# Measuring Violence Against Children: A COSMIN Systematic Review of the Psychometric and Administrative Properties of Adult Retrospective Self-report Instruments on Child Abuse and Neglect

**DOI:** 10.1177/15248380221145912

**Published:** 2023-01-25

**Authors:** Bridget Steele, Lakshmi Neelakantan, Janina Jochim, Lynn M. Davies, Mark Boyes, Hannabeth Franchino-Olsen, Michael Dunne, Franziska Meinck

**Affiliations:** 1 University of Oxford, UK; 2 University of Edinburgh, UK; 3 Curtin University, Perth, WA, Australia; 4 Hue University, Vietnam; 5 Queensland University of Technology, Australia; 6 North-West University, Vanderbijlpark, South Africa; 7 University of the Witwatersrand, Johannesburg, South Africa

**Keywords:** violence against children, child abuse, measurement, psychometric properties, systematic review

## Abstract

Valid, meaningful, and reliable adult retrospective measures of violence against children (VAC) are essential for establishing the prevalence, risk factors, and long-term effects of VAC. We aim to summarize the available evidence on the psychometric properties of adult retrospective VAC measures and to provide evidence-based recommendations for appropriate measure selection. We searched six electronic databases and gray literature for studies that report on the development, content validity, or psychometric properties of adult retrospective child abuse and neglect measures for this review (PROSPERO: CRD4201706). We used the 2018 Consensus-based Standards for the selection of health Measurement Instruments (COSMIN) criteria to evaluate each included study and measure. We assessed if measures included questions on frequency or severity, the perpetrator, or the location of the violence, and noted the administrative practicalities for each instrument such as length, readability, available translations, and cost to access. We identified 288 studies and 77 measures. The quality of evidence ranged from “low” to “high,” depending on the measure and the psychometric properties assessed. The measures with the most robust evidence available across multiple contexts are the: ACE and ACE-IQ; FBQ and FBQ-U; CTQ and CTQ-SF; and ICAST-R. This review shows the strengths and weaknesses of retrospective VAC measures. The substantial evidence presented in this review can be used by researchers to make psychometrically sound decisions for measurement selection which should be supported by extensive piloting and adaptation to the respective local context.

## Background

Violence against children (VAC) is conceptualized to include physical abuse, emotional abuse, sexual abuse, neglect, and exposure to domestic violence. Measuring the prevalence, nature, risk factors, and impacts of childhood violence victimization is essential for prevention and response efforts (World Health Oraganization, 2014).

### Measuring VAC

There are multiple methods that can be used to measure VAC, including child self-report, adult retrospective self-report, parent report, police or agency records, or researcher observations (Meinck et al., 2016). Self-report measures (adult and child) ask participants about their experiences with VAC. They are often the most feasible; they are less likely to suffer from underreporting and are more cost effective, when compared to other measurement types, and are thus commonly used in both prevalence surveys and studies on intervention effectiveness (Meinck et al., 2016).

### Importance of Adult Retrospective Self-Report Tools for Measuring VAC

Adult retrospective self-report measures are particularly useful for establishing a holistic understanding of childhood violence (Lyon, 2014). These measures ask people 18 years and older about the violence they experienced as children. Children may not be ready or able to disclose their violence victimization until adulthood and as a result, prevalence estimates are often lower in childhood surveys than in adult surveys (Easton, 2012; McGuire & London, 2020). Adult retrospective self-report measures are also useful for understanding the impact of VAC on older generations, for whom contemporaneous research into childhood violence was rare. Further, adult retrospective self-report measures, in most cases, are more practical, timely, and ethical to implement, when compared to child self-report measures, given that participants are over the age of 18 and as such they are widely used in surveys on the prevalence and outcomes of VAC (CDC, 2020; Mathews et al., 2021; Witt et al., 2017).

### Psychometric Properties of Adult Retrospective Self-Report Measures

**Figure 1.**
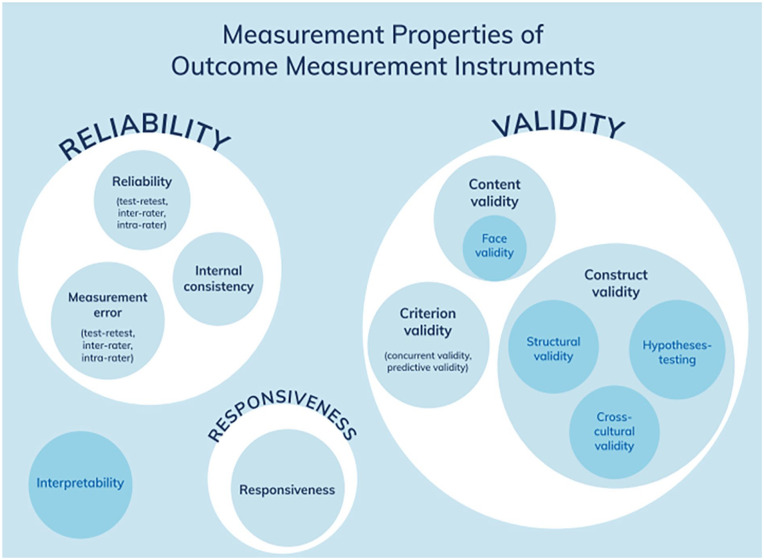
Measurement properties of outcome measurement instruments. *Source*. Mokkink et al. (2010).

Reliable and valid measures for adult retrospective reports of VAC are important to the understanding of prevalence, severity, and long-term implications of childhood violence (Mathews et al., 2020; Meinck et al., 2016). The reliability and validity of measures is established though the evaluation of psychometric (or measurement) properties. The international Consensus-based Standards for the selection of health Measurement Instruments (COSMIN) provides a taxonomy that identifies the key measurement properties that should be evaluated for any measurement instrument used in any application (see Figure 1). However, selecting an appropriate tool for measuring VAC can be difficult and daunting, especially for researchers who are new to the field or to psychometric assessment. The sheer number of measures used in the literature, combined with the inconsistent use and reporting of psychometric testing, often proves overwhelming to those looking to select a measure for research (Finkelhor et al., 2013; Moore et al., 2015).

Several related papers have partially summarized the psychometric properties of retrospective measures of child abuse, but these reviews were either regionally specific (Ritacco & Suffla, 2012; Satapathy et al., 2017), did not include self-report measures (Heinze & Grisso 1996; Stowman & Donohye, 2005), or are not systematic in nature (Burgermeister, 2007; Hardt & Rutter, 2004; Hulme, 2004; Tonmyr et al., 2011; Walsh et al., 2004). Further, these reviews did not apply the Consensus-based standards for the selection of health measurement instruments (COSMIN). COSMIN offers a standardized and rigorous methodology for assessing measurement properties; both the quality of a study and the quality of the measure are evaluated.

### Applying COSMIN Criteria

The application of COSMIN is useful for improving the evidence base on VAC measures and assisting researchers and practitioners in the field to choose suitable instruments. Selecting a poor, inappropriate, or untested instrument to measure VAC has the potential to produce misleading or even inaccurate data. It is essential to assess the scope and quality of VAC measures using agreed-upon rigorous criteria so that researchers and clinicians can make timely and informed decisions.

### Research Objectives

Given the plethora of retrospective self-report VAC survey tools, the task of selecting an appropriate measure to use in studies with adult populations is challenging. This systematic review therefore has five aims. First, to systematically search the literature and both identify and describe all adult retrospective self-report measures for VAC which are supported by psychometric evidence. Second, to assess the content validity of measures when possible. Third, to evaluate the psychometric properties of each instrument using COSMIN standards. Fourth, to describe the scope of the measure (e.g., type(s) of violence to be measured, the recall period, or frequency and severity), and the logistical elements such as mode of application, number of items, readability, available translations, and accessibility or cost of the measure. Lastly, to offer recommendations, based on the evidence summarized and assessed in this review, to researchers intending to measure VAC.

### Existing Evidence

There are four related reviews that have been conducted on the psychometric properties of child abuse measures using COSMIN criteria. Yoon et al. (2021a) examined content validity of parent or caregiver reports of child maltreatment, Yoon et al. (2021b) considered other psychometric properties of parent or caregiver reports of child maltreatment, and Yoon et al. (2022), evaluated the responsiveness of parent- or caregiver-reported child maltreatment measures for interventions. The first, Yoon et al. (2021a) review found 15 content validity studies covering 15 different instruments. The quality of included studies was rated poor; however, the content validity of the instruments was rated sufficient. The second, Yoon et al. (2021b) study found 25 studies reporting on the psychometric properties of 15 different instruments. The third, Yoon et al. (2022) study found 69 articles reporting on the responsiveness of 15 measures. The authors rated the methodological quality of included studies as adequate but found that the psychometric properties of the instruments were overall inadequate or insufficient. These three reviews examined measures of *parent or caregiver* self-reported behavior while the current study examines individuals’ self-reported experiences of violence experienced as a child.

Saini et al. (2019) conducted a review that assessed all types of self-reported child abuse and neglect (parent, child, and adult retrospective). This review only included articles published prior to July 2016 and only searched three electronic databases. Saini et al. (2019) included 68 studies that reported on the psychometric properties of 52 instruments. This review found a wide discrepancy in the methodological quality across studies and inconsistent evidence on the psychometric properties for instruments. There was little to no evidence for several measurement properties such as measurement error, criterion validity, and cross-cultural validity. As a result, the authors conclude that they are unable to recommend an instrument. Our review provides a more comprehensive and up-to-date search of the literature while also discussing the practical and administrative considerations for instrument selection.

## Methods

The research team used PRISMA guidelines (Moher et al., 2009) to conduct a global systematic review of the psychometric properties for self-report measures of child abuse (PROSPERO 2017: CRD42017062251). The review has yielded two articles. The first article, Meinck et al. (2022) summarizes the psychometric properties of *child self-report* measures. This present article focuses on *adult retrospective self- report* measures. In-depth reporting of methods can be found in Meinck et al. (2022).

### Literature Search

We searched the following electronic databases in April 2017 and in October 2020: PsycINFO, MEDLINE, Embase, Global Health, ProQuest, and Social Sciences Citation Index. The search terms used for each database are listed in the Supplemental Material. We also searched key journals in the field directly, contacted experts, and conducted backward and forward citation searching to identify articles that were not retrieved in the electronic databases searches.

### Selection Criteria for Eligible Studies

We conducted title and abstract screening using the following inclusion criteria. First, the measure or part of the measure assessed retrospective self-report experiences of VAC (physical, emotional, or sexual abuse, and neglect). Tools that retrospectively ask about self-report experiences of VAC are measures designed to ask people 18 years and older about their exposure to violence when they were children. Specifically, these tools measure physical and emotional abuse perpetrated by a caregiver, parent, or similar person in a position of power (e.g., teacher), sexual abuse perpetrated by anyone, neglect in the home, and witnessing domestic violence in the home. Tools that measure experiences of bullying were not included. However, measures such as the ICAST and ACE which included items on household dysfunction or peer victimization were included as these items appeared alongside other types of abuse that met the inclusion criteria for this review.

Second, the measure was completed by adults aged 18 and above. Third, the study developed or validated an adult retrospective VAC measure, or reported data on the content validity, construct validity (structural validity, hypothesis testing with other VAC measures, or measures of depression, anxiety, suicidal ideation, self-harm, delinquency, criminal activity, or revictimization, measurement invariance/cross cultural validity, or criterion validity), reliability (test-retest reliability, internal consistency, or measurement error), or concordance. Lastly, the paper was published in English.

We excluded the COSMIN psychometric property “responsiveness,” which assesses a measurement tool’s ability to detect change. Due to the nature of retrospective self-report measures, all respondents in our included studies were adults, so we would not expect to see a change in reports of child abuse. Common reasons for excluding studies were if they: (1) only assessed other forms of VAC such as peer violence or exposure to community violence and not VAC inflicted by adults on children within a school or home environment; (2) used single items or a non-standardized measure to assess VAC; or (3) measured attitudes and perceptions of VAC rather than abusive events.

At the beginning of the screening process, two research team members double-screened 10% of eligible titles and abstracts. When there was discrepancy or disagreement, studies were discussed by the author team in relation to the established inclusion and exclusion criteria and the research question. With guidance from the senior author, consensus on the final decision was reached in all cases. This process allowed the team to add clarity and transparency to the study selection. We kept an ongoing list of measures that we defined as retrospective VAC measures and noted our reasoning as to why we decided to include each measure. This documentation was shared with the authorship team to allow for consistency in study selection. The remaining titles and abstracts were then screened by members of the authorship team. This process was repeated for the full text screening.

### Data Extraction

We extracted the following data from each included study: the measure(s) used, the intended construct for measurement, the administration method, accessibility, the study population, the number of participants, the participant demographics, the country and setting, and the language. We then extracted details on each psychometric property: content validity, construct validity (structural validity, cross-cultural validity, measurement error, criterion validity, and hypothesis testing), reliability (test-retest reliability, internal consistency (within, not across subscales), measurement error), and concordance. Lastly, we extracted information on whether the measure asked about the perpetrator, the location, or the frequency and severity of the VAC experienced.

### Methodological and Measurement Quality Assessment

We applied the COSMIN guidelines for each study and measure included in this review (Mokkink et al., 2018). First, we assessed the methodological quality of included studies. Second, we assessed included study results for each psychometric property. The data extraction forms used to apply ratings to the studies, psychometric properties, and the measures is included in the Supplemental Material. These forms were slightly adapted from the COSMIN data extraction forms. Third, we summarized and conducted a quality grading of the evidence. Lastly, we assessed the contents and the practical administrative properties of each measure to enhance the applicability and practicality of the findings of this review. Methodology and measurement quality were assessed by two raters for each study. The four steps are outlined in detail below.

### Step 1: Assessment of Methodological Quality of Included Studies

We used the COSMIN Risk of Bias Checklist to assess the methodological quality of each study (Mokkink et al., 2018). For each study reporting on the content validity of a measure we assessed the measure for *relevance* (all items in the measure were relevant to the construct of interest, the context, and the population), *comprehensiveness* (all key aspects of the construct were included), and *comprehensibility* (the words and phrases used in each measure could be understood by the target population) (Terwee et al., 2018). Content validity refers to the extent to which the contents of an instrument represent the construct that it intends to measure and is assessed through professional and participant-led evaluation of the relevance, comprehensiveness, and comprehensibility of each item in an instrument (Terwee et al., 2018).

We then rated the measurement of psychometric properties in each included study as “very good,” “adequate,” “doubtful,” or “inadequate” based on the appropriateness of the study design, the methodology used, and the statistical analyses employed. The final score was equal to the lowest rating across the checklist and an inadequate rating was given if there was not enough information to assess the quality of the study (Mokkink et al., 2018; Prinsen et al., 2018). A detailed description of the criteria used can be found in the Supplemental Material.

### Step 2: Assessment of Study Results for Each Psychometric Property

We then assessed the results for each included study using the COSMIN guidance on good measurement properties (Prinsen et al., 2018). We applied the COSMIN guidance for the outcomes of content validity studies and the results of each psychometric property evaluation in each individual study. We rated a psychometric property as sufficient (+) if appropriate statistical procedures were used with appropriately high scores; insufficient (−) if appropriate statistical procedures were not used or if scores were low; indeterminate (?) if information was not fully reported or hypotheses were missing; or inconsistent (±) if some hypotheses were met but others were not (Terwee et al., 2018). A detailed description of the criteria used to apply these ratings can be found in the Supplemental Material.

### Step 3: Summary and Quality Grading of the Evidence

Next, we summarized all assessments of psychometric properties and methodological quality, conducted at the individual study level, for each measure using COSMIN criteria (Prinsen et al., 2018). We examined the quality of the overall body of evidence for each measure’s content validity and psychometric properties using the Grading of Recommendations Assessment, Development, and Evaluation (GRADE), applying the following ratings: “high,” “moderate,” “low,” or “very low” (Prinsen et al., 2018). We then rated the overall results for each instrument’s psychometric properties as sufficient (+), insufficient (−), indeterminate (?), or inconsistent (±).

### Step 4: Assessment of Practical Administrative Properties

We assessed if each instrument included questions on perpetrators, location of abuse, disclosure of abuse, frequency and severity of abuse, or burden of participation. We also extracted information on the time and mode of administration, readability, and accessibility of each measure.

## Results

### Description of Studies

**Figure 2.**
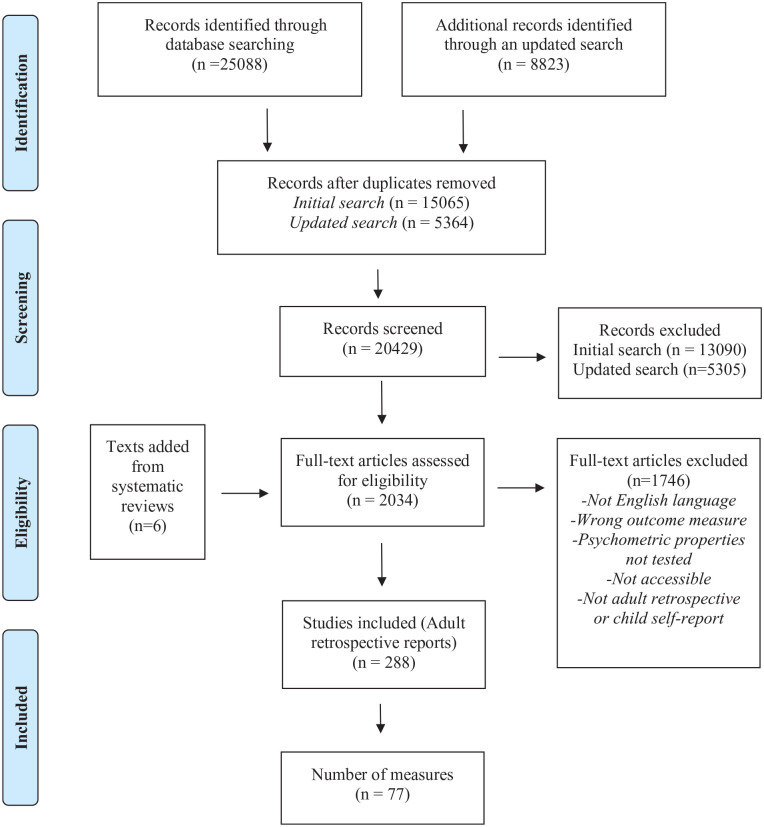
PRISMA flow-diagram. *Source.* Moher et al. (2009)

The search yielded 33,911 articles. After duplicates were removed, 20,429 articles remained. We identified 2,034 full-text articles for eligibility during the title and abstract screening. We determined 288 articles that reported data from 77 adult retrospective self-report measures qualified for inclusion. A list of all studies and measures included in this review is included in the Supplemental Materials. Included articles were published between 1988 and 2020. See the first table in the Supplemental Material for detailed descriptions of each measure. The study selection flow chart is shown in Figure 2.

### Description of Measures

Most measures (*n* = 48) assess multiple forms of VAC. Some measures (*n* = 29) assess a single form of violence; four measures only assess physical violence, five measures only assess psychological violence, eighteen measures only assess sexual violence, and two measures only assess neglect. There are 67 original measures and ten modified versions of an original measure included in this review. For example, ACE was included as an original measure, but the ACE-ASF, ACE-BRFSS, ACE-IQ, and ACE-S were also included as they were reported on in studies as modified measures. There are also measures which were originally developed for current use with parents and children, and not retrospective self-report with adults, such as the CTS-PC.

All measures were implemented with a sample of adults (18+ years). There was discrepancy among how each measure defined and measured “childhood,” with the following recall periods: before age 18 (25 measures); before age 17 (three measures); before age 16 (two measures); before age 15 (one measure); before age 14 (one measure); before age 13 (one measure); before age 7 (one measure); between ages 8–18 (one measure); between ages 5 and 16 (one measure); “childhood” (17 measures), and “lifetime” (14 measures).

The measures with the most evidence available in terms of number of studies using the measure and reporting on psychometric properties are: the CTQ-SF (87 studies across 6 of 9 psychometric properties); ACE (39 studies across 3 of 9 psychometric properties); CATS (17 studies across 5 of 9 psychometric properties); the CTQ (11 studies across 6 of 9 psychometric properties); CTS-PC (9 studies across 3 of 9 psychometric properties); CECA (9 studies across 5 of 9 psychometric properties). Yet, there are no development or content validity studies for any of these measures.

### Overview of Measurement Properties

Hypothesis testing was the most assessed psychometric property among included studies (*n* = 252), followed by internal consistency (*n* = 143), structural validity (*n* = 52), other forms of reliability (including test-retest reliability, intra-rater, and inter-rater reliability) (*n* = 48), and concordance (*n* = 28). Very few studies reported on cross-cultural validity (*n* = 12), and criterion validity (*n* = 8). Only 12 studies reported on content validity. No studies investigated measurement error, so this property was not reported on. The below results are structured using the COSMIN taxonomy of measurement properties that rely on the domains of validity and reliability. Concordance is also assessed and falls outside these two domains. Detailed reporting on the psychometric properties in each included study can be found in the Supplemental Material.

### Validity

Validity refers to the extent to which an outcome measure is appropriate for the construct it is designed to measure. This domain encompasses the following measurement properties: content validity; construct validity (which includes structural validity, hypothesis testing and cross-cultural validity); and criterion validity.

#### Content validity

Content validity refers to the extent to which the wording within an instrument reflects the construct it intends to measure (Mokkink et al., 2018).

Development study quality was assessed in five studies for three different measures (ICAST-R, CMQ, PTI), all receiving low ratings of either inadequate or doubtful. Content validity was assessed in 12 studies across ten measures: ICAST-R; CMQ; CCMI; DDI; FBQ; FBQ-U; ACE-IQ; TLEQ; PTI; MMCSA.

The overall methodological quality of each study was poor as most studies did not include respondents from the target population when assessing content validity or did not provide information on the respondents. Relevance was investigated in only two studies involving the target population (measures: ICAST-R; ACE-IQ) and six studies with professionals (measures: ICAST-R; CMQ; FBQ-U; FBQ; TLEQ; MMCSA). Only one study received a rating of “very good” (Quinn et al., 2017; ACE-IQ) and one received a rating of “adequate” the remainder were rated “doubtful.” Comprehensiveness was assessed in two studies involving the target population (measures: ICAST-R; DDI) and in five studies with professionals (measures: ICAST-R; CMQ; FBQ; TLEQ; MMCSA). All studies were rated “doubtful” except for one (Villarroel et al., 2012; TLEQ) which received a rating of “adequate.” Comprehensibility was assessed in five studies involving the target population (measures: ICAST-R; CCMI; FBQ-U; ACE-IQ) but only one study (Quinn et al., 2017; ACE-IQ) received a rating of “very good” with the rest receiving a rating of “doubtful.”

The quality of content validation for each included study was also assessed. Then the quality of content validity for each measure was assessed: The ICAST-R, CMQ, CCMI, FBQ-U, FBQ, ACE-IQ, TLEQ, and MMCSA were rated sufficient for relevance, while the DDI and PTI received an indeterminate rating. The CMQ, DDI, FBQ, and TLEQ were rated sufficient for comprehensiveness while the ICAST-R received an inconsistent rating due to differences in ratings across included studies, and the CCMI and the PTI received an indeterminate rating. The FBQ-U and ACE-IQ were rated sufficient for their comprehensibility, while ICAST-R was again rated as inconsistent due to differences in ratings in included studies. The CMQ, CCMI, DDI, and the PTI received an indeterminate rating. A summary of the criteria for evaluating content validity and a summary of the evidence for content validity can be found in the Supplemental Material.

#### Structural validity

Structural validity refers to the extent to which the scores of a measure “are an adequate reflection of the dimensionality of the construct to be measured” (Mokkink et al., 2018).

Structural validity was tested for 35 measures in 52 different studies. The methodological quality for the majority of studies testing structural validity was adequate or very good (*n* = 44) with only eight studies having doubtful or inadequate methodological quality. Studies that conducted confirmatory factor analysis with a sample that was seven times the number of items received a rating of very good. The highest rating that a study using exploratory factor analysis could receive was adequate. Studies were often rated doubtful or inadequate if the sample size was not sufficient according to COSMIN guidance.

The evidence for the quality of the psychometric properties in each study was not as strong: 24 studies were rated as sufficient, 11 studies were rated as insufficient, 16 studies were rated as indeterminate, and 1 study was rated as inconsistent. Indeterminate ratings were given when the information needed to receive a rating was not reported. A sufficient rating was received if the results of the Confirmatory or Exploratory Factor Analysis or Rasch/Item Response Theory were above the cut-off set by COSMIN. Insufficient ratings were given when the results did not meet the sufficient criteria. The overall quality of structural validity was sufficient for the following fourteen measures: ACE-ASF; AC-BRFSS; ACE-S; CES; CTA; CTQ; FBQ-SF; JVQ; NS; TAQ; TEC; VEQ; PMR; SAQ1 (see Supplemental Material). All other measures either received an “insufficient,” “indeterminate,” or “inconsistent” rating. The overall quality of the body of evidence for instruments with “sufficient” structural validity ranged from moderate to high.

#### Cross-cultural validity

Cross cultural validity assesses whether a translated or culturally adapted measure performs differently than the original measure (Mokkink et al., 2018).

Twelve studies across six measures (ACE-BRFSS; CATS; FBQ-U; CARTS; CTQ-SF; MNBS) were rated for cross-cultural validity. The ACE, CARTS, CTQ-SF, FBQ, and MNBS were all rated “sufficient” with “moderate” to “high” quality of evidence. The CATS was rated indeterminate with very low quality of evidence.

Studies were rated as sufficient if in a multiple group factor analysis, no differences were found between group factors such as age, gender, or language, or if there was no important Differential Item Functioning for group factors. The methodological quality of studies was determined by sample characteristics and sample size, as well as the analysis approach.

#### Hypothesis testing

Hypothesis testing assesses the extent to which the outcome scores of a VAC measure are consistent with other VAC measures, or measures of depression, anxiety, suicidal ideation, self-harm, delinquency, criminal activity, or revictimization. Hypothesis testing can also be used to assess whether differences in outcome scores between groups are consistent with pre-existing relationships (Mokkink et al., 2018).

Hypothesis testing was the most common psychometric property assessed in included studies. We found 250 studies conducting hypothesis testing for a total of 68 measures. The study quality was rated very good for 132 studies, adequate for 63 studies, doubtful for 41 studies, and inadequate for 14 studies, due to the use of inappropriate statistical methods. The quality of the psychometric property was sufficient for most studies (*n* = 207) with only 21 studies being rated as insufficient, 8 studies inconsistent, and 14 studies indeterminate. The overall quality of hypothesis testing was rated “sufficient” for 52 measures. The quality of the body of evidence conducting hypothesis testing ranged from very low to high, with most studies (53) assessed as high quality.

#### Criterion validity

Criterion validity assesses whether the scores of an adapted or shortened version of a measure are consistent with the original version of the measure (Mokkink et al., 2018).

Eight studies across eight measures assessed criterion validity. Six of the eight measures were rated “sufficient” for overall quality of the psychometric property (ACE; CES; CEVQ-SF; ETI; ETI-SF; SAQ1), with the body of evidence being high for ACE, CES, CEVQ-SF, and ETI-SF. The SAQ1 was rated “moderate” for quality of evidence available and the ETI had low quality of evidence due to methodological flaws in the analysis.

### Reliability

An assessment of reliability determines whether outcome scores for a measurement remain consistent when that measurement is applied in different conditions (e.g., different time points) (Mokkink et al., 2018). This domain includes the following measurement properties: internal consistency, and; test-retest, inter-rater, and intra-rater reliability.

#### Internal consistency

Internal consistency is a type of reliability and refers to “the degree of interrelatedness among items” in a measure (Mokkink et al., 2018). Internal consistency was measured for 49 different measures in 143 studies. The methodological quality of studies testing internal consistency was high with 113 studies rated very good or adequate and only 30 studies rated as doubtful or inadequate. The quality of internal consistency within these studies was high with 67 studies rated sufficient, 43 inconsistent, 21 insufficient, and 12 indeterminate. Most measures (32) where internal consistency was measured were given an overall “sufficient” rating for internal consistency while only seven measures were rated insufficient. The quality of the body of evidence for measures rated “sufficient” for internal consistency was high with 30 of the 32 measures receiving a moderate or high rating as the majority of studies reported consistency using Cronbach’s alpha.

#### Test-retest, inter-rater, and intra-rater reliability

We found forty-eight studies investigating these forms of reliability for 35 measures. The methodological quality of studies testing for reliability varied across the four ratings due to inconsistent use of appropriate statistical methods and lack of reporting on study design: very good (4), adequate (8), doubtful (28), and inadequate (8). The quality of reliability within these studies was rated from insufficient (*n* = 3) to sufficient (*n* = 24), with 4 studies having an inconsistent rating and 17 studies having an indeterminate rating. Overall, five of the 35 measures were rated “sufficient.” The quality of evidence for each measure ranged from being “very low” (5 measures) and “low” (16 measures) to “moderate” (9 measures) and “high” (2 measures) (see Supplemental Material for more details). The following measures were rated “sufficient” with a “moderate” to “high” quality of evidence: CTI; ETI; ETI-SF; SNFI; TLEQ; SAEQ; ITEC, and SAQ1.

### Concordance

Concordance examines the extent to which VAC scores correspond depending on whether the participant themselves or another person (e.g., therapist or health professional) completes the questionnaire, or when responses on self-report measures are compared to registry data (e.g., child protective service or police records) (Mokkink et al., 2018). We found 27 studies and 16 measures assessing concordance. The quality ratings of the studies were inadequate (3), doubtful (2), moderate (7) and very good (15). The quality of the concordance was rated sufficient in 12 studies and inconsistent (*n* = 7) or insufficient (*n* = 8) in the remaining studies. Of the 16 measures rated for concordance, 9 were given an overall rating of “sufficient” (ACE-IQ; CAMI; CECA; CTQ; NorAQ; TAQ; TEQ; FCSES; RSAIS), 5 were rated insufficient, and 2 were inconsistent. The quality of the evidence ranged from moderate to high, except for the TEQ, which was rated low.

### Additional Considerations

Researchers attempting to select an appropriate retrospective measure of child abuse should consider the psychometric properties and the contents of the instrument to see if they align with the aims and objectives of a research study. For more information on what criteria was applied to assign the ratings please see the detailed data extraction form with COSMIN guidance imbedded in the Supplemental Material. Each measure reported on in this study focuses on and features different elements of child abuse. Table 1 summarizes non-psychometric contents of instruments that could be important when selecting a measure. This information is also outlined in detail in the first table within the Supplemental Material.

**Table 1.** Non-Psychometric Considerations for the Contents of Instruments.

Does the Measure Ask About?	Description of Questions	Studies
Frequency of abuse	Response options that ask about frequency often include scales from never to often.	44 measures
Severity of abuse	Severity can be measured by asking about the impact of the abuse or if injuries were sustained.	43 measures
Disclosure of abuse/ response to this disclosure	Questions that ask the victim if they told anyone about the abuse, who they told, or what the response was from the person or people they disclosed to.	13 measures: CEVQ; CTI; CTA; CCMI; ICAST-R; JVQ; NorAQ; ESEQ; FCSES; HEAS; MMCSA; SAEQ; SHQ
Perpetrator characteristics	Questions that seek to understand who the perpetrator was in terms of their relationship to the victim survivor. For example, was the perpetrator a family member or stranger.	52 measures
Location of abuse	Questions that ask where the child abuse occurred. For example, some questions may ask if the abuse occurred at home or at school.	2 measures: JVQ; NorAQ
Trivialization	Questions that assess how participants present their experiences of violence. For example, an item on a scale might state “my childhood was fantastic.”	5 measures: APK; CTQ; CTQ-SF; APDI-EC; APDI-LC
Burden of participation	Questions that ask participants how difficult they found answering the questions, if they regret participating, or if they would participate again in a similar study.	2 measures: CEVQ; JVQ

### Practical administrative properties

In addition to considering what is included in each child abuse measure and how that differs across measures, it is important to consider practical and administrative elements such as the length of time to administer it, available translations, or the cost of accessing the measures. Table 2 summarizes these properties with tables in the Supplemental Material providing detail across all included measures.

**Table 2.** Practical Administrative Properties.

Property	Description of Property	Studies
Accessibility	Some measures are freely available for use while other measures require a fee to use them.	4 measures have a fee to use: CTQ-SF; CTS-PC; ETI; ETI-SF.
Administration time	The time it takes for participants to take the survey.	Only 24 measures reported the estimated time to complete the instrument. Measures ranged from 5 to 90 min.
Handbook or scoring guide	Measures are at times accompanied by user guides that assist researchers in implementing the measure and scoring results.	26 measures had a user handbook available to assist researchers with the implementation and scoring of the instrument.
Readability	The Flesch reading score was calculated for each measure when possible. Scores closer to 100 indicate that the questionnaire could be more accessible and easier to read.	This property should be considered when implementing questionnaires in contexts where there are low literacy levels. A score above 80 indicates a reading level of age 15.
Languages/translations	Some measures have been translated into different languages.	55 measures are only available in English and 25 measures have non-English translations.

## Discussion

We systematically reviewed the English language evidence for the psychometric properties of adult retrospective self-report VAC measures. We thoroughly assessed the quality of included studies, rigorously evaluated the psychometric properties of measures, and described each measure’s administrative properties, to provide evidence-based recommendations for determining what measure to use in retrospective research on VAC. Our critical findings are outlined in Table 3.

**Table 3.** Critical Findings.

● Our search identified 288 articles reporting on 77 adult retrospective child abuse and neglect measures.
● Hypothesis testing and internal consistency were the most frequently assessed psychometric properties in included studies, likely due to their ease of application.
● Only 12 studies across ten measures reported on the most important measurement property, content validity. Further, the methodological quality of these studies was mostly poor.
● The measures with the most robust evidence available across multiple contexts are: ACE and ACE-IQ; FBQ and FBQ-U; CTQ and CTQ-SF; and; ICAST-R. The quality of these instruments has been proven across multiple psychometric properties, with evidence on content validity available for ACE-IQ, FBQ-U, and the ICAST-R.

Our study builds on existing research conducted by Mathews et al. (2020). Their review of national prevalence studies on child abuse and neglect suggests that retrospective measurements of VAC have demonstrated strengths but may have compromised validity due to motivational factors, memory biases, and poorly worded questions. Mathews et al. find that retrospective measures that use behaviorally specific questions with good content validity in surveys with representative samples can adequately estimate the prevalence of VAC. Our review helps to contextualize these finding, by providing transparent detailed information on why certain VAC measurement tools can be considered more valid and reliable than others.

Through our search and screening process, we identified 288 articles describing a total of 77 adult retrospective child abuse and neglect measures. These 77 measures include 10 measures that have been substantively adapted from their previous versions to be shorter in length or to add an additional construct. These adapted measures were included in the review as independent measures. For example, we included the CTQ and the CTQ-SF as distinct measures as we felt it would be inaccurate to compare psychometric properties across these two measures.

### Strengths and Limitations of COSMIN Guidelines

We applied the 2018 COSMIN criteria for study and measure evaluation. These criteria are rigorous and time consuming and required the research team to undergo multiple evaluation steps at both the study and measure level.

There are several advantages from the application of COSMIN guidelines in systematic reviews. First, the process for evaluating psychometric properties is comprehensive and standardized. This is also found by Kwok et al. (2021) who conducted a review on the strengths and limitations of the COSMIN tools. Further, the freely available guidance and instruction on how to identify and rate a psychometric property was thorough and detailed.

We also identified limitations to relying on the COSMIN guidelines. First, we found the “worst case counts” rule, for assessing the methodological quality of studies, can give too much weight to a minor methodological concern. This has also been found by Wittkowski et al. (2020) in their assessment of parent-infant attachment measures and Meinck et al. (2022) who report on VAC child self-report measures. Given the purpose of the COSMIN guidelines to help researchers choose an acceptable measure for research, we believe that more discretion should be given to researchers for assessing methodological quality of studies. Second, the psychometric property of internal reliability might not be relevant for certain VAC measurement tools and therefore low internal consistency scores for VAC measures should be interpreted with caution. For example, items on a physical violence scale can be so different (e.g., smacking or spanking compared to burning or assaulting with a weapon) that it is not useful or possible to establish an underlying construct (Straus & Hamby, 1997; Straus et al., 1998). As recommended by Meinck et al. (2022) there is a need for subject matter experts to be part of the review team when applying COSMIN criteria. Third, the COSMIN guidelines, in their current form, are time-consuming to apply. Given the large number of studies and measures covered in this review, we learned that a more streamlined process for reporting and synthesizing study quality, psychometric quality, and the overall evidence available for each measure is needed. Adapting and then transferring the COSMIN rating forms to be compatible with Excel allowed our team to clearly report assessments of each psychometric property for each study without compromising the comprehensiveness and the integrity of the COSMIN guidelines.

### Content Validity

Content validity is widely considered to be the most important measurement property, yet we only found 12 studies to assess the content validity of a total of 10 measures (Terwee et al., 2018). This could be explained by the fact that content validity is difficult to measure (Terwee et al., 2018). The process of establishing content validity can be time consuming, iterative, and may be overlooked by researchers who want to select a pre-existing validated measure. The lack of available evidence on content validity could also be explained by reporting gaps; Meinck et al. (2022) argue that pilot testing of measures is frequently conducted but rarely reported.

Of the studies included in the review that do report on content validity, the methodological quality was overall poor. Very few studies provided adequate details regarding the process of consultations with professionals or the target population; many studies simply just stated that the consultations took place. Further, no study assessed all three components of content validity (relevance, comprehensiveness, and comprehensibility). The ACE-IQ was the only measure to receive high ratings for methodological and content validity quality assessment. The ACE-IQ content validity study provides an example to future researchers looking to expand the availability and quality of evidence on content validity of adult retrospective child abuse and neglect measures (Quinn et al., 2017).

### Considerations Relating to Other Psychometric Properties

Certain psychometric properties were measured more frequently than others. Hypothesis testing and internal consistency were, by far, the most assessed psychometric properties in included studies. Potential reasons for this are: (1) Hypothesis testing was often assessed in studies that were not explicitly evaluating the psychometric properties of a measure and were designed primarily to establish the relationship between child abuse and an adverse outcome, and; (2) There is no specialty software that is required for measuring internal consistency and researchers will have all the information necessary to statistically evaluate internal consistency at multiple points, even if this is not a pre-planned analysis.

Other psychometric properties were less commonly reported on (concordance and cross-cultural validity). This is not surprising; concordance is resource intensive to implement as it requires researchers to implement two different instruments for one construct and commonly involves a comparison of an instrument to professional clinical judgment.

The lack of cross-cultural validity studies is concerning given the wide-spread global use of some child abuse measures and the comparisons frequently drawn for prevalence estimates between different countries. Untested assumptions regarding cultural invariance in the measures may also compromise studies of associations between VAC and risk factors or putative outcomes across contexts (Flake & Fried, 2020). The diversity of the settings in which an instrument has been tested must be considered when using the same instrument in a new social or cultural context. Additionally, there was limited evidence considering if and how gender identity relates to VAC measurement.

### Overall Evidence per Instrument

Over 60% of the 288 included studies report psychometric properties for one of these measures: CTQ-SF; ACE; CTQ; CTS-PC; CECA. Yet, none of these measures have been assessed for content validity and only ACE and CECA measures are freely accessible. Further, only the CTQ-SF has been evaluated rigorously for cross-cultural validity. This shows that despite the prolific use of certain measures, fundamental understanding of certain psychometric properties is lacking.

We did however find seven promising measures that have robust psychometric properties: ACE and ACE-IQ; FBQ and FBQ-U; CTQ and CTQ-SF; and ICAST-R. These measures stand out because of the considerable amount of high-quality work that has been conducted to better understand their validity and reliability. For example, Asmundson and Afifi (2019) consolidate and describe in detail existing efforts to assess the psychometric properties of the ACE measure. Further, the CTQ is so pervasively used that our review was able to gather sufficient evidence to evaluate its psychometric qualities.

We encourage researchers to use the tables in the Supplemental Material to look across all psychometric properties for a certain measure to assess (a) the extent of psychometric evaluation for each measure and (b) the quality of each psychometric property. Further, certain psychometric properties may be more pertinent to certain research studies than others and this should be considered when choosing a measure. For example, cross-cultural validity may not be necessary if a measure is used in the context in which it was developed but it could be crucial if it were implemented in a new country or culture.

### Considerations Relating to Contents of Measures and Practical Administration

We summarize contents of each measure that may be useful for researchers. Items measuring the frequency or severity of the abuse, perpetrator characteristics, location of abuse, disclosure, or participant burden may be important for individual researchers depending on the aims and objectives of each study. We also summarize practical administrative elements of each measure such as the length of the measure, readability, available translations, and cost. Moving beyond psychometric properties, certain measures may be more suitable for certain studies. A framework for considering these properties in relation to measure selection and study goals can be found in Meinck et al. (2022).

## Strengths and Limitations

The strengths of this review lie in its comprehensiveness and rigor including the scope of the search, that all studies were double screened, the large number of articles screened and included, and the application of the COSMIN 2018 criteria. Finally, our synthesis of instrument contents and administration practicalities enhances the usefulness of this review by offering researchers a way to identify the most appropriate measure for their use. Implications for practice are outlined in Table 4.

**Table 4.** Review Implications.

Practice	● We encourage researchers to critically evaluate each measure included in this review to ensure it meets the objectives of their research project; different measures could be more valid and reliable depending on the context and aims of a study.
Policy	● Decision makers should assess prevalence estimates of VAC based on an understanding of the strengths and limitations of the measures used to estimate prevalence.
	● Additionally, it is important to understand how the type of measure used in research can influence outcomes and conclusions.
Research	● Extensive qualitative work, including cognitive interviewing and focus group discussions, should be carried out with target populations, and reported on in detail, to establish content validity.
	● Measures must be assessed for cross-cultural validity before being implemented across geographies to ensure that results can be appropriately compared across population groups.

There are some limitations in the scope of the review. First, we only included studies published in the English language, excluding possibly relevant studies published in other languages that could have contributed to and altered our findings and recommendations. Second, we excluded studies that used an adult retrospective VAC measure but did not report on psychometric properties. Fourth, we included any study that measured VAC retrospectively and with a self-report tool in this review, even if the authors selected an inappropriate measure to do so. In cases where authors employed a measure that was not intended to measure retrospective self-report VAC, the psychometric properties were sub-optimal. Lastly, adult retrospective self-report measures are limited as they typically measure lifetime (before the age of 18) exposure to violence and are subject to recall bias. For example, respondents may experience memory decay or distortion which could impact their responses. Additionally, there is evidence that people have trouble dating events which can make it difficult for participants to answer follow-up question about incidents of abuse (Janssen et al., 2006). Further adult retrospective self-report measures do not allow researchers to monitor current trends due to the time frame of questions asked.

## Conclusion

This is the first systematic review employing the 2018 COSMIN criteria to assess psychometric properties of adult retrospective self-report VAC measures. We found a total of 67 unique measures and 10 modified versions. The methodological quality of the evidence for most measures was low and scant, especially evidence on content validity which is the most important psychometric property. Both the methodological quality of studies and evidence on each psychometric property were lacking. The large number of studies in this review shows that many adult retrospective measures have been developed and are used with little systematic evidence regarding their psychometric properties. Future research should work to establish the reliability and validity of the most commonly employed adult retrospective VAC instruments.

## Supplemental Material

sj-docx-1-tva-10.1177_15248380221145912 – Supplemental material for Measuring Violence Against Children: A COSMIN Systematic Review of the Psychometric and Administrative Properties of Adult Retrospective Self-report Instruments on Child Abuse and Neglect Click here for additional data file. Supplemental material, sj-docx-1-tva-10.1177_15248380221145912 for Measuring Violence Against Children: A COSMIN Systematic Review of the Psychometric and Administrative Properties of Adult Retrospective Self-report Instruments on Child Abuse and Neglect by Bridget Steele, Lakshmi Neelakantan, Janina Jochim, Lynn M. Davies, Mark Boyes, Hannabeth Franchino-Olsen, Michael Dunne and Franziska Meinck in Trauma, Violence, & Abuse

sj-docx-2-tva-10.1177_15248380221145912 – Supplemental material for Measuring Violence Against Children: A COSMIN Systematic Review of the Psychometric and Administrative Properties of Adult Retrospective Self-report Instruments on Child Abuse and Neglect Click here for additional data file. Supplemental material, sj-docx-2-tva-10.1177_15248380221145912 for Measuring Violence Against Children: A COSMIN Systematic Review of the Psychometric and Administrative Properties of Adult Retrospective Self-report Instruments on Child Abuse and Neglect by Bridget Steele, Lakshmi Neelakantan, Janina Jochim, Lynn M. Davies, Mark Boyes, Hannabeth Franchino-Olsen, Michael Dunne and Franziska Meinck in Trauma, Violence, & Abuse

sj-docx-3-tva-10.1177_15248380221145912 – Supplemental material for Measuring Violence Against Children: A COSMIN Systematic Review of the Psychometric and Administrative Properties of Adult Retrospective Self-report Instruments on Child Abuse and Neglect Click here for additional data file. Supplemental material, sj-docx-3-tva-10.1177_15248380221145912 for Measuring Violence Against Children: A COSMIN Systematic Review of the Psychometric and Administrative Properties of Adult Retrospective Self-report Instruments on Child Abuse and Neglect by Bridget Steele, Lakshmi Neelakantan, Janina Jochim, Lynn M. Davies, Mark Boyes, Hannabeth Franchino-Olsen, Michael Dunne and Franziska Meinck in Trauma, Violence, & Abuse

sj-docx-4-tva-10.1177_15248380221145912 – Supplemental material for Measuring Violence Against Children: A COSMIN Systematic Review of the Psychometric and Administrative Properties of Adult Retrospective Self-report Instruments on Child Abuse and Neglect Click here for additional data file. Supplemental material, sj-docx-4-tva-10.1177_15248380221145912 for Measuring Violence Against Children: A COSMIN Systematic Review of the Psychometric and Administrative Properties of Adult Retrospective Self-report Instruments on Child Abuse and Neglect by Bridget Steele, Lakshmi Neelakantan, Janina Jochim, Lynn M. Davies, Mark Boyes, Hannabeth Franchino-Olsen, Michael Dunne and Franziska Meinck in Trauma, Violence, & Abuse

sj-docx-5-tva-10.1177_15248380221145912 – Supplemental material for Measuring Violence Against Children: A COSMIN Systematic Review of the Psychometric and Administrative Properties of Adult Retrospective Self-report Instruments on Child Abuse and Neglect Click here for additional data file. Supplemental material, sj-docx-5-tva-10.1177_15248380221145912 for Measuring Violence Against Children: A COSMIN Systematic Review of the Psychometric and Administrative Properties of Adult Retrospective Self-report Instruments on Child Abuse and Neglect by Bridget Steele, Lakshmi Neelakantan, Janina Jochim, Lynn M. Davies, Mark Boyes, Hannabeth Franchino-Olsen, Michael Dunne and Franziska Meinck in Trauma, Violence, & Abuse

sj-docx-6-tva-10.1177_15248380221145912 – Supplemental material for Measuring Violence Against Children: A COSMIN Systematic Review of the Psychometric and Administrative Properties of Adult Retrospective Self-report Instruments on Child Abuse and Neglect Click here for additional data file. Supplemental material, sj-docx-6-tva-10.1177_15248380221145912 for Measuring Violence Against Children: A COSMIN Systematic Review of the Psychometric and Administrative Properties of Adult Retrospective Self-report Instruments on Child Abuse and Neglect by Bridget Steele, Lakshmi Neelakantan, Janina Jochim, Lynn M. Davies, Mark Boyes, Hannabeth Franchino-Olsen, Michael Dunne and Franziska Meinck in Trauma, Violence, & Abuse
