# Supplementary material for: Measuring Violence Against Children: A COSMIN Systematic Review of the Psychometric and Administrative Properties of Adult Retrospective Self-report Instruments on Child Abuse and Neglect
Source: Trauma Violence Abuse. 2023 Jan 25;25(1):183–96. doi: 10.1177/15248380221145912 (PMC10666516; doi:10.1177/15248380221145912)
Supplement: sj-docx-1-tva-10.1177_15248380221145912 – Supplemental material for Measuring Violence Against Children: A COSMIN Systematic Review of the Psychometric and Administrative Properties of Adult Retrospective Self-report Instruments on Child Abuse and Neglect [file sj-docx-1-tva-10.1177_15248380221145912.docx]

Included studies:

1. Adamson, J. L. (1997). *Predicting attachment and violence in relationships: An investigation across three generations*. The University of Nebraska-Lincoln.
2. Afifi, T. O., Ford, D., Gershoff, E. T., Merrick, M., Grogan-Kaylor, A., Ports, K. A., ... & Bennett, R. P. (2017). Spanking and adult mental health impairment: The case for the designation of spanking as an adverse childhood experience. *Child Abuse & Neglect*, *71*, 24-31.
3. Allen, B., Cramer, R. J., Harris, P. B., & Rufino, K. A. (2013). Borderline personality symptomatology as a mediator of the link between child maltreatment and adult suicide potential. *Archives of suicide research*, *17*(1), 41-51.
4. Allen Jr, V. C., Myers, H. F., & Williams, J. K. (2014). Depression among Black bisexual men with early and later life adversities. *Cultural Diversity and Ethnic Minority Psychology*, *20*(1), 128.
5. Allen, J. G., Coyne, L., & Huntoon, J. (1998). Trauma pervasively elevates Brief Symptom Inventory profiles in inpatient women. *Psychological Reports*, *83*(2), 499-513.
6. Allen, B. (2011). Childhood psychological abuse and adult aggression: The mediating role of self-capacities. *Journal of interpersonal violence*, *26*(10), 2093-2110.
7. Ammerman, R. T., Peugh, J. L., Teeters, A. R., Putnam, F. W., & Van Ginkel, J. B. (2016). Child maltreatment history and response to CBT treatment in depressed mothers participating in home visiting. *Journal of interpersonal violence*, *31*(5), 774-791.
8. Anda, R. F., Whitfield, C. L., Felitti, V. J., Chapman, D., Edwards, V. J., Dube, S. R., & Williamson, D. F. (2002). Adverse childhood experiences, alcoholic parents, and later risk of alcoholism and depression. *Psychiatric services*, *53*(8), 1001-1009.
9. Antonopoulou, Z., Konstantakopoulos, G., Tzinieri-Coccosis, M., & Sinodino, C. (2017). Rates of childhood trauma in a sample of university students in Greece: The Greece version of the Early Trauma Inventory--Self Report. *Psychiatriki*.
10. Andreopoulos, S. H. (2002). *Childhood trauma and childhood sexual abuse as predictor variables of adult attachment and adult partner violence in a population of substance abusing men*. Adelphi University, The Institute of Advanced Psychological Studies.
11. Arata, C. M., & Lindman, L. (2002). Marriage, child abuse, and sexual revictimization. *Journal of Interpersonal Violence*, *17*(9), 953-971.
12. Arnow, B. A., Blasey, C. M., Hunkeler, E. M., Lee, J., & Hayward, C. (2011). Does gender moderate the relationship between childhood maltreatment and adult depression?. *Child maltreatment*, *16*(3), 175-183.
13. Bahk, Y. C., Jang, S. K., Choi, K. H., & Lee, S. H. (2017). The relationship between childhood trauma and suicidal ideation: role of maltreatment and potential mediators. *Psychiatry investigation*, *14*(1), 37.
14. Bailer, J., Witthöft, M., Wagner, H., Mier, D., Diener, C., & Rist, F. (2014). Childhood maltreatment is associated with depression but not with hypochondriasis in later life. *Journal of psychosomatic research*, *77*(2), 104-108.
15. Bailey, H. N., DeOliveira, C. A., Wolfe, V. V., Evans, E. M., & Hartwick, C. (2012). The impact of childhood maltreatment history on parenting: A comparison of maltreatment types and assessment methods. *Child abuse & neglect*, *36*(3), 236-246.
16. Banducci, A. N., Hoffman, E. M., Lejuez, C. W., & Koenen, K. C. (2014). The impact of childhood abuse on inpatient substance users: Specific links with risky sex, aggression, and emotion dysregulation. *Child abuse & neglect*, *38*(5), 928-938.
17. Banducci, A. N., Hoffman, E., Lejuez, C. W., & Koenen, K. C. (2014). The relationship between child abuse and negative outcomes among substance users: Psychopathology, health, and comorbidities. *Addictive behaviors*, *39*(10), 1522-1527.
18. Basto-Pereira, M., Miranda, A., Ribeiro, S., & Maia, Â. (2016). Growing up with adversity: From juvenile justice involvement to criminal persistence and psychosocial problems in young adulthood. *Child abuse & neglect*, *62*, 63-75.
19. Bellis, M. A., Lowey, H., Leckenby, N., Hughes, K., & Harrison, D. (2014). Adverse childhood experiences: retrospective study to determine their impact on adult health behaviours and health outcomes in a UK population. *Journal of public health*, *36*(1), 81-91.
20. Bernet, C. Z., & Stein, M. B. (1999). Relationship of childhood maltreatment to the onset and course of major depression in adulthood. *Depression and anxiety*, *9*(4), 169-174.
21. Bernstein, D. P., Stein, J. A., Newcomb, M. D., Walker, E., Pogge, D., Ahluvalia, T., ... & Zule, W. (2003). Development and validation of a brief screening version of the Childhood Trauma Questionnaire. *Child abuse & neglect*, *27*(2), 169-190.
22. Beutel, M. E., Tibubos, A. N., Klein, E. M., Schmutzer, G., Reiner, I., Kocalevent, R. D., & Brähler, E. (2017). Childhood adversities and distress-The role of resilience in a representative sample. *PloS one*, *12*(3), e0173826.
23. Bifulco, A., Moran, P. M., Baines, R., Bunn, A., & Stanford, K. (2002). Exploring psychological abuse in childhood: II. Association with other abuse and adult clinical depression. *Bulletin of the Menninger Clinic*, *66*(3), 241-258.
24. Birchfield, J. M. (1996). *Comparison of levels of dissociation and history of child sexual abuse between single-and multiple-incident rape victims*. Fordham University.
25. Bizzarro, M. R. (2003). *Lifetime patterns of maternal substance abuse as a predictor of child maltreatment and child developmental outcomes*. Institute for Clinical Social Work (Chicago).
26. Boillat, C., Schwab, N., Stutz, M., Pflueger, M. O., Graf, M., & Rosburg, T. (2017). Neuroticism as a risk factor for child abuse in victims of childhood sexual abuse. *Child abuse & neglect*, *68*, 44-54.
27. Bonevski, D., Novotni, A., Raleva, M., & Naumovska, A. (2012). Childhood abuse and level of manifested anxiety in adult patients with anxiety disorder. *Maced J Med Sci*, *5*(1), 94-98.
28. Bradley, R., Schwartz, A. C., & Kaslow, N. J. (2005). Posttraumatic stress disorder symptoms among low‐income, African American women with a history of intimate partner violence and suicidal behaviors: Self‐esteem, social support, and religious coping. *Journal of Traumatic Stress: Official Publication of The International Society for Traumatic Stress Studies*, *18*(6), 685-696.
29. Bremner, J. D., Vermetten, E., & Mazure, C. M. (2000). Development and preliminary psychometric properties of an instrument for the measurement of childhood trauma: the Early Trauma Inventory. *Depression and anxiety*, *12*(1), 1-12.
30. Bremner, J. D., Bolus, R., & Mayer, E. A. (2007). Psychometric properties of the early trauma inventory–self report. *The Journal of nervous and mental disease*, *195*(3), 211.
31. Briere, J., Godbout, N., & Runtz, M. (2012). The Psychological Maltreatment Review (PMR): Initial reliability and association with insecure attachment in adults. *Journal of Aggression, Maltreatment & Trauma*, *21*(3), 300-320.
32. Briere, J., Madni, L. A., & Godbout, N. (2016). Recent suicidality in the general population: multivariate association with childhood maltreatment and adult victimization. *Journal of interpersonal violence*, *31*(18), 3063-3079.
33. Brockie, T. N., Dana-Sacco, G., Wallen, G. R., Wilcox, H. C., & Campbell, J. C. (2015). The relationship of adverse childhood experiences to PTSD, depression, poly-drug use and suicide attempt in reservation-based Native American adolescents and young adults. *American journal of community psychology*, *55*(3-4), 411-421.
34. Bruskas, D. (2012). *Adverse childhood experiences and psychosocial well-being of adult women formerly in foster care as children*. University of Washington.
35. Bruskas, D., & Tessin, D. H. (2013). Adverse childhood experiences and psychosocial well-being of women who were in foster care as children. *The Permanente Journal*, *17*(3), e131.
36. Burnette, C. E., Roh, S., Lee, K. H., Lee, Y. S., Newland, L. A., & Jun, J. S. (2017). A comparison of risk and protective factors related to depressive symptoms among American Indian and Caucasian older adults. *Health & Social Work*, *42*(1), e15-e23.
37. Burton, D. L. (2008). An exploratory evaluation of the contribution of personality and childhood sexual victimization to the development of sexually abusive behavior. *Sexual Abuse*, *20*(1), 102-115.
38. Cabrera, O. A., Hoge, C. W., Bliese, P. D., Castro, C. A., & Messer, S. C. (2007). Childhood adversity and combat as predictors of depression and post-traumatic stress in deployed troops. *American journal of preventive medicine*, *33*(2), 77-82.
39. Cammack, A. L., Hogue, C. J., Drews-Botsch, C. D., Kramer, M. R., Pearce, B. D., Knight, B. T., ... & Newport, D. J. (2016). Test-retest reliability of retrospective self-reported maternal exposure to childhood abuse and neglect. *Archives of women's mental health*, *19*(2), 415-421.
40. Campbell, J. A., Walker, R. J., & Egede, L. E. (2016). Associations between adverse childhood experiences, high-risk behaviors, and morbidity in adulthood. *American journal of preventive medicine*, *50*(3), 344-352.
41. Chandraratne, N. K., Fernando, A. D., & Gunawardena, N. (2018). Cultural adaptation, translation and validation of the ISPCAN Child Abuse Screening Tool–Retrospective Version (ICAST-R) for young adults in Sri Lanka. *Child abuse & neglect*, *84*, 11-22.
42. Chapman, D. P., Whitfield, C. L., Felitti, V. J., Dube, S. R., Edwards, V. J., & Anda, R. F. (2004). Adverse childhood experiences and the risk of depressive disorders in adulthood. *Journal of affective disorders*, *82*(2), 217-225.
43. Charak, R., Byllesby, B. M., Roley, M. E., Claycomb, M. A., Durham, T. A., Ross, J., ... & Elhai, J. D. (2016). Latent classes of childhood poly-victimization and associations with suicidal behavior among adult trauma victims: Moderating role of anger. *Child Abuse & Neglect*, *62*, 19-28.
44. Chegeni, M., Haghdoost, A., Shahrbabaki, M. E., Shahrbabaki, P. M., & Nakhaee, N. (2020). Validity and reliability of the Persian version of the Adverse Childhood Experiences Abuse Short Form. *Journal of education and health promotion*, *9*.
45. Chung, E. K., Mathew, L., Elo, I. T., Coyne, J. C., & Culhane, J. F. (2008). Depressive symptoms in disadvantaged women receiving prenatal care: the influence of adverse and positive childhood experiences. *Ambulatory Pediatrics*, *8*(2), 109-116.
46. Claridge, A. M., Lettenberger-Klein, C. G., Farineau, H. M., Wojciak, A. S., & McWey, L. M. (2014). Maternal history of victimization and adolescent behaviors: protective function of relationship quality among at-risk mother-adolescent dyads. *Journal of Family Violence*, *29*(5), 473-482.
47. Clemmons, J. C. (2004). *Multiple forms of child maltreatment and abuse-specific characteristics: Relationships to psychological adjustment*. The University of Nebraska-Lincoln.
48. Cooper, C. D. (1995). *Childhood sexual abuse and depressive symptoms in a lesbian population: An exploratory study* (Doctoral dissertation, University of Southern California).
49. Corvo, K. N. (1993). *Attachment and violence in the families of origin of domestically violent men* (Doctoral dissertation, Case Western Reserve University).
50. Craig, T. K. J., & Hodson, S. (2000). Homeless youth in London: II. Accommodation, employment and health outcomes at 1 year. *Psychological medicine*, *30*(1), 187-194.
51. Craig, M. C. (2002). *The mediating role of safety, trust, and esteem in explaining the relationship between childhood sexual abuse and adult revictimization*. Texas A&M University.
52. Cristofaro, S. L., Cleary, S. D., Wan, C. R., Broussard, B., Chapman, C., Haggard, P. J., ... & Compton, M. T. (2013). Measuring trauma and stressful events in childhood and adolescence among patients with first-episode psychosis: initial factor structure, reliability, and validity of the Trauma Experiences Checklist. *Psychiatry research*, *210*(2), 618-625.
53. D'Angelo, K. J. (2006). *The role of reflective functioning as a protective factor in survivors of trauma* (Doctoral dissertation, Long Island University, The Brooklyn Center).
54. De Ravello, L., Abeita, J., & Brown, P. (2008). Breaking the cycle/mending the hoop: adverse childhood experiences among incarcerated American Indian/Alaska Native women in New Mexico. *Health care for women international*, *29*(3), 300-315.
55. Demare, D. (2000). Examining long-term correlates of psychological, physical, and sexual childhood maltreatment, validation of the childhood maltreatment questionnaire.
56. Dietrich, A. (2003). Characteristics of child maltreatment, psychological dissociation, and somatoform dissociation of Canadian inmates. *Journal of Trauma & Dissociation*, *4*(1), 81-100.
57. DiLillo, D., Fortier, M. A., Hayes, S. A., Trask, E., Perry, A. R., Messman-Moore, T., ... & Nash, C. (2006). Retrospective assessment of childhood sexual and physical abuse: A comparison of scaled and behaviorally specific approaches. *Assessment*, *13*(3), 297-312.
58. DiLillo, D., Hayes-Skelton, S. A., Fortier, M. A., Perry, A. R., Evans, S. E., Moore, T. L. M., ... & Fauchier, A. (2010). Development and initial psychometric properties of the Computer Assisted Maltreatment Inventory (CAMI): A comprehensive self-report measure of child maltreatment history. *Child Abuse & Neglect*, *34*(5), 305-317.
59. DiLillo, D., Hayes-Skelton, S. A., Fortier, M. A., Perry, A. R., Evans, S. E., Moore, T. L. M., ... & Fauchier, A. (2010). Development and initial psychometric properties of the Computer Assisted Maltreatment Inventory (CAMI): A comprehensive self-report measure of child maltreatment history. *Child Abuse & Neglect*, *34*(5), 305-317.
60. Dong, M., Anda, R. F., Felitti, V. J., Dube, S. R., Williamson, D. F., Thompson, T. J., ... & Giles, W. H. (2004). The interrelatedness of multiple forms of childhood abuse, neglect, and household dysfunction. *Child abuse & neglect*, *28*(7), 771-784.
61. Dovran, A., Winje, D., Øverland, S. N., Breivik, K., Arefjord, K., Dalsbø, A. S., ... & Waage, L. (2013). Psychometric properties of the Norwegian version of the Childhood Trauma Questionnaire in high‐risk groups. *Scandinavian journal of psychology*, *54*(4), 286-291.
62. Ast, E. (2006). *The Development and Validation of the Ast Physical Discipline Inventory-2006 (APDI)* (Doctoral dissertation, Alliant International University, California School of Professional Psychology, Fresno).
63. Berger, A. M., Knutson, J. F., Mehm, J. G., & Perkins, K. A. (1988). The self-report of punitive childhood experiences of young adults and adolescents. *Child Abuse & Neglect*, *12*(2), 251-262.
64. Bernstein, D. P., Stein, J. A., & Handelsman, L. (1998). Predicting personality pathology among adult patients with substance use disorders: Effects of childhood maltreatment. *Addictive behaviors*, *23*(6), 855-868.
65. Bifulco, A., Bernazzani, O., Moran, P. M., & Jacobs, C. (2005). The childhood experience of care and abuse questionnaire (CECA. Q): validation in a community series. *British Journal of Clinical Psychology*, *44*(4), 563-581.
66. Carr, A., Dooley, B., Fitzpatrick, M., Flanagan, E., Flanagan-Howard, R., Tierney, K., ... & Egan, J. (2010). Adult adjustment of survivors of institutional child abuse in Ireland. *Child Abuse & Neglect*, *34*(7), 477-489.
67. Dube, S. R., Williamson, D. F., Thompson, T., Felitti, V. J., & Anda, R. F. (2004). Assessing the reliability of retrospective reports of adverse childhood experiences among adult HMO members attending a primary care clinic. *Child abuse & neglect*.
68. Dunne, M. P., Zolotor, A. J., Runyan, D. K., Andreva-Miller, I., Choo, W. Y., Dunne, S. K., ... & Youssef, R. (2009). ISPCAN Child Abuse Screening Tools Retrospective version (ICAST-R): Delphi study and field testing in seven countries. *Child abuse & neglect*, *33*(11), 815-825.
69. Dunn, S. E. (2009). *Childhood maltreatment and adult post-traumatic stress disorder symptomatology in abused, suicidal, low-income, African American women: A moderated mediational model*. Georgia State University.
70. Dunn, C. E. (1994). *Child abuse trauma: Incidence, interpersonal manifestations, and other correlates in university counseling center clients* (Doctoral dissertation, University of Kansas).
71. Duran, B., Malcoe, L. H., Sanders, M., Waitzkin, H., Skipper, B., & Yager, J. (2004). Child maltreatment prevalence and mental disorders outcomes among American Indian women in primary care. *Child Abuse & Neglect*, *28*(2), 131-145.
72. Durrett, C., Trull, T. J., & Silk, K. (2004). Retrospective measures of childhood abuse: Concurrent validity and reliability in a nonclinical sample with borderline features. *Journal of personality disorders*, *18*(2), 178-192.
73. Edwards, C. E. (1997). *A test of theory: Four traumagenic dynamics of sexual abuse in a population of adult female sexual abuse survivors*. University of Missouri-Kansas City.
74. Edwards, V. J., Anda, R. F., Nordenberg, D. F., Felitti, V. J., Williamson, D. F., & Wright, J. A. (2001). Bias assessment for child abuse survey: factors affecting probability of response to a survey about childhood abuse. *Child abuse & neglect*.
75. Eldeeb, N., Halileh, S., Alyafei, K. A., Ghandour, R., Dargham, S., Giacaman, R., ... & Mian, M. (2016). Child discipline in Qatar and Palestine: A comparative study of ICAST-R. *Child abuse & neglect*, *61*, 63-72.
76. Epperson, C. N., Sammel, M. D., Bale, T. L., Kim, D. R., Conlin, S., Scalice, S., ... & Freeman, E. W. (2017). Adverse childhood experiences and risk for first-episode major depression during the menopause transition. *The Journal of clinical psychiatry*, *78*(3), 0-0.
77. Ehrenthal, J. C., Schauenburg, H., Wagner, F. E., Dinger, U., & Volz, M. (2020). Development and Evaluation of the Questionnaire for the Assessment of Adverse and Protective Childhood Experiences (APC). *Psychiatrische Praxis*, *47*(4), 207-213.
78. Fauchier, A., & Straus, M. A. (2010). Psychometric properties of the Adult-Recall form of the Dimensions of Discipline Inventory. *Manuscript under review*.
79. Ferrari, A. M. (2002). The impact of culture upon child rearing practices and definitions of maltreatment. *Child abuse & neglect*, *26*(8), 793-813.
80. Festinger, T., & Baker, A. (2010). Prevalence of recalled childhood emotional abuse among child welfare staff and related well-being factors. *Children and Youth Services Review*, *32*(4), 520-526.
81. Figueiredo, B., Bifulco, A., Paiva, C., Maia, A., Fernandes, E., & Matos, R. (2004). History of childhood abuse in Portuguese parents. *Child Abuse & Neglect*, *28*(6), 669-682.
82. Fink, L. A. (1995). *The Childhood Trauma Interview: Development, coding reliability and validity* (Doctoral dissertation, New York University).
83. Fiore, J. (1997). *Factors related to chemical dependence in adult offspring of alcoholic parents*. Fordham University.
84. Fisher, H. L., Craig, T. K., Fearon, P., Morgan, K., Dazzan, P., Lappin, J., ... & Morgan, C. (2011). Reliability and comparability of psychosis patients’ retrospective reports of childhood abuse. *Schizophrenia Bulletin*, *37*(3), 546-553.
85. Fitzhenry, M., Harte, E., Carr, A., Keenleyside, M., O’Hanrahan, K., White, M. D., ... & Browne, S. (2015). Child maltreatment and adult psychopathology in an Irish context. *Child abuse & neglect*, *45*, 101-107.
86. Font, S. A., & Maguire-Jack, K. (2016). Pathways from childhood abuse and other adversities to adult health risks: The role of adult socioeconomic conditions. *Child abuse & neglect*, *51*, 390-399.
87. Ford, D. C., Merrick, M. T., Parks, S. E., Breiding, M. J., Gilbert, L. K., Edwards, V. J., ... & Thompson, W. W. (2014). Examination of the factorial structure of adverse childhood experiences and recommendations for three subscale scores. *Psychology of violence*, *4*(4), 432.
88. Forde, D. R., Baron, S. W., Scher, C. D., & Stein, M. B. (2012). Factor structure and reliability of the childhood trauma questionnaire and prevalence estimates of trauma for male and female street youth. *Journal of interpersonal violence*, *27*(2), 364-379.
89. Fosse, G. K., & Holen, A. (2007). Reported maltreatment in childhood in relation to the personality features of Norwegian adult psychiatric outpatients. *The Journal of nervous and mental disease*, *195*(1), 79-82.
90. Fuchs, A., Möhler, E., Resch, F., & Kaess, M. (2016). Sex-specific differences in adrenocortical attunement in mothers with a history of childhood abuse and their 5-month-old boys and girls. *Journal of neural transmission*, *123*(9), 1085-1094.
91. Fuller-Thomson, E., Roane, J. L., & Brennenstuhl, S. (2016). Three types of adverse childhood experiences, and alcohol and drug dependence among adults: An investigation using population-based data. *Substance Use & Misuse*, *51*(11), 1451-1461.
92. Gahm, G. A., Lucenko, B. A., Retzlaff, P., & Fukuda, S. (2007). Relative impact of adverse events and screened symptoms of posttraumatic stress disorder and depression among active duty soldiers seeking mental health care. *Journal of Clinical Psychology*, *63*(3), 199-211.
93. Galea, M. (2012). Studying the incremental validity of family environment among Maltese university students with past mental trauma. *Pastoral Psychology*, *61*(2), 211-220.
94. Gartland, D., Woolhouse, H., Giallo, R., McDonald, E., Hegarty, K., Mensah, F., ... & Brown, S. J. (2016). Vulnerability to intimate partner violence and poor mental health in the first 4-year postpartum among mothers reporting childhood abuse: an Australian pregnancy cohort study. *Archives of women's mental health*, *19*(6), 1091-1100.
95. Gauble, K. (2010). *An examination of childhood sexual abuse and substance use initiation in Appalachia*. University of Kentucky.
96. Gerdner, A., & Allgulander, C. (2009). Psychometric properties of the swedish version of the childhood trauma Questionnaire—Short form (CTQ-SF). *Nordic journal of psychiatry*, *63*(2), 160-170.
97. Gerra, G., Manfredini, M., Somaini, L., Milano, G., Ciccocioppo, R., & Donnini, C. (2016). Perceived parental care during childhood, ACTH, cortisol and nicotine dependence in the adult. *Psychiatry research*, *245*, 458-465.
98. Gerra, G., Somaini, L., Manfredini, M., Raggi, M. A., Saracino, M. A., Amore, M., ... & Donnini, C. (2014). Dysregulated responses to emotions among abstinent heroin users: Correlation with childhood neglect and addiction severity. *Progress in neuro-psychopharmacology and biological psychiatry*, *48*, 220-228.
99. Gil, A., Gama, C. S., de Jesus, D. R., Lobato, M. I., Zimmer, M., & Belmonte-de-Abreu, P. (2009). The association of child abuse and neglect with adult disability in schizophrenia and the prominent role of physical neglect. *Child abuse & neglect*, *33*(9), 618-624.
100. Giovanelli, A., Reynolds, A. J., Mondi, C. F., & Ou, S. R. (2016). Adverse childhood experiences and adult well-being in a low-income, urban cohort. *Pediatrics*, *137*(4).
101. Goldsmith, R. E. (2004). *Physical and emotional health effects of betrayal trauma: A longitudinal study of young adults*. University of Oregon.
102. Grewal-Sandhu, P. K. (2008). Hope theory: A framework for understanding the relation between childhood maltreatment and adult suicidal action.
103. Grilo, C. M., White, M. A., Masheb, R. M., Rothschild, B. S., & Burke-Martindale, C. H. (2006). Relation of childhood sexual abuse and other forms of maltreatment to 12-month postoperative outcomes in extremely obese gastric bypass patients. *Obesity surgery*, *16*(4), 454-460.
104. Grossman, G. A. (1997). *Effects of childhood trauma and dissociation with substance abusing adolescents*. The Wright Institute.
105. Hörberg, N., Kouros, I., Ekselius, L., Cunningham, J., Willebrand, M., & Ramklint, M. (2019). Early Trauma Inventory Self-Report Short Form (ETISR-SF): validation of the Swedish translation in clinical and non-clinical samples. *Nordic journal of psychiatry*, *73*(2), 81-89.
106. Hahn, A. M., Simons, R. M., & Simons, J. S. (2016). Childhood maltreatment and sexual risk taking: the mediating role of alexithymia. *Archives of sexual behavior*, *45*(1), 53-62.
107. Harmer, A. L., Sanderson, J., & Mertin, P. (1999). Influence of negative childhood experiences on psychological functioning, social support, and parenting for mothers recovering from addiction. *Child abuse & neglect*, *23*(5), 421-433.
108. Harrington, D., Zuravin, S., DePanfilis, D., Ting, L., & Dubowitz, H. (2002). The neglect scale: Confirmatory factor analyses in a low-income sample. *Child maltreatment*, *7*(4), 359-368.
109. Hayashi, Y., Okamoto, Y., Takagaki, K., Okada, G., Toki, S., Inoue, T., ... & Yamawaki, S. (2015). Direct and indirect influences of childhood abuse on depression symptoms in patients with major depressive disorder. *BMC psychiatry*, *15*(1), 1-8.
110. Hernandez, A., Gallardo-Pujol, D., Pereda, N., Arntz, A., Bernstein, D. P., Gaviria, A. M., ... & Gutiérrez-Zotes, J. A. (2013). Initial validation of the Spanish childhood trauma questionnaire-short form: factor structure, reliability and association with parenting. *Journal of interpersonal violence*, *28*(7), 1498-1518.
111. Higgins, D. J., & McCabe, M. P. (2001). The development of the comprehensive child maltreatment scale. *Journal of family studies*, *7*(1), 7-28.
112. Hocking, E. C., Simons, R. M., & Surette, R. J. (2016). Attachment style as a mediator between childhood maltreatment and the experience of betrayal trauma as an adult. *Child abuse & neglect*, *52*, 94-101.
113. Honkalampi, K., Honkalampi, K., Hintikka, J., Haatainen, K., Koivumaa-Honkanen, H., Tanskanen, A., & Viinamäki, H. (2005). Adverse childhood experiences, stressful life events or demographic factors: which are important in women's depression? A 2-year follow-up population study. *Australian & New Zealand Journal of Psychiatry*, *39*(7), 627-632.
114. Hughes, K., Lowey, H., Quigg, Z., & Bellis, M. A. (2016). Relationships between adverse childhood experiences and adult mental well-being: results from an English national household survey. *BMC public health*, *16*(1), 1-11.
115. Hung, G. C. L., Caine, E. D., Fan, H. F., Huang, M. C., & Chen, Y. Y. (2013). Predicting Suicide Attempts among Treatment‐Seeking Male Alcoholics: An Exploratory Study. *Suicide and Life‐Threatening Behavior*, *43*(4), 429-438.
116. Hyman, S. M., Garcia, M., Kemp, K., Mazure, C. M., & Sinha, R. (2005). A gender specific psychometric analysis of the early trauma inventory short form in cocaine dependent adults. *Addictive behaviors*, *30*(4), 847-852.
117. Jeon, J. R., Lee, E. H., Lee, S. W., Jeong, E. G., Kim, J. H., Lee, D., & Jeon, H. J. (2012). The early trauma inventory self report-short form: psychometric properties of the Korean version. *Psychiatry investigation*, *9*(3), 229.
118. Jackson, T. L. (2004). *The relationships among childhood psychological maltreatment, adult psychological functioning, dating abuse, and schema*. The University of North Dakota.
119. Jacobs, A. E. (1998). *Childhood Abuse and Incarceration: A Comparison between Recidivists and Nonrecidivists in an East Oakland Community*. California Institute of Integral Studies.
120. Jangam, K., Devasthali, G., Tansa, K. A., Raj, A., & Muralidharan, K. (2016). Childhood abuse in adult women with unipolar depression compared to healthy women: Data from a tertiary care centre in India. *Asian journal of psychiatry*, *22*, 138-139.
121. Kalebić Jakupčević, K., & Ajduković, M. (2011). Risk factors of child physical abuse by parents with mixed anxiety-depressive disorder or posttraumatic stress disorder. *Croatian medical journal*, *52*(1), 25-34.
122. Jennissen, S., Holl, J., Mai, H., Wolff, S., & Barnow, S. (2016). Emotion dysregulation mediates the relationship between child maltreatment and psychopathology: A structural equation model. *Child abuse & neglect*, *62*, 51-62.
123. Jeong, B., Lee, S. W., Lee, J. S., Yoo, J. H., Kim, K. W., Cho, S., ... & Choi, J. (2015). The psychometric properties of the Korean version of the verbal abuse questionnaire in university students. *Psychiatry investigation*, *12*(2), 190.
124. Jewkes, R. K., Dunkle, K., Nduna, M., Jama, P. N., & Puren, A. (2010). Associations between childhood adversity and depression, substance abuse and HIV and HSV2 incident infections in rural South African youth. *Child abuse & neglect*, *34*(11), 833-841.
125. Jewkes, R., Nduna, M., Jama-Shai, N., Chirwa, E., & Dunkle, K. (2016). Understanding the relationships between gender inequitable behaviours, childhood trauma and socio-economic status in single and multiple perpetrator rape in rural South Africa: structural equation modelling. *PLoS one*, *11*(5), e0154903.
126. Kaier, E., Cromer, L. D., Davis, J. L., & Strunk, K. (2015). The relationship between adverse childhood experiences and subsequent health complaints in elite athletes. *Journal of Child & Adolescent Trauma*, *8*(2), 83-92.
127. Kazeem, O. T. (2015). A validation of the adverse childhood experiences scale in Nigeria. *Research on Humanities and Social Sciences*, *5*(11), 18-23.
128. Kalemeera, A. (2007). Cross-cultural assessment of child maltreatment: Adapting the family background questionnaire with Ugandan students.
129. Karabatsos, G. (1997). The Sexual Experiences Survey: interpretation and validity. *Journal of Outcome Measurement*, *1*(4), 305-328.
130. Karakus, Ö. (2012). Childhood abuse and attachment styles of adolescents.
131. Karanović, J., Ivković, M., Jovanović, V. M., Šviković, S., Pantović-Stefanović, M., Brkušanin, M., ... & Savić-Pavićević, D. (2017). Effect of childhood general traumas on suicide attempt depends on TPH2 and ADARB1 variants in psychiatric patients. *Journal of neural transmission*, *124*(5), 621-629.
132. Karatekin, C., & Ahluwalia, R. (2020). Effects of adverse childhood experiences, stress, and social support on the health of college students. *Journal of interpersonal violence*, *35*(1-2), 150-172.
133. Karos, K., Niederstrasser, N., Abidi, L., Bernstein, D. P., & Bader, K. (2014). Factor structure, reliability, and known groups validity of the German version of the Childhood Trauma Questionnaire (Short-form) in Swiss patients and nonpatients. *Journal of child sexual abuse*, *23*(4), 418-430.
134. Kennedy, M. A. (2003). *Identifying child abuse: a structural equation modeling analysis of history of abuse and westernization on perceptions of abuse among Asian-descent and European-descent students* (Doctoral dissertation, University of British Columbia).
135. Kent, A., & Waller, G. (1998). The impact of childhood emotional abuse: An extension of the child abuse and trauma scale. *Child Abuse & Neglect*, *22*(5), 393-399.
136. Kim, Y. H. (2017). Associations of adverse childhood experiences with depression and alcohol abuse among Korean college students. *Child abuse & neglect*, *67*, 338-348.
137. Kim, D., Bae, H., Han, C., Oh, H. Y., & MacDonald, K. (2013). Psychometric properties of the Childhood Trauma Questionnaire-Short Form (CTQ-SF) in Korean patients with schizophrenia. *Schizophrenia research*, *144*(1-3), 93-98.
138. Kim, D., Park, S. C., Yang, H., & Oh, D. H. (2011). Reliability and validity of the Korean version of the childhood trauma questionnaire-short form for psychiatric outpatients. *Psychiatry investigation*, *8*(4), 305.
139. Kimball, J. S. (2003). *Self-mutilation as an affect regulation strategy: The role of attachment and childhood sexual abuse*. Seattle Pacific University.
140. King, A. (2014). Violent Experiences Questionnaire predictors of low base-rate aggressive acts. *Journal of Aggression, Maltreatment & Trauma*, *23*(8), 804-822.
141. King, A. R., & Russell, T. D. (2017). Psychometric properties of the violent experiences questionnaire. *Child abuse & neglect*, *67*, 64-75.
142. Kong, S., & Bernstein, K. (2009). Childhood trauma as a predictor of eating psychopathology and its mediating variables in patients with eating disorders. *Journal of Clinical Nursing*, *18*(13), 1897-1907.
143. Kristjansson, S., McCutcheon, V. V., Agrawal, A., Lynskey, M. T., Conroy, E., Statham, D. J., ... & Nelson, E. C. (2016). The variance shared across forms of childhood trauma is strongly associated with liability for psychiatric and substance use disorders. *Brain and behavior*, *6*(2), e00432.
144. Kroll, J., Fiszdon, J., & Crosby, R. D. (1996). Childhood abuse and three measures of altered states of consciousness (dissociation, absorption and mysticism) in a female outpatient sample. *Journal of Personality Disorders*, *10*(4), 345-354.
145. Kuhlman, K. R., Maercker, A., Bachem, R., Simmen, K., & Burri, A. (2013). Developmental and contextual factors in the role of severe childhood trauma in geriatric depression: The sample case of former indentured child laborers. *Child Abuse & Neglect*, *37*(11), 969-978.
146. Kuo, J. R., Khoury, J. E., Metcalfe, R., Fitzpatrick, S., & Goodwill, A. (2015). An examination of the relationship between childhood emotional abuse and borderline personality disorder features: The role of difficulties with emotion regulation. *Child abuse & neglect*, *39*, 147-155.
147. Lambert, D. K. (2010). Violence, Depression, Parental Stress, and Child Neglect among High Risk Postpartum Women.
148. Lamela, D., & Figueiredo, B. (2013). Parents' physical victimization in childhood and current risk of child maltreatment: The mediator role of psychosomatic symptoms. *Journal of psychosomatic research*, *75*(2), 178-183.
149. Lara, M. A., Navarrete, L., Nieto, L., & Le, H. N. (2015). Childhood abuse increases the risk of depressive and anxiety symptoms and history of suicidal behavior in Mexican pregnant women. *Brazilian Journal of Psychiatry*, *37*, 203-210.
150. Leary, C. E., Kelley, M. L., Morrow, J., & Mikulka, P. J. (2008). Parental use of physical punishment as related to family environment, psychological well-being, and personality in undergraduates. *Journal of family violence*, *23*(1), 1-7.
151. Lee, Y., & Kim, S. (2011). Childhood maltreatment in South Korea: retrospective study. *Child Abuse & Neglect*, *35*(12), 1037-1044.
152. Lev-Wiesel, R., & Daphna-Tekoa, S. (2007). Prenatal posttraumatic stress symptomatology in pregnant survivors of childhood sexual abuse: A brief report. *Journal of Loss and Trauma*, *12*(2), 145-153.
153. Lev-Wiesel, R., & Markus, L. (2013). Perception vs. circumstances of the child sexual abuse event in relation to depression and post-traumatic stress symptomatology. *Journal of child sexual abuse*, *22*(5), 519-533.
154. Long, P. J. (2002). Psychometric validation of the Life Experiences Questionnaire (LEQ). *Unpublished manuscript, Oklahoma State University*.
155. Levenson, J. S., & Grady, M. D. (2016). The influence of childhood trauma on sexual violence and sexual deviance in adulthood. *Traumatology*, *22*(2), 94.
156. Li, Y., Long, Z., Cao, D., & Cao, F. (2017). Maternal history of child maltreatment and maternal depression risk in the perinatal period: a longitudinal study. *Child abuse & neglect*, *63*, 192-201.
157. Lipschitz, D. S., Kaplan, M. L., Sorkenn, J., Chorney, P., & Asnis, G. M. (1996). Childhood abuse, adult assault, and dissociation. *Comprehensive Psychiatry*, *37*(4), 261-266.
158. Litty, C. G., Kowalski, R., & Minor, S. (1996). Moderating effects of physical abuse and perceived social support on the potential to abuse. *Child Abuse & Neglect*, *20*(4), 305-314.
159. Lobbestael, J., Arntz, A., Harkema-Schouten, P., & Bernstein, D. (2009). Development and psychometric evaluation of a new assessment method for childhood maltreatment experiences: The interview for traumatic events in childhood (ITEC). *Child Abuse & Neglect*, *33*(8), 505-517.
160. Lock, T. G., Levis, D. J., & Rourke, P. A. (2005). The sexual abuse questionnaire: a preliminary examination of a time and cost efficient method in evaluating the presence of childhood sexual abuse in adult patients. *Journal of child sexual abuse*, *14*(1), 1-26.
161. Lopez-Stane, M. A. (2006). *Understanding childhood psychological maltreatment in an urban university population*. The University of Wisconsin-Milwaukee.
162. Lotzin, A., Haupt, L., von Schönfels, J., Wingenfeld, K., & Schäfer, I. (2016). profiles of childhood trauma in patients with alcohol dependence and their associations with addiction‐related problems. *Alcoholism: Clinical and Experimental Research*, *40*(3), 543-552.
163. Love, A. (2011). A Quantitative examination of the impact of acculturation and childhood trauma on resilience and health status among native Hawaiians. Saybrook University.
164. Melchert, T. P., & Sayger, T. V. (1998). The development of an instrument for measuring memories of family of origin characteristics. *Educational and Psychological Measurement*, *58*(1), 99-118.
165. Messman-Moore, T. L., & Long, P. J. (2000). Child sexual abuse and revictimization in the form of adult sexual abuse, adult physical abuse, and adult psychological maltreatment. *Journal of interpersonal violence*, *15*(5), 489-502.
166. Machisa, M. T., Christofides, N., & Jewkes, R. (2017). Mental ill health in structural pathways to women’s experiences of intimate partner violence. *PloS one*, *12*(4), e0175240.
167. MacMillan, H. L., Fleming, J. E., Streiner, D. L., Lin, E., Boyle, M. H., Jamieson, E., ... & Beardslee, W. R. (2001). Childhood abuse and lifetime psychopathology in a community sample. *American Journal of Psychiatry*, *158*(11), 1878-1883.
168. Mair, C., Cunradi, C. B., & Todd, M. (2012). Adverse childhood experiences and intimate partner violence: Testing psychosocial mediational pathways among couples. *Annals of epidemiology*, *22*(12), 832-839.
169. Mallow, A. J. (2000). *The severity of childhood sexual abuse and its relationship to the severity of chemical dependency in adults*. Adelphi University, School of Social Work.
170. Mancini, C., Van Ameringen, M., & MacMillan, H. (1995). Relationship of childhood sexual and physical abuse to anxiety disorders. *Journal of Nervous and Mental Disease*.
171. Marcy, S. N. (1998). *Childhood maltreatment and negative sequelae in young adults: A comparison between outcomes of psychological, physical, and sexual maltreatment*. Boston University.
172. Marquee-Flentje, S. L. (2015). *Psychological adjustment of adult female survivors of CSA as a function of developmental level, self-directed disclosure and parental support* (Doctoral dissertation, Fielding Graduate University).
173. Marshall, W. L., & Mazzucco, A. (1995). Self-esteem and parental attachments in child molesters. *Sexual Abuse: A Journal of Research and Treatment*, *7*(4), 279-285.
174. McCall-Hosenfeld, J. S., Winter, M., Heeren, T., & Liebschutz, J. M. (2014). The association of interpersonal trauma with somatic symptom severity in a primary care population with chronic pain: exploring the role of gender and the mental health sequelae of trauma. *Journal of psychosomatic research*, *77*(3), 196-204.
175. McCarty, C. S. (1999). *Examining moderator variables' unique impact upon physical child abuse*. University of Arkansas.
176. McGinn A. D. (2015). *Early trauma and subsequent health and risk behaviors.*.University of Arkansas. Indiana University.
177. Melchert, T. P., & Kalemeera, A. (2009). A brief version of the family background questionnaire. *Measurement and Evaluation in Counseling and Development*, *41*(4), 210-222.
178. Mersky, J. P., Janczewski, C. E., & Topitzes, J. (2017). Rethinking the measurement of adversity: Moving toward second-generation research on adverse childhood experiences. *Child maltreatment*, *22*(1), 58-68.
179. Merza, K., Papp, G., & Kuritárné Szabó, I. (2015). The role of childhood traumatization in the development of borderline personality disorder in Hungary. *The European Journal of Psychiatry*, *29*(2), 105-118.
180. Merrill, L. L. (2001). Trauma symptomatology among female US Navy recruits. *Military medicine*, *166*(7), 621-624.
181. Messman-Moore, T. L., & Brown, A. L. (2004). Child maltreatment and perceived family environment as risk factors for adult rape: is child sexual abuse the most salient experience?. *Child Abuse & Neglect*, *28*(10), 1019-1034.
182. El Mhamdi, S., Lemieux, A., Bouanene, I., Salah, A. B., Nakajima, M., Salem, K. B., & Al'absi, M. (2017). Gender differences in adverse childhood experiences, collective violence, and the risk for addictive behaviors among university students in Tunisia. *Preventive medicine*, *99*, 99-104.
183. Miller-Perrin, C. L., Perrin, R. D., & Kocur, J. L. (2009). Parental physical and psychological aggression: Psychological symptoms in young adults. *Child abuse & neglect*, *33*(1), 1-11.
184. Milletich, R. J., Kelley, M. L., Doane, A. N., & Pearson, M. R. (2010). Exposure to interparental violence and childhood physical and emotional abuse as related to physical aggression in undergraduate dating relationships. *Journal of family violence*, *25*(7), 627-637.
185. Mondragon, E. A. (2005). *The effects of family related factors on the development of serious violence adolescent behaviour.* University of La Verne.
186. Nuckols, A. E. (2010). *The roles of paternal attachment and psychopathy in the cycle of sexual abuse: Examining sex offenders and non-sex offenders*. Alliant International University, Fresno.
187. Montgomery, A. E., Cutuli, J. J., Evans-Chase, M., Treglia, D., & Culhane, D. P. (2013). Relationship among adverse childhood experiences, history of active military service, and adult outcomes: Homelessness, mental health, and physical health. *American journal of public health*, *103*(S2), S262-S268.
188. Nakai, Y., Inoue, T., Toda, H., Toyomaki, A., Nakato, Y., Nakagawa, S., ... & Kusumi, I. (2014). The influence of childhood abuse, adult stressful life events and temperaments on depressive symptoms in the nonclinical general adult population. *Journal of affective disorders*, *158*, 101-107.
189. Naqavi, M. R., Mohammadi, M., Salari, V., & Nakhaee, N. (2011). The relationship between childhood maltreatment and opiate dependency in adolescence and middle age. *Addiction & health*, *3*(3-4), 92.
190. Narayan, J. (2009). *Emotional intelligence as a protective factor against traumatic stress in young adults*. University of Hartford.
191. Nash, C. L., Hayes-Skelton, S. A., & DiLillo, D. (2012). Reliability and factor structure of the psychological maltreatment and neglect scales of the Computer Assisted Maltreatment Inventory (CAMI). *Journal of Aggression, Maltreatment & Trauma*, *21*(5), 583-607.
192. Nicholas, K. B., & Bieber, S. L. (1997). Assessment of perceived parenting behaviors: The exposure to abusive and supportive environments parenting inventory (EASE-PI). *Journal of Family Violence*, *12*(3), 275-291.
193. Nickel, M. K., Tritt, K., Mitterlehner, F. O., Leiberich, P., Nickel, C., Lahmann, C., ... & Loew, T. H. (2004). Sexual abuse in childhood and youth as psychopathologically relevant life occurrence: cross-sectional survey. *Croatian medical journal*, *45*(4), 483-489.
194. Osório, F. L., Salum, G. A., Donadon, M. F., Forni-dos-Santos, L., Loureiro, S. R., & Crippa, J. A. S. (2013). Psychometrics properties of early trauma inventory self report–short form (ETISR-SR) for the Brazilian context. *PLoS One*, *8*(10), e76337.
195. Park, K., Shim, G., & Jeong, B. (2020). Validation of the Traumatic Antecedents Questionnaire using item response theory. *Brain and behavior*, *10*(12), e01870.
196. Ono, K., Takaesu, Y., Nakai, Y., Shimura, A., Ono, Y., Murakoshi, A., ... & Inoue, T. (2017). Associations among depressive symptoms, childhood abuse, neuroticism, and adult stressful life events in the general adult population. *Neuropsychiatric disease and treatment*, *13*, 477.
197. Paivio, S. C. (2001). Stability of retrospective self-reports of child abuse and neglect before and after therapy for child abuse issues☆. *Child abuse & neglect*, *25*(8), 1053-1068.
198. Paivio, S. C., & Cramer, K. M. (2004). Factor structure and reliability of the Childhood Trauma Questionnaire in a Canadian undergraduate student sample. *Child abuse & neglect*, *28*(8), 889-904.
199. Park, S., Nam, Y. Y., Sim, Y., & Hong, J. P. (2015). Interactions between the apolipoprotein E ɛ 4 allele status and adverse childhood experiences on depressive symptoms in older adults. *European journal of psychotraumatology*, *6*(1), 25178.
200. Patterson, M. L., Moniruzzaman, A., & Somers, J. M. (2014). Setting the stage for chronic health problems: cumulative childhood adversity among homeless adults with mental illness in Vancouver, British Columbia. *BMC public health*, *14*(1), 1-10.
201. Pereira da Silva, S. S., & da Costa Maia, Â. (2013). The stability of self-reported adverse experiences in childhood: a longitudinal study on obesity. *Journal of interpersonal violence*, *28*(10), 1989-2004.
202. Pereda, N., & Gallardo-Pujol, D. (2014). One hit makes the difference: The role of polyvictimization in childhood in lifetime revictimization on a southern European sample. *Violence and Victims*, *29*(2), 217-231.
203. Pflugradt, D. M., Allen, B. P., & Zintsmaster, A. J. (2018). Adverse childhood experiences of violent female offenders: A comparison of homicide and sexual perpetrators. *International journal of offender therapy and comparative criminology*, *62*(8), 2312-2328.
204. Pitzner, J. K., & Drummond, P. D. (1997). The reliability and validity of empirically scaled measures of psychological/verbal control and physical/sexual abuse: relationship between current negative mood and a history of abuse independent of other negative life events. *Journal of psychosomatic research*, *43*(2), 125-142.
205. Plaza, A., Torres, A., Martin-Santos, R., Gelabert, E., Imaz, M. L., Navarro, P., ... & Garcia-Esteve, L. (2011). Validation and test-retest reliability of early trauma inventory in Spanish postpartum women. *The Journal of nervous and mental disease*, *199*(4), 280.
206. Pompili, M., Iliceto, P., Innamorati, M., Rihmer, Z., Lester, D., Akiskal, H. S., ... & Tatarelli, R. (2009). Suicide risk and personality traits in physically and/or sexually abused acute psychiatric inpatients: a preliminary study. *Psychological reports*, *105*(2), 554-568.
207. Pompili, M., Innamorati, M., Lamis, D. A., Erbuto, D., Venturini, P., Ricci, F., ... & Girardi, P. (2014). The associations among childhood maltreatment,“male depression” and suicide risk in psychiatric patients. *Psychiatry research*, *220*(1-2), 571-578.
208. Poole, J. C., Dobson, K. S., & Pusch, D. (2017). Childhood adversity and adult depression: the protective role of psychological resilience. *Child abuse & neglect*, *64*, 89-100.
209. Powers, A. D., Thomas, K. M., Ressler, K. J., & Bradley, B. (2011). The differential effects of child abuse and posttraumatic stress disorder on schizotypal personality disorder. *Comprehensive psychiatry*, *52*(4), 438-445.
210. Quinn, M., Caldara, G., Collins, K., Owens, H., Ozodiegwu, I., Loudermilk, E., & Stinson, J. D. (2018). Methods for understanding childhood trauma: modifying the adverse childhood experiences international questionnaire for cultural competency. *International journal of public health*, *63*(1), 149-151.
211. Ryan, S. H. W. (1993). *Psychometric analysis of the sexual abuse exposure questionnaire* (Doctoral dissertation, Fuller Theological Seminary, School of Psychology).
212. Rajkumar, R. P. (2015). The impact of childhood adversity on the clinical features of schizophrenia. *Schizophrenia research and treatment*, *2015*.
213. Ramiro, L. S., Madrid, B. J., & Brown, D. W. (2010). Adverse childhood experiences (ACE) and health-risk behaviors among adults in a developing country setting. *Child abuse & neglect*, *34*(11), 842-855.
214. Rankin, M. E. (1999). *Construct validation of the Child Abuse and Trauma Scale: Comparison to data obtained from a structured interview*. University of Connecticut.
215. Rausch, M. A. (2016). Adverse childhood experiences and intimate partner violence in lesbian and queer relationships. *Journal of LGBT issues in counseling*, *10*(2), 97-111.
216. Rieder, H., & Elbert, T. (2013). The relationship between organized violence, family violence and mental health: findings from a community-based survey in Muhanga, Southern Rwanda. *European Journal of Psychotraumatology*, *4*(1), 21329.
217. Reuben, A., Moffitt, T. E., Caspi, A., Belsky, D. W., Harrington, H., Schroeder, F., ... & Danese, A. (2016). Lest we forget: comparing retrospective and prospective assessments of adverse childhood experiences in the prediction of adult health. *Journal of Child Psychology and Psychiatry*, *57*(10), 1103-1112.
218. Riddle, K. P., & Aponte, J. F. (1999). The comprehensive childhood maltreatment inventory: Early development and reliability analyses. *Child Abuse & Neglect*, *23*(11), 1103-1115.
219. Riddle, K. P. (1997). *The development of a self-report research instrument for the comprehensive assessment of child maltreatment based on retrospective memory*. University of Louisville.
220. Roh, S., Burnette, C. E., Lee, K. H., Lee, Y. S., Easton, S. D., & Lawler, M. J. (2015). Risk and protective factors for depressive symptoms among American Indian older adults: Adverse childhood experiences and social support. *Aging & mental health*, *19*(4), 371-380.
221. Rohlehr, L. N. (2014). *The moderating effects of resilience in the relationship between childhood physical abuse, criminal behavior, and antisocial traits* (Doctoral dissertation, Fordham University).
222. Rosen, L. N., & Martin, L. (1996). Impact of childhood abuse history on psychological symptoms among male and female soldiers in the US Army. *Child Abuse & Neglect*, *20*(12), 1149-1160.
223. Rosen, L. N., & Martin, L. (1998). Long-term effects of childhood maltreatment history on gender-related personality characteristics. *Child Abuse & Neglect*, *22*(3), 197-211.
224. Simonelli, A., Sacchi, C., Cantoni, L., Brown, M., & Frewen, P. (2017). Italian translation and cross-cultural comparison with the Childhood Attachment and Relational Trauma Screen (CARTS). *European journal of psychotraumatology*, *8*(1), 1375839.
225. Saraçlı, Ö., Atasoy, N., Şenormancı, Ö., Atik, L., Açıkgöz, H. O., Doğan, V., ... & Örsel, S. (2016). Childhood trauma and suicide risk in the population living in Z onguldak P rovince. *Asia‐Pacific Psychiatry*, *8*(2), 136-144.
226. Sarchiapone, M., Jaussent, I., Roy, A., Carli, V., Guillaume, S., Jollant, F., ... & Courtet, P. (2009). Childhood trauma as a correlative factor of suicidal behavior–via aggression traits. Similar results in an Italian and in a French sample. *European Psychiatry*, *24*(1), 57-62.
227. Sacchi, C., Vieno, A., & Simonelli, A. (2018). Italian validation of the Childhood Trauma Questionnaire—Short Form on a college group. *Psychological trauma: theory, research, practice, and policy*, *10*(5), 563.
228. Sacco, K. A., George, T. P., Head, C. A., Vessicchio, J. C., Easton, C. J., & Prigerson, H. G. (2007). Adverse childhood experiences, smoking and mental illness in adulthood: a preliminary study. *Annals of Clinical Psychiatry*, *19*(2), 89-97.
229. Sanders, B., & Becker-Lausen, E. (1995). The measurement of psychological maltreatment: Early data on the child abuse and trauma scale. *Child abuse & neglect*, *19*(3), 315-323.
230. Schalinski, I., Teicher, M. H., Nischk, D., Hinderer, E., Müller, O., & Rockstroh, B. (2016). Type and timing of adverse childhood experiences differentially affect severity of PTSD, dissociative and depressive symptoms in adult inpatients. *BMC psychiatry*, *16*(1), 1-15.
231. Scher, C. D., Stein, M. B., Ingram, R. E., Malcarne, V. L., & McQuaid, J. R. (2002). The Parent Threat Inventory: development, reliability, and validity. *Child abuse & neglect*, *26*(2), 207-225.
232. Schilling, C., Weidner, K., Brähler, E., Glaesmer, H., Häuser, W., & Pöhlmann, K. (2016). Patterns of childhood abuse and neglect in a representative German population sample. *PloS one*, *11*(7), e0159510.
233. Schilling, E. A., Aseltine, R. H., & Gore, S. (2007). Adverse childhood experiences and mental health in young adults: a longitudinal survey. *BMC public health*, *7*(1), 1-10.
234. Schmidt, M. R., Narayan, A. J., Atzl, V. M., Rivera, L. M., & Lieberman, A. F. (2020). Childhood maltreatment on the Adverse Childhood Experiences (ACEs) Scale versus the Childhood Trauma Questionnaire (CTQ) in a perinatal sample. *Journal of Aggression, Maltreatment & Trauma*, *29*(1), 38-56.
235. Schulz, A., Becker, M., Van der Auwera, S., Barnow, S., Appel, K., Mahler, J., ... & Grabe, H. J. (2014). The impact of childhood trauma on depression: Does resilience matter? Population-based results from the Study of Health in Pomerania. *Journal of psychosomatic research*, *77*(2), 97-103.
236. Scioli-Salter, E. R., Johnides, B. D., Mitchell, K. S., Smith, B. N., Resick, P. A., & Rasmusson, A. M. (2016). Depression and dissociation as predictors of physical health symptoms among female rape survivors with posttraumatic stress disorder. *Psychological trauma: theory, research, practice, and policy*, *8*(5), 585.
237. Şenkal, İpek, and Sedat IŞIKLI. "Childhood Traumas and Attachment Style-Associated Depression Symptoms: The Mediator Role of Alexithymia." *Turk Psikiyatri Dergisi* 26.4 (2015).
238. Sfoggia, A., Pacheco, M. A., & Grassi-Oliveira, R. (2008). History of childhood abuse and neglect and suicidal behavior at hospital admission. *Crisis*, *29*(3), 154-158.
239. Shchupak, T. (2015). Convergent validity of two measures of childhood sexual abuse: Russell Sexual Abuse Interview Schedule and the Finkelhor Survey of Childhood Experiences.
240. Simon, N. M., Herlands, N. N., Marks, E. H., Mancini, C., Letamendi, A., Li, Z., ... & Stein, M. B. (2009). Childhood maltreatment linked to greater symptom severity and poorer quality of life and function in social anxiety disorder. *Depression and anxiety*, *26*(11), 1027-1032.
241. Simonelli, C. J., Mullis, T., & Rohde, C. (2005). Scale of negative family interactions: A measure of parental and sibling aggression. *Journal of Interpersonal Violence*, *20*(7), 792-803.
242. Sims, E. N., Dodd, V. J. N., & Tejeda, M. J. (2008). The relationship between severity of violence in the home and dating violence. *Journal of Forensic Nursing*, *4*(4), 166-173.
243. Singh, S., Manjula, M., & Philip, M. (2012). Suicidal risk and childhood adversity: A study of Indian college students. *Asian journal of psychiatry*, *5*(2), 154-159.
244. Smith, N., Lam, D., Bifulco, A., & Checkley, S. (2002). Childhood experience of care and abuse questionnaire (CECA. Q). *Social Psychiatry and Psychiatric Epidemiology*, *37*(12), 572-579.
245. Spinhoven, P., Penninx, B. W., Hickendorff, M., van Hemert, A. M., Bernstein, D. P., & Elzinga, B. M. (2014). Childhood Trauma Questionnaire: factor structure, measurement invariance, and validity across emotional disorders. *Psychological assessment*, *26*(3), 717.
246. Spitzer, C., Bouchain, M., Winkler, L. Y., Wingenfeld, K., Gold, S. M., Grabe, H. J., ... & Heesen, C. (2012). Childhood trauma in multiple sclerosis: a case-control study. *Psychosomatic medicine*, *74*(3), 312-318.
247. Spitzer, C., Chevalier, C., Gillner, M., Freyberger, H. J., & Barnow, S. (2006). Complex posttraumatic stress disorder and child maltreatment in forensic inpatients. *The Journal of Forensic Psychiatry & Psychology*, *17*(2), 204-216.
248. Steel, J., Sanna, L., Hammond, B., Whipple, J., & Cross, H. (2004). Psychological sequelae of childhood sexual abuse: Abuse-related characteristics, coping strategies, and attributional style. *Child abuse & neglect*, *28*(7), 785-801.
249. Stoltz, J. A. M., Shannon, K., Kerr, T., Zhang, R., Montaner, J. S., & Wood, E. (2007). Associations between childhood maltreatment and sex work in a cohort of drug-using youth. *Social science & medicine*, *65*(6), 1214-1221.
250. Straus, M. A. (2006). Cross-cultural reliability and validity of the multidimensional neglectful behavior scale adult recall short form. *Child Abuse & Neglect*, *30*(11), 1257-1279.
251. Strine, T. W. (2010). *The mediating role of psychological distress in the relationship between adverse childhood experiences and adult smoking* (Doctoral dissertation, Walden University).
252. Swahnberg, I. K., & Wijma, B. (2003). The NorVold Abuse Questionnaire (NorAQ) Validation of new measures of emotional, physical, and sexual abuse, and abuse in the health care system among women. *The European Journal of Public Health*, *13*(4), 361-366.
253. Swahnberg, K. (2011). NorVold Abuse Questionnaire for men (m-NorAQ): validation of new measures of emotional, physical, and sexual abuse and abuse in health care in male patients. *Gender medicine*, *8*(2), 69-79.
254. Swift, E. E., & Gayton, W. F. (1996). Further validation of the psychological maltreatment inventory. *Journal of clinical psychology*, *52*(3), 325-327.
255. Swopes, R. M., Simonet, D. V., Jaffe, A. E., Tett, R. P., & Davis, J. L. (2013). Adverse childhood experiences, posttraumatic stress disorder symptoms, and emotional intelligence in partner aggression. *Violence and Victims*, *28*(3), 513-530.
256. Tanaka, M., Wekerle, C., Leung, E., Waechter, R., Gonzalez, A., Jamieson, E., & MacMillan, H. L. (2012). Preliminary evaluation of the Childhood Experiences of Violence Questionnaire short form. *Journal of Interpersonal Violence*, *27*(2), 396-407.
257. Tardif-Williams, C. Y., Tanaka, M., Boyle, M. H., & MacMillan, H. L. (2017). The impact of childhood abuse and current mental health on young adult intimate relationship functioning. *Journal of interpersonal violence*, *32*(22), 3420-3447.
258. Teicher, M. H., & Parigger, A. (2015). The ‘Maltreatment and Abuse Chronology of Exposure’(MACE) scale for the retrospective assessment of abuse and neglect during development. *PLoS one*, *10*(2), e0117423.
259. Tresno, F., Ito, Y., & Mearns, J. (2013). Risk factors for nonsuicidal self-injury in Japanese college students: The moderating role of mood regulation expectancies. *International Journal of Psychology*, *48*(6), 1009-1017.
260. Thabet, A. A. M., Tischler, V., & Vostanis, P. (2004). Maltreatment and coping strategies among male adolescents living in the Gaza Strip. *Child abuse & neglect*, *28*(1), 77-91.
261. Thombs, B. D., Bernstein, D. P., Lobbestael, J., & Arntz, A. (2009). A validation study of the Dutch Childhood Trauma Questionnaire-Short Form: factor structure, reliability, and known-groups validity. *Child abuse & neglect*.
262. Thombs, B. D., Lewis, C., Bernstein, D. P., Medrano, M. A., & Hatch, J. P. (2007). An evaluation of the measurement equivalence of the Childhood Trauma Questionnaire—Short Form across gender and race in a sample of drug-abusing adults. *Journal of psychosomatic research*, *63*(4), 391-398.
263. Thomson, P., & Jaque, S. (2015). Posttraumatic stress disorder and psychopathology in dancers. *Medical problems of performing artists*, *30*(3), 157-162.
264. Toda, H., Inoue, T., Tsunoda, T., Nakai, Y., Tanichi, M., Tanaka, T., ... & Kusumi, I. (2016). Affective temperaments play an important role in the relationship between childhood abuse and depressive symptoms in major depressive disorder. *Psychiatry research*, *236*, 142-147.
265. Tousignant, M., Pouliot, L., Routhier, D., Vrakas, G., McGirr, A., & Turecki, G. (2011). Suicide, schizophrenia, and schizoid‐type psychosis: Role of life events and childhood factors. *Suicide and Life‐Threatening Behavior*, *41*(1), 66-78.
266. Tran, Q. A., Dunne, M. P., Vo, T. V., & Luu, N. H. (2015). Adverse childhood experiences and the health of university students in eight provinces of Vietnam. *Asia Pacific Journal of Public Health*, *27*(8_suppl), 26S-32S.
267. Twomey, H. B., Kaslow, N. J., & Croft, S. (2000). Childhood maltreatment, object relations, and suicidal behavior in women. *Psychoanalytic Psychology*, *17*(2), 313.
268. VanDeusen, K. M., & Way, I. (2006). Vicarious trauma: An exploratory study of the impact of providing sexual abuse treatment on clinicians' trust and intimacy. *Journal of child sexual abuse*, *15*(1), 69-85.
269. Van Leeuwen, K. G., Fauchier, A., & Straus, M. A. (2012). Assessing dimensions of parental discipline. *Journal of psychopathology and behavioral assessment*, *34*(2), 216-231.
270. Verona, E., Murphy, B., & Javdani, S. (2016). Gendered pathways: Violent childhood maltreatment, sex exchange, and drug use. *Psychology of violence*, *6*(1), 124.
271. Villano, C. L., Cleland, C., Rosenblum, A., Fong, C., Nuttbrock, L., Marthol, M., & Wallace, J. (2004). Psychometric utility of the childhood trauma questionnaire with female street-based sex workers. *Journal of trauma & dissociation*, *5*(3), 33-41.
272. Villarroel, A. M., Penelo, E., Portell, M., & Raich, R. M. (2012). Childhood sexual and physical abuse in Spanish female undergraduates: Does it affect eating disturbances?. *European Eating Disorders Review*, *20*(1), e32-e41.
273. Virkler, P. M. (2005). *The relationship between childhood sexual abuse and measures of depression, anxiety and revictimization in females aged 55 to 85*. The University of North Carolina at Charlotte.
274. Voorthuis, A., Bhandari, R., Out, D., van der Veen, R., Bakermans-Kranenburg, M. J., & Van IJzendoorn, M. H. (2014). Childhood maltreatment experiences and child abuse potential: Temperamental sensitivity as moderator?. *Journal of family Violence*, *29*(7), 749-756.
275. Wade Jr, R., Becker, B. D., Bevans, K. B., Ford, D. C., & Forrest, C. B. (2017). Development and evaluation of a short adverse childhood experiences measure. *American journal of preventive medicine*, *52*(2), 163-172.
276. Walsh, E. G., & Cawthon, S. W. (2014). The mediating role of depressive symptoms in the relationship between adverse childhood experiences and smoking. *Addictive Behaviors*, *39*(10), 1471-1476.
277. Weibel, S., Vidal, S., Olié, E., Hasler, R., Torriani, C., Prada, P., ... & Huguelet, P. (2017). Impact of child maltreatment on meaning in life in psychiatric patients. *Psychiatry research*, *251*, 204-211.
278. Welles, S. L., Patel, F., & Chilton, M. (2017). Does employment-related resilience affect the relationship between childhood adversity, community violence, and depression?. *Journal of urban health*, *94*(2), 233-243.
279. Whitfield, C. L., Anda, R. F., Dube, S. R., & Felitti, V. J. (2003). Violent childhood experiences and the risk of intimate partner violence in adults: Assessment in a large health maintenance organization. *Journal of interpersonal violence*, *18*(2), 166-185.
280. Wildes, J. E., Kalarchian, M. A., Marcus, M. D., Levine, M. D., & Courcoulas, A. P. (2008). Childhood maltreatment and psychiatric morbidity in bariatric surgery candidates. *Obesity surgery*, *18*(3), 306-313.
281. Williams, T. L. (2001). *The development and validation of a multi-dimensional assessment instrument of child sexual abuse experiences*. Texas A&M University.
282. Wolfner, G. D. (1996). *Family functioning and physical child abuse: Are certain family types more prone to abuse?*. University of Rhode Island.
283. Wimberly, A. I. W. (2004). *The influence of childhood sexual abuse and depression on substance abusing women*. Howard University.
284. Wright, K. D., Asmundson, G. J., McCreary, D. R., Scher, C., Hami, S., & Stein, M. B. (2001). Factorial validity of the Childhood Trauma Questionnaire in men and women. *Depression and anxiety*, *13*(4), 179-183.
285. Yampolsky, L., Lev‐Wiesel, R., & Ben‐Zion, I. Z. (2010). Child sexual abuse: is it a risk factor for pregnancy?. *Journal of Advanced Nursing*, *66*(9), 2025-2037.
286. Youssef, N. A., Belew, D., Hao, G., Wang, X., Treiber, F. A., Stefanek, M., ... & Su, S. (2017). Racial/ethnic differences in the association of childhood adversities with depression and the role of resilience. *Journal of affective disorders*, *208*, 577-581.
287. Yuan, N. P., Koss, M. P., Polacca, M., & Goldman, D. (2006). Risk factors for physical assault and rape among six Native American tribes. *Journal of Interpersonal Violence*, *21*(12), 1566-1590.
288. Zlotnick, C., Shea, M. T., Pearlstein, T., Simpson, E., Costello, E., & Begin, A. (1996). The relationship between dissociative symptoms, alexithymia, impulsivity, sexual abuse, and self-mutilation. *Comprehensive psychiatry*, *37*(1), 12-16.
