# Supplementary material for: Measuring Violence Against Children: A COSMIN Systematic Review of the Psychometric and Administrative Properties of Adult Retrospective Self-report Instruments on Child Abuse and Neglect
Source: Trauma Violence Abuse. 2023 Jan 25;25(1):183–96. doi: 10.1177/15248380221145912 (PMC10666516; doi:10.1177/15248380221145912)
Supplement: sj-docx-2-tva-10.1177_15248380221145912 – Supplemental material for Measuring Violence Against Children: A COSMIN Systematic Review of the Psychometric and Administrative Properties of Adult Retrospective Self-report Instruments on Child Abuse and Neglect [file sj-docx-2-tva-10.1177_15248380221145912.docx]

**Table 1: Overview of measurement properties and practicalities**

| Instrument | Adaptations | Retrievable | Constructs | Subscales | Recall period | Items | Response Options | Locations | Perpetrators | Disclosure | Frequency | Severity | Trivialisation | Flesch reading score | Mode of application | Time to complete | Languages | User guide | Participant burden | Requirement for use |
| --- | --- | --- | --- | --- | --- | --- | --- | --- | --- | --- | --- | --- | --- | --- | --- | --- | --- | --- | --- | --- |
| **Multiple forms of abuse** | | | | | | | | | | | | | | | | | | | | |
| **Adverse Childhood Experiences (ACE)** |  | Yes | Child abuse | Emotional, physical, and sexual abuse; emotional and physical neglect | Lifetime | 14 | 5 point Likert scale | No | Yes | No | Yes | Yes | No | 77.6 | Tablets or pen and paper | NR | English | Yes | No | NR |
|  | **ACE with spanking (ACE-S)** | Yes | Child abuse | Emotional, physical, and sexual abuse; emotional and physical neglect | Lifetime | 14 | 5 point Likert scale | No | Yes | No | Yes | Yes | No | 77.6 | Tablets or pen and paper | NR | English | Yes | No | NR |
|  | **ACE Abuse Short Form (ACE-ASF)** | Yes | Child abuse | Physical, emotional, and sexual abuse | Before age 15 | 11 | 5 point Likert scale | No | Yes | No | Yes | No | No | 77.6 | Self-completion paper-pencil | 40 minutes | English | Yes | No | NR |
|  | **ACE International Questionnaires (ACE-IQ)** | Yes | Trauma | Emotional, physical, and sexual abuse; emotional and physical neglect | Lifetime | 14 | 5 point Likert scale | No | Yes | No | Yes | Yes | No | 77.6 | Tablets or pen and paper | NR | English | Yes | No | NR |
|  | **ACE-BRFSS (ACE-BRFSS)** | Yes | Adverse childhood experiences | Psychological abuse; physical abuse; sexual abuse | Childhood | 11 | Never, once, more than once | No | Yes | No | Yes | No | No | 83.4 | Self-completion | 6 minutes | English | Yes | No | NR |
| **Aversive und Protektive Kindsheitserfahrungen (APK)** |  | Yes | Adverse childhood experiences | Emotional abuse and neglect; physical abuse and neglect; sexual abuse; other traumatic experiences | Childhood | 19 | Never to very often | No | No | No | Yes | Yes | Yes | 50.4 | Self-completion paper-pencil | NR | German | NR | No | NR |
| **Caregiver History of Loss and Victimization (VICA)** |  | Yes | Child abuse | Physical abuse; excessive punishment; sexual abuse | Childhood | 8 | Yes/No | No | Yes | No | No | Yes | Yes | 65.9 | Self-completion | 5 minutes | English | Yes | No | NR |
| **Childhood Experiences of Violence (CEVQ)** |  | Yes | Childhood violence exposure | Bullying; witnessing domestic violence; emotional abuse; corporal punishment; physical abuse; sexual abuse | Lifetime | 16 screeners | Never to more than 10 times | No | Yes | Yes | Yes | Yes | No | 72.9 | Self-completion | NR | English | NR | Yes | NR |
|  | **CEVQ - Short Form (CEVQ-SF)** | Yes | Childhood violence exposure | Bullying; physical abuse; sexual abuse; physical punishment | Before age 16 | 7 screeners | Never to more than 10 times | No | No | No | Yes | No | No | 72.9 | Self-completion | NR | English | NR | No | NR |
| **Child Abuse and Trauma Scale (CATS)** |  | Yes | Trauma | Neglect; sexual abuse; punishment | Childhood | 38 | Never to always | No | No | No | Yes | No | No | 60.5 | Self-completion | NR | English; Japanese | NR | No | NR |
| **Child Abuse Experiences Inventory (CAEI)** |  | No | Child abuse | Physical abuse; emotional abuse; neglect; exposure to violence between parents | Childhood | 19 | Never to always | No | Yes | No | Yes | No | No | NR | Self-completion | NR | Croatian | NR | No | NR |
| **Child Attachment and Relational Trauma Screen (CARTS)** |  | Yes | Child maltreatment | Emotional abuse; sexual abuse; physical abuse | Lifetime | 69 (13 on child abuse) | NR | No | No | No | No | Yes | No | 74.9 | Computer based self-report measure | 15 minutes | Italian; English | Yes | No | NR |
| **Child Maltreatment History Self-Report (CMH-SR)** |  | Yes | Child maltreatment | Physical abuse; sexual abuse | Childhood | 11 | Never to often for physical abuse; Yes/No for sexual abuse | No | No | No | Yes | No | No | 78.5 | Self-completion | 5 minutes | English | NR | No | NR |
| **Child Maltreatment Interview Schedule-Short Form (CMIS-SF)** |  | Yes | Child abuse | Witnessing intimate partner violence; psychological unavailability of parents; psychological abuse; physical abuse; sexual abuse | Childhood | 10 | Yes/No | No | Yes | No | Yes | Yes | No | 65.7 | Self-completion | NR | English | NR | No | NR |
| **Child Maltreatment Questionnaire (CMQ)** |  | No | Child maltreatment | Psychological abuse; physical abuse; parental and non-parental sexual abuse | Before age 18 | 132 | Never to very often | NR | NR | NR | Yes | No | NR | NR | Self-report | NR | English | NR | NR | NR |
| **Childhood Experiences of Care and Abuse (CECA)** |  | Yes | Child maltreatment | Parental care (neglect and apathy); physical abuse; sexual abuse | Lifetime | 12 | 5 point Likert scale | No | Yes | No | No | Yes | No | 72.9 | Self-completion paper-pencil questionnaire sent to participants homes | 40-120 minutes | English | Yes | No | NR |
| **Childhood Experiences Survey (CES)** |  | Yes | Adverse childhood experiences | Physical abuse; emotional abuse; sexual abuse; household dysfunction; household adversity | Before age 18 | 17 | Never to very often | No | No | No | Yes | No | NR | 70.1 | Self-completion | NR | English | NR | NR | NR |
| **Childhood History Questionnaire (CHQ1)** |  | No | Violence exposure in childhood | Physical abuse; sexual abuse | Before age 13 | 17 | Never to very often | No | No | No | NR | Yes | NR | 70.3 | Self-completion | NR | English | NR | NR | NR |
| **Childhood History Questionnaire (CHQ2)** |  | No | Adverse childhood experiences | Emotional abuse; physical abuse; physical neglect; emotional neglect; sexual abuse; witnessing domestic violence; household dysfunction | Before age 18 | 24 | Never to very often | No | No | No | Yes | No | NR | 70.3 | Self-completion | NR | Portuguese | NR | NR | NR |
| **Childhood Trauma Interview (CTI)** |  | Yes | Trauma | Separation from caregivers; physical neglect; emotional neglect; physical abuse; emotional abuse; sexual abuse; witnessing violence | Before age 17 | 7 screeners | NR | No | Yes | Yes | Yes | Yes | No | Interview | Interviewer-led | 20-30 minutes | English | NR | No | NR |
| **Childhood Trauma Questionnaire (CTQ)** |  | Yes | Trauma | Sexual abuse; physical abuse; emotional abuse; emotional neglect; physical neglect; denial scale | Lifetime | 70 | Never true to very often true | NR | NR | NR | Yes | Yes | Yes | 66.1 | Face to face, ACASI, Self-completion with paper and pencil | 15-20 minutes | English | Yes | NR | Qualified professional required for administration |
|  | **CTQe Short From (CTQ-SF)** | Yes | Trauma | Physical abuse; physical neglect; emotional abuse; emotional neglect; sexual abuse | Lifetime | 28 | Never true to very often true | No | No | No | No | Yes | Yes | 66.1 | Self-completion | 5 minutes | English | Yes | No | People certified by a professional organisation recognised by Pearson Assessment |
| **Christchurch Trauma Assessment (CTA)** |  | Yes | Trauma | Physical abuse; sexual abuse; exposure to domestic violence | before age 16 | 50 | Varies | Yes | Yes | Yes | Yes | Yes | Yes | 81.9 | Self-completion or computer-assisted interviewing administered via telephone | NR | English | NR | NR | NR |
| **Comprehensive Child Maltreatment Scale (CCMS)** |  | Yes | Child maltreatment | Sexual abuse; physical abuse; psychological maltreatment; neglect; witnessing family violence | Before age 18 | 22 | Never to more than 20 times | No | Yes | No | Yes | No | No | 58.1 | Self-completion paper-pencil | NR | English | NR | No | NR |
| **Comprehensive Childhood Maltreatment Inventory (CCMI)** |  | Yes | Child maltreatment | Physical abuse; emotional abuse; sexual abuse; physical neglect; psychological neglect | Before age 18 | 31 | Yes/No | No | Yes | Yes | Yes | Yes | No | 64.5 | Self-completion | NR | English | NR | No | NR |
| **Computer-Assisted Maltreatment Inventory (CAMI)** |  | Yes | Child maltreatment | Physical abuse; sexual abuse; psychological abuse; neglect; exposure to domestic violence | Before age 18 | NR | Never to more than 10 times | No | Yes | No | No | Yes | NR | 82.8 | Self-completion or computer-based | 7-20 minutes | English | NR | NR | NR |
| **Parent-Child Conflict Tactics Scales (CTS-PC)** |  | Yes | Physical discipline | Psychological aggression; physical assault; severe assault; very severe assault; non-violent discipline | Before age 18 | 44 | Never to more than 20 times | No | Yes | No | Yes | Yes | No | 100 | Self-completion or interviewer led | NR | English | Yes | No | People certified by a professional organisation recognised by Pearson Assessment |
| **Dimensions of Discipline Inventory (DDI)** |  | Yes | Parental discipline | Physical abuse; emotional abuse | At age 10 | 26 | Never to two or more times a day | No | Yes | No | Yes | No | No | 53.4 | Self-completion | NR | English | Yes | No | NR |
| **Early Trauma Inventory (ETI)** |  | Yes | Trauma | Physical abuse; emotional abuse; sexual abuse; general trauma | Before age 19 | 62 | Yes/No | No | Yes | No | No | No | No | 84.8 | Self-completion | 30 minutes | English; Dutch; Korean | Yes | No | NR |
|  | **ETI Short Form (ETI-SF)** | Yes | Trauma | Physical abuse; emotional abuse; sexual abuse | Before age 18 | 29 | Yes/No | No | No | No | No | No | No | 84.8 | Self-completion | 15 minutes | English | Yes | No | NR |
| **Exposure to Abusive and Supportive Environments Parenting Inventory (EASE-PI)** |  | Yes | Parenting | Emotional abuse; sexual abusiveness; physical abusiveness; love and support; promotion of independence; positive modeling | Before age 18 | 70 | Never to very often | No | Yes | No | Yes | No | NR | 84 | Self-completion | NR | English | NR | NR | NR |
| **Familial Experiences Questionniare (FEQ)** |  | No | Child abuse | Sexual abuse; physical abuse | Before age 18 | NR | Yes/No | NO | Yes | NR | NR | Yes | NR | NR | Self-completion | NR | English | NR | NR | NR |
| **Family Background Questionnaire (FBQ)** |  | No | Child maltreatment | NR | Before age 18 | 179 | Never to always | No | Yes | No | NR | No | NR | 66.9 | Self-completion | NR | English | NR | NR | NR |
|  | **FBQ- Short Form (FBQ-SF)** | No | Child maltreatment | Parent responsiveness, physical and sexual abuse, physical neglect, parent substance abuse | Lifetime | 68 | 5 point Likert scale | NR | Yes | NR | Yes | Yes | NR | 66.9 | Self-completion | NR | English | NR | NR | NR |
|  | **FBQ- Uganda (FBQ-U)** | No | Child maltreatment | Psychological maltreatment; physical abuse; physical neglect; sexual abuse; educational neglect; substance abuse | NR | 94 | NR | NR | Yes | NR | NR | NR | NR | 66.9 | Self-completion | NR | English; Luganda | NR | NR | NR |
| **Interview for Traumatic Events in Childhood (ITEC)** |  | Yes | Child maltreatment | Sexual, physical, emotional abuse; emotional and physical neglect | Before age 18 | 33 | Yes/No | No | Yes | No | Yes | Yes | No | Interview | interviewer-led | NR | Dutch | Yes | No | NR |
| **ISPCAN Child Abuse Screening Tool - Retrospective (ICAST-R)** |  | Yes | Child abuse | Physical violence; psychological violence; sexual violence; neglect | Before age 18 | 15, V3 contains 20 | Yes/No | No | Yes | Yes | Yes | Yes | No | 71.6 | Self-completion paper-pencil questionnaire/ tablet or interviewers | 20 minutes | English; Russian; Arabic; Hindi; Marathi; Malay; Spanish; Korean; Sinhalese; Portucugese | Yes | No | NR |
| **Juvenile Victimization Questionnaire (JVQ)** |  | Yes | Childhood violence exposure | Conventional crime; child maltreatment; peer and sibling victimisation; sexual victimisation; witnessing and indirect victimisation | Before age 18 | 34 | Yes/No | Yes | Yes | Yes | Yes | Yes | No | 84.3 | Self-administered, interviewer-administered, computer-administered | 20-30 minutes | English; Spanish | Yes | Yes | Any experienced examiner can administer. Paraprofessionals and research assistants should administer under supervision.. |
| **Maltreatment and Abuse Chronology of Exposure (MACE)** |  | Yes | Child maltreatment | Verbal, emotional, physical and sexual abuse; emotional neglect; witnessing IPV and violence against siblings; peer verbal abuse, physical bullying | Before age 18 | 75 | Yes/No | No | Yes | No | No | Yes | No | 63.1 | Self-completion | NR | English; German Spanish | Yes | No | NR |
| **Negative Life Events Scale (NLES)** |  | Yes | Negative life events | Physical abuse; psychological abuse; sexual abuse; other traumatic events | Childhood | 74 (40 abuse items) | Yes/No | NR | NR | NR | Yes | NR | NR | 93.1 | Self-completion | NR | English | NR | NR | NR |
| **NorVold Abuse Questionnaire (NorAQ)** |  | Yes | Child abuse | Emotional, sexual, physical abuse; abuse in health care system | Before age 18 | 13 | Yes/No | Yes | Yes | Yes | No | Yes | No | 55.1 | Self-completion | NR | Swedish | NR | No | NR |
| **Parent Threat Inventory (PTI)** |  | Yes | Child abuse | Threat of rejection; threat of abandonment; threat of punishment/ neglect | Childhood | 17 | NR | No | Yes | No | Yes | Yes | No | 100 | Self-completion | NR | English | NR | No | NR |
| **Scale of Negative Family Interactions (SNFI)** |  | Yes | Emotional, physical, and sexual aggression from siblings and parents | Emotional aggression; physical aggression; sexual aggression | Lifetime | 34 items per subscale | 4 point Likert scale | No | Yes | No | Yes | Yes | No | 100 | Self-completion | 10-15 minutes | English | NR | No | NR |
| **Trauma Experience Checklist (TEC)** |  | Yes | Trauma | Interpersonal abuse and family stress; violence; death; legal involvement | Lifetime | 41 | Likert scale | No | No | No | Yes | Yes | No | 85.9 | Online or paper and pencil | NR | Dutch; English; French; German; Italian; Norwegian; Polish; Portugese; Swedish; Turkish; Urdu | Yes | No | NR |
| **Traumatic Antecedents Questionnaire (TAQ)** |  | Yes | Trauma | Neglect; separation; emotional, physical, and sexual abuse; witnessing IPV | Before age 18 | 40 | Never to very often | NR | Yes | No | Yes | Yes | Yes | 68 | Clinician led | NR | English; Hungarian; Korean | Yes | Yes | Recommended not be used in research and only in clinical practice |
| **Traumatic Events Questionnaire (TEQ)** |  | Yes | Child and adult physical and sexual abuse | Physical and sexual abuse | Lifetime | 49 | 7 point Likert scale | No | No | No | Yes | Yes | Yes | 66 | Self-completion paper-pencil | 5-10 minutes | English; Hebrew | Yes | No | NR |
| **Traumatic Events Survey (TES)** |  | No | Child sexual, physical and emotional abuse | Physical, sexual and emotional abuse | Before age 18 | 10 | Yes/No | NR | No | No | NR | Yes | No | NR | Self-completion paper-pencil | NR | English | NR | No | NR |
| **Traumatic Life Events Questionnaire (TLEQ)** |  | Yes | Trauma | Physical and sexual abuse | Lifetime | 24 | 5 point Likert scale | No | No | No | Yes | No | No | 94.1 | NR | NR | English; Spanish | NR | No | NR |
| **Only physical abuse** | | | | | | | | | | | | | | | | | | | | |
| **Assessing Environments III (AEIII)** |  | No | Physical discipline | Physical discipline | Lifetime | 170, 12 items on physical punishment | True of False | No | No | No | No | No | NR | NR | Self-completion | NR | English | NR | NR | NR |
| **Ast Physical Discipline Inventory – Early Childhood (APDI-EC)** |  | Yes | Physical discipline | Physical abuse | Age 0-7 | 42 | Never to daily | No | Yes | No | Yes | Yes | Yes | 65.7 | Self-completion | NR | English | Yes | No | NR |
|  | **Ast APDI – Late childhood (APDI-LC)** | Yes | Physical discipline | Physical abuse | Age 8-17 | 42 | Never to daily | No | Yes | No | Yes | Yes | Yes | 65.7 | Self-completion | NR | English | Yes | No | NR |
| **Violent Experiences Questionnaire (VEQ)** |  | Yes | Physical discipline and abuse | Corporal punishment; parent-child and sibling verbal discord; observed parental discord and violence; parent-child and sibling physical threats; observed parental threats; child-parent and sibling physical abuse; peer bullying; peer teasing | Age 5-16 | 36 | Never happened- happened more than once a week (9 options) | No | Yes | No | Yes | No | No | 24.9 | Self-completion | 10 minutes | English | NR | No | NR |
| **Only psychological abuse** | | | | | | | | | | | | | | | | | | | | |
| **Psychological Maltreatment Inventory (PMI)** |  | Yes | Psychological maltreatment | Emotional neglect; hostile rejection; isolation | Childhood | 25 | 5 point Likert scale | No | No | No | No | No | NR | 93.1 | Self-completion | 10 minutes | English | NR | No | NR |
| **Leipzig Incidence and Psychological Distress Questionnaire (LEBI)** |  | No | Stressful life events | Bereavement; social conflicts; serious disease; abuse | Childhood | 50 | NR | NR | Yes | NR | NR | No | No | 65.7 | Self-completion | NR | German | Yes | NR | NR |
| **Psychological Maltreatment Experiences Scale (PMES)** |  | Yes | Child maltreatment | Verbal abuse; neglectful parenting; withholding supportive behaviors; minimizing, isolating and terrorizing acts; exploitative parenting | Childhood | 53 | Never to very often | No | Yes | No | Yes | No | No | 75.9 | Self-completion | 15 minutes | English | NR | No | NR |
| **Psychological Maltreatment Review (PMR)** |  | Yes | Psychological maltreatment | psychological abuse, neglect, and support | Before age 18 | 30 | Never to over 20 times per year | No | Yes | No | Yes | No | No | 95.3 | Self-completion | 10 minutes | English | NR | NR | NR |
| **Verbal Abuse Questionnaire (VAQ)** |  | Yes | Verbal abuse | None | Childhood | 15 | Not at all to everyday | No | Yes | No | Yes | Yes | No | 100 | Self-completion | 10 minutes | English; Korean | NR | No | NR |
| **Only sexual abuse** | | | | | | | | | | | | | | | | | | | | |
| **Child Sexual Assault Scale (CSAS)** |  | Yes | Sexual abuse | None | Before age 14 | 14 | Yes/No | No | Yes | No | No | Yes | No | 50 | Self-completion | 5-10 minutes | Hebrew | NR | No | NR |
| **Childhood Sexual Abuse Interview (CSAI)** |  | No | Sexual abuse | None | Before age 18 | 17 | Yes/No | No | Yes | No | Yes | No | NR | NR | Interviewer-led | NR | English | NR | NR | NR |
| **Childhood Sexual Abuse Scale (CSAbS)** |  | No | Sexual abuse | None | NR | 12 | Never to frequent | NR | NR | NR | NR | NR | NR | NR | Self-completion | NR | NR | NR | NR | NR |
| **Childhood Sexual Experiences Scale (CSES1)** |  | Yes | Sexual abuse | None | Before age 18 | NR | Yes/No | No | Yes | No | No | No | NR | 26.7 | Self-completion | NR | English; Hebrew | NR | NR | NR |
| **Childhood Sexual Experiences Survey (CSES2)** |  | Yes | Sexual abuse | None | Before age 18 | 21 | Never to every day | No | Yes | No | Yes | Yes | No | 69.2 | Self-completion | 10-20 minutes | English | NR | No | NR |
| **Early Sexual Experiences Questionnaire (ESEQ)** |  | Yes | Sexual abuse | Sexual aubuse | Before age 17 | 3 | Yes/No | No | Yes | Yes | Yes | No | No | 67 | Self-completion | NR | English | NR | No | NR |
| **Finkelhor's Childhood Sexual Experiences Survey (FCSES)** |  | Yes | Sexual abuse | Sexual abuse | Before age of 12, after age of 12 | 14 | NR | No | Yes | Yes | Yes | Yes | No | 64.2 | Self-completion | NR | English | NR | No | NR |
| **Historical Events of Abuse Survey (HEAS)** |  | Yes | Sexual abuse | Sexual abuse | Childhood | 8 | NR | no | Yes | Yes | Yes | No | No | 77.2 | Self-completion | NR | English | NR | No | NR |
| **Life Experiences Qustionnaire (LEQ)** |  | Yes | Sexual abuse | NR | Before age 17 | 8 | Yes/No | NR | Yes | NR | No | Yes | NR | 70 | Self-completion | NR | English | NR | NR | NR |
| **Modified Sexual Events Questionnaire (SEQ)** |  | No | Sexual acts | NR | before age 14 | NR | Yes/No | NR | NR | NR | NR | NR | NR | NR | Self-completion | NR | English | NR | NR | NR |
| **Multi-dimensional Measure of Child Sexual Absue (MMCSA)** |  | Yes | Sexual abuse | Verbal coercion; physical coercion; sexual experiences | Before age 18 | 33 | unsure to very frequently | No | Yes | Yes | Yes | Yes | No | 68.8 | Self-completion | NR | English | NR | No | NR |
| **Russel Sexual Abuse Interview Schedule (RSAIS)** |  | Yes | Sexual abuse | Sexual abuse | before age 14 | 14 | Yes/No | No | Yes | No | No | No | No | 62.5 | Self-completion | NR | English | NR | No | NR |
| **Sexual Abuse Exposure Questionnaire (SAEQ)** |  | Yes | Sexual acts | Sexual abuse | Before age 18 | 10 | Yes/No | No | Yes | Yes | No | Yes | No | 62 | NR | 30-45 minutes | English | Yes | No | NR |
| **Sexual Abuse Questionnaire (SAQ1)** |  | Yes | Sexual abuse | NR | Before age 18 | 45 | Yes/No | No | No | No | No | No | No | 92.8 | Self-completion paper-pencil | 5 minutes | English | NR | No | NR |
| **Sexual Assault Questionnaire (SAQ2)** |  | No | Sexual abuse | NR | Lifetime | NR | NR | NR | Yes | NR | No | Yes | NR | NR | Self-completion paper-pencil | NR | English | NR | NR | NR |
| **Sexual Experiences Survey (SES)** |  | Yes | Sexual victimization | NR | Past 5 years | 5 or 10 | Yes/No | NR | NR | NR | No | Yes | No | 61.8 | Self-completion paper-pencil | NR | English | Yes | No | NR |
| **Sexual History Questionnaire (SHQ)** |  | Yes | Sexual abuse | Witnessing; unwanted sexual activity; unwanted sexual advances or sexual pressure; forced sexual encounter; other sexual trauma | Lifetime | 21 | NR | No | Yes | Yes | Yes | Yes | No | 76 | NR | NR | English | NR | No | NR |
| **Wyatt Sex History Questionnaire (WSHQ)** |  | Yes | Sexual abuse | NR | NR | 7 | NR | No | No | No | No | Yes | No | 84.7 | Audio Computer Assisted Self Interview | 90 minutes | English | NR | No | NR |
| **Only neglect** | | | | | | | | | | | | | | | | | | | | |
| **Multidimensional Neglectful Behaviour Scale (MNBS)** |  | Yes | Child neglect | Emotional, physical, cognitive, supervisory neglect | Childhood | 20 | 5 point Likert scale | No | Yes | No | Yes | No | No | 87.1 | Self-completion | NR | English; Chinese; Spanish; German; French; Portugese; Korean | NR | No | NR |
| **Neglect scale (NS)** |  | Yes | Child abuse | NR | Childhood | 63 | 4 point Likert scale | No | Yes | No | No | Yes | No | 100 | Self-completion | NR | English | Yes | No | NR |

**Table 2: Assessment of methodological quality of development and content validity studies**

|  |  | Development Study Quality | | | Content Validity Study Quality | | | | |  |  |
| --- | --- | --- | --- | --- | --- | --- | --- | --- | --- | --- | --- |
|  |  | Item generation | Cognitive Interview | | Asking target population | | | Asking Professionals | |  |  |
| Instrument | Reference | Relevance | Comprehensiveness | Comprehensibility | Relevance | Comprehensiveness | Comprehensibility | Relevance | Comprehensiveness | Language |  |
|  |  |  |  |  |  |  |  |  |  |  |  |
| Multiple forms of abuse | | | | | | | | | | |  |
| ICAST-R | Dunne et al 2009 | Inadequate | NR | NR | NR | NR | NR | NR | NR | English, Marathi, Russian, Arabic, Spanish |  |
|  | Silveira et al 2016 | NR | NR | NR | NR | NR | Doubtful | NR | NR | Portuguese |  |
|  | Chadnaratne et al 2018 | Doubtful | Inadequate | Inadequate | Doubtful | Doubtful | Doubtful | Doubtful | Doubtful | Sinhala |  |
| CMQ | Demare 2000 | Doubtful | NR | NR | NR | NR | NR | Doubtful | Doubtful | English |  |
| CCMI | Riddle 1998 | NR | NR | NR | NR | NR | Doubtful | NR | NR | English |  |
| DDI | Fauchier et al 2010 | NR | NR | NR | NR | Doubtful | NR | NR | NR | English |  |
| FBQ | Melchert et al 1998 | NR | NR | NR | NR | NR | NR | Doubtful | Doubtful | English |  |
| FBQ-U | Kalemeera 2007 | NR | NR | NR | NR | NR | Doubtful | Doubtful | NR | Luganda |  |
| ACE-IQ | Quinn et al., 2018 | NR | NR | NR | Very Good* | NR | Very good | NR | NR | English, South Africa |  |
| TLEQ | Villarroel et al. 2012 | NR | NR | NR | NR | NR | NR | Adequate | Adequate | Spanish |  |
| PTI | Scher et al. 2002 | Inadequate | Inadequate | Inadequate | NR | NR | NR | NR | NR | English |  |
| Only sexual abuse | | | | | | | | | | |  |
| MMCSA | Williams 2001 | NR | NR | NR | NR | NR | NR | Doubtful | Doubtful | English |  |

**Table 3: Quality of content validity for each included study on the development and content validity of an instrument**

| Instrument | Reference | Relevance | | | Comprehensiveness | | | Comprehensibility | | |
| --- | --- | --- | --- | --- | --- | --- | --- | --- | --- | --- |
|  |  | Development Study | Content Validity | Reviewer Rating | Development Study | Content Validity | Reviewer Rating | Development Study | Content Validity | Reviewer Rating |
| Multiple forms of abuse | | | | | | | | | | |
| ICAST-R | Dunne et al 2009 | + | ? | + | - | ? | + | - |  | + |
|  | Silveira et al 2016 | ? | ? | ? | ? | ? | ? | ? | + | ? |
|  | Chandnaratne et al 2018 | + | + | ? | + | + | ? | + | ? | ? |
| CMQ | Demare 2000 | + | ? | ? | + | ? | ? | ? | ? | ? |
| CCMI | Riddel 1998 | NE | NE | + | Ne | NE | ? | NE | ? | - |
| DDI | Fauchier et al 2010 | NR | ? | + | NE | + | + | NE | ? | + |
| FBQ | Melchert et al 1998 | NE | + | ? | NE | + | ? | NE | NE | ? |
| FBQ-U | Kalemeera 2007 | NR | + | ? | Ne | NE | ? | NE | + | ? |
| ACE-IQ | Quinn et al., 2018 | NR | + | + | NR | NR | + | NR | + | + |
| TLEQ | Villarroel et al. 2012 | NR | + | + | NR | + | + | NR | NR | NR |
| PTI | Scher et al. 2002 | ? | NR | + | ? | NR | ? | ? | NR | + |
| Only sexual abuse | | | | | | | | | | |
| MMCSA | Williams 2001 | NE | ? | + | NE | ? | + | NE | NE | NE |

**Table 4: Methodological and content validity quality assessment of development and content validity studies per instrument**

| **Instrument** | Relevance | | Comprehensiveness | | Comprehensibility | |
| --- | --- | --- | --- | --- | --- | --- |
|  | Overall quality of content validity | Quality of evidence | Overall quality of content validity | Quality of evidence | Overall quality of content validity | Quality of evidence |
| Multiple forms of abuse | | | | | | |
| ICAST-R | + | Very low | ± | Very low | ± | moderate |
| CMQ | + | Very low | + | Very low | ? | Very low |
| CCMI | + | very low | ? | Very low | ? | Very low |
| DDI | ? | Very low | + | low | ? | Very low |
| FBQ | + | Moderate | + | Moderate | NE | NE |
| FBQ-U | + | Moderate | NE | NE | + | Moderate |
| ACE-IQ | + | High | NR | NR | + | High |
| TLEQ | + | Moderate | + | Moderate | NR | NR |
| PTI | ? | Very low | ? | Very low | ? | Very low |
| Only sexual abuse | | | | | | |
| MMCSA | + | Moderate | + | Moderate | NE | NE |

+sufficient rating, ? indeterminate rating, -insufficient rating, ± inconsistent rating

**Table 5: Methodological Quality Assessment of Psychometric studies**

| Instrument | Reference | Structural validity | Internal consistency | Cross-cultural validity | Reliability | Criterion Validity | Hypothesis testing | Concordance | Language |
| --- | --- | --- | --- | --- | --- | --- | --- | --- | --- |
| Multiple forms of abuse | | | | | | | | | |
| ICAST-R | Dunne et al 2009 | NR | Very good | NR | NR | NR | NR | NR | English, Russian, Arabic, Spanish, Marathi |
|  | Eldeeb et al 2009 | NR | Very good | NR | NR | NR | NR | NR | Arabic |
|  | Lee et al 2011 | NR | Very good | NR | NR | NR | Doubtful | NR | Korean |
|  | Jangam et al 2015 | NR | NR | NR | NR | NR | Doubtful | NR | Hindi |
|  | Chandraratne et al 2018 | Adequate | Very good | NR | Inadequate | NR | NR | NR | Sinhala |
| APK | Ehrenthal et al 2020 | NR | Very good | NR | NR | NR | Very good | NR | German |
| ACE- BRFSS | Ford et al 2014 | Very good | Very good | Doubtful | NR | NR | NR | NR | English |
|  | Walsh et al 2014 | NR | NR | NR | NR | NR | Adequate | NR | English |
|  | Campbell et al 2016 | NR | NR | NR | NR | NR | Doubtful | NR | English |
|  | Font et al 2016 | NR | NR | NR | NR | NR | Adequate | NR | English |
| VICA | Claridge et al 2014 | NR | NR | NR | NR | NR | Very good | NR | English |
|  | Bizzarro et al 2003 | NR | NR | NR | NR | NR | Adequate | NR | English |
| CATS | Sanders et al 1995 | Adequate | Very good | NR | Inadequate | NR | Adequate | NR | English |
|  | Kennedy et al 2003 | NR | Very good | NR | NR | NR | NR | NR | English |
|  | Hocking et al 2016 | NR | Inadequate | NR | NR | NR | NR | NR | NR |
|  | Harmer et al 1999 | NR | Very good | NR | NR | NR | Very good | NR | English |
|  | Hahn et al 2016 | NR | Inadequate | NR | NR | NR | Adequate | NR | English |
|  | Mondragon 2005 | NR | Inadequate | NR | NR | NR | Doubtful | NR | English |
|  | Goldsmith 2004 | NR | NR | Inadequate | NR | NR | Adequate | NR | English |
|  | Rankin 1999 | NR | NR | NR | NR | NR | Adequate | NR | NR |
|  | Grossman 1997 | NR | NR | NR | NR | NR | Adequate | NR | English |
|  | Kent et al 1998 | NR | Very good | NR | NR | NR | Very good | NR | English |
|  | Jackson 2004 | NR | NR | NR | NR | NR | Very good | NR | English |
|  | Kroll et al 1996 | NR | NR | NR | NR | NR | Very good | NR | English |
|  | Nakai et al 2014 | NR | NR | NR | NR | NR | Very good | NR | Japanese |
|  | Tesno et al 2013 | NR | Very good | NR | NR | NR | Adequate | NR | Japanese |
|  | Hayashi et al 2015 | NR | NR | NR | NR | NR | Adequate | NR | Japanese |
|  | Toda et al 2016 | NR | NR | NR | NR | NR | Adequate | NR | Japanese |
|  | Ono et al 2017 | NR | NR | NR | NR | NR | Very good | NR | Japanese |
| CAEI | Jakupcevic et al 2011 | NR | Doubtful | NR | NR | NR | Very good | NR | Croatian |
| CMH-SR | Gartland et al 2016 | NR | NR | NR | NR | NR | Very good | NR | English |
|  | Macmillan et al 2001 | NR | NR | NR | NR | NR | Very good | NR | English |
|  | Mancini et al 1995 | NR | NR | NR | NR | NR | Doubtful | NR | English |
| CMIS-SF | Nuckols 2010 | NR | NR | NR | NR | NR | Doubtful | NR | English |
|  | Rankin 1999 | NR | NR | NR | NR | NR | Adequate | NR | English |
|  | Birchfield 1996 | NR | NR | NR | NR | NR | Very good | NR | English |
|  | Jacobs 1998 | NR | NR | NR | NR | NR | Adequate | NR | English |
|  | Fiore 1997 | NR | NR | NR | NR | NR | Doubtful | NR | English |
|  | Thabet et al 2004 | NR | NR | NR | NR | NR | Very good | NR | Arabic |
|  | Dietrich 2003 | NR | NR | NR | NR | NR | Doubtful | NR | English |
|  | Dunn 1994 | NR | NR | NR | NR | NR | Inadequate | NR | NR |
| CEVQ | Tanaka et al 2012 | NR | Very good | NR | Doubtful | NR | Very good | NR | English |
|  | Fuller-Thomson et al 2016 | NR | NR | NR | NR | NR | Very good | NR | English/French |
| CEVQ-SF | Tanaka et al 2012 | NR | Very good | NR | Doubtful | Very good | Very good | Adequate | English/French |
|  | Tardif-Williams et al 2017 | NR | Very good | NR | NR | NR | Very good | NR | English |
| CMQ | Marcy 1998 | NR | NR | NR | NR | NR | Very good | NR | English |
|  | Demare 2000 | Adequate | Very good | NR | Inadequate | NR | Adequate | NR | English |
| CES | Mersky 2017 | Adequate | Very good | NR | Doubtful | Very good | NR | NR | English |
| CHQ1 (Milner 1995) | Litty et al 1996 | NR | inadequate | NR | NR | NR | NR | NR | English |
|  | Lamela et al 2013 | NR | Very good^1^ | NR | NR | NR | NR | NR | Portuguese |
|  | Figuerido et al 2004 | NR | Inadequate | NR | NR | NR | NR | NR | Portuguese |
| CHQ2 (Felitti 1987) | Pereira da silva et al 2013 | NR | NR | NR | Doubtful | NR | Inadequate | NR | Portuguese |
| CTI | Fink 1996 | Doubtful | NR | NR | Adequate | NR | very good | NR | English |
|  | Spinhoven et al 2014 | NR | NR | NR | NR | NR | Adequate | NR | Dutch |
| CTQ-SF | Marquee-Flintje 2017 | NR | very good | NR | NR | NR | Very good | NR | English |
|  | Arnow 2011 | NR | Very good | NR | NR | NR | Very good | NR | English |
|  | Bernstein et al 2003 | Very good | Very good | Doubtful | NR | NR | Very good | Very good | English |
|  | Sacchi 2017 | Very good | Very good | NR | NR | NR | Very good | NR | Italian |
|  | Schmidt 2020 | NR | NR | NR | NR | NR | Adequate | Adequate | English, Spanish |
|  | Van Deusen et al 2007 | NR | very good | NR | NR | NR | NR | NR | English |
|  | Verona et al 2016^2^ | NR | very good | NR | NR | NR | Very good | NR | English |
|  | Banducci et al 2014^3^ | NR | Very good | NR | NR | NR | Very good | NR | English |
|  | Grillo et al 2006 | NR | NR | NR | NR | NR | Very good | NR | English |
|  | Bernstein et al 1998 | Very good | NR | NR | NR | NR | NR | NR | English |
|  | Carr et al 2010 | NR | very good | NR | Doubtful | NR | Adequate | NR | English |
|  | Yuan et al 2006 | NR | NR | NR | NR | NR | Doubtful | NR | English |
|  | Burton 2008 | NR | very good | NR | NR | NR | Very good | NR | English |
|  | Kuo et al 2015 | NR | very good | NR | NR | NR | NR | NR | English |
|  | Rohlehr 2014^1^ | NR | NR | NR | NR | NR | Very good | NR | English |
|  | Kazeem et al. 2015 | NR | very good | NR | NR | NR | Very good | NR | English (Nigeria) |
|  | Brockie et al 2015 | NR | very good | NR | NR | NR | Very good | NR | English |
|  | Thombs et al 2007 | NR | very good | Inadequate | NR | NR | NR | NR | English |
|  | Banducci et al 2014^3^ | NR | very good | NR | NR | NR | Adequate | NR | English |
|  | Ammerman et al 2016 | NR | NR | NR | NR | NR | Adequate | NR | English |
|  | Andreopulous 2002 | NR | NR | NR | NR | NR | Adequate | NR | English |
|  | DiLillo et al 2010 | NR | NR | NR | NR | NR | NR | Adequate | English |
|  | Powers et al 2011 | NR | NR | NR | NR | NR | Very good | NR | English |
|  | Bailey et al 2012 | NR | Very good | NR | NR | NR | NR | Very good | English |
|  | Cammack et al 2016 | NR | Doubtful | NR | Inadequate | NR | NR | NR | English |
|  | Messman-Moore et al 2004^4^ | NR | Very good | NR | NR | NR | Adequate | NR | English |
|  | DiLillo et al 2006 | NR | NR | NR | NR | NR | NR | Very good | English |
|  | Villano et al 2004 | Adequate | Doubtful | NR | NR | NR | NR | NR | English |
|  | Rosen 1996 | NR | Inadequate | NR | NR | NR | Adequate | Inadequate | English |
|  | Rosen 1998 | NR | Very good | NR | NR | NR | NR | NR | English |
|  | Galea 2012 | NR | Doubtful | NR | NR | NR | NR | NR | English |
|  | Festinger et al 2010^5^ | NR | very good | NR | NR | NR | NR | NR | English |
|  | Grewal-Sandh 2008 | NR | very good | NR | NR | NR | Doubtful | NR | English |
|  | Kimball 2003 | NR | very good | NR | NR | NR | Very good | NR | English |
|  | Virkler 2005 | NR | NR | NR | NR | NR | Adequate | NR | English |
|  | Wright Wimberley 2004 | NR | NR | NR | NR | NR | Adequate | NR | English |
|  | Twomey 1997 | NR | NR | NR | NR | NR | Adequate | NR | English |
|  | Forde et al 2012 | Very good | Very good | Doubtful | NR | NR | NR | NR | English |
|  | Boillat et al 20017 | NR | NR | NR | NR | NR | Very good | NR | English |
|  | Duran et al 2004 | NR | NR | NR | NR | NR | Doubtful | NR | English |
|  | Jewkes et al 2016 | NR | NR | NR | NR | NR | Very good | NR | isiXhosa |
|  | Fitzhenry et al 2015 | NR | Very good | NR | NR | NR | Very good | NR | English |
|  | wildes et al 2008 | NR | NR | NR | NR | NR | Adequate | NR | English |
|  | Simon et al 2009 | NR | NR | NR | NR | NR | Very good | NR | English |
|  | Bradley et al 2005 | NR | Doubtful | NR | NR | NR | Doubtful | NR | English |
|  | Dunn 2009 | Adequate | Inadequate | NR | NR | NR | NR | NR | English |
|  | Narayan 2009 | NR | NR | NR | NR | NR | Very good | NR | English |
|  | Wright et al 2001 | NR | NR | Doubtful | NR | NR | NR | NR | English |
|  | Spitzer et al 2006 | NR | NR | NR | NR | NR | NR | Inadequate | German |
|  | Beutel et al 2017 | NR | NR | NR | NR | NR | Very good | NR | German |
|  | Schilling et al 2016 | NR | very good | NR | NR | NR | NR | NR | German |
|  | Thombs et al 2009 | Very good | very good | Very good | NR | NR | Very good | NR | Dutch |
|  | Weibel et al 2017 | NR | NR | NR | NR | NR | Very good | NR | French |
|  | Fuchs et al 2016 | NR | Very good | NR | NR | NR | NR | NR | German |
|  | Gil et al 2009 | NR | Very good | NR | Doubtful | NR | NR | NR | Portuguese |
|  | Rajkumar 2015 | NR | NR | NR | NR | NR | Doubtful | NR | Tamil |
|  | Allen et al 1998 | NR | NR | NR | NR | NR | Very good | NR | German |
|  | Bailer et al 2014 | NR | Inadequate | NR | NR | NR | Adequate | NR | German |
|  | Machisa et al 2016 | NR | Inadequate | NR | NR | NR | Doubtful | NR | English, Afrikaans, isiZulu, SeSotho |
|  | Spitzer et al 2012 | NR | NR | NR | NR | NR | Very good | NR | German |
|  | Pompili et al 2009 | NR | NR | NR | NR | NR | Adequate | NR | Italian |
|  | Lotzin et al 2016 | NR | NR | NR | NR | NR | Adequate | NR | German |
|  | Fosse et al 2007 | NR | Doubtful | NR | NR | NR | NR | NR | Norwegian |
|  | Voorthuis et al 2014 | NR | Very good | NR | NR | NR | NR | NR | Dutch |
|  | Pompili et al 2014 | NR | Very good | NR | NR | NR | Very good | NR | Italian |
|  | Kim et al 2011 | Adequate | Very good | NR | Doubtful | NR | NR | Very good | Korean |
|  | Kong et al 2009 | NR | Very good | NR | NR | NR | Adequate | NR | Korean |
|  | Jewkes et al 2016 | NR | NR | NR | NR | NR | Adequate | NR | isiXhosa |
|  | Kim et al 2013 | NR | Very good | NR | Doubtful | NR | Adequate | Very good | Korean |
|  | Bonevski et al 2012 | NR | NR | NR | NR | NR | Doubtful | NR | Macedonian |
|  | Bahk et al 2017 | NR | NR | NR | NR | NR | Very good | NR | Macedonian |
|  | Li et al 2017 | NR | NR | NR | NR | NR | Very good | NR | Chinese |
|  | Saracli et al 2016 | NR | NR | NR | NR | NR | Very good | NR | Turkish |
|  | Senkal et al 2015 | NR | NR | NR | NR | NR | Very good | NR | Turkish |
|  | Reider et al 2013 | NR | Very good | NR | NR | NR | Adequate | NR | Kiryawandan |
|  | Sfoggia et al 2008 | NR | NR | NR | NR | NR | Very good | NR | Portuguese |
|  | Sarchiapone et al 2009 | NR | very good | NR | NR | NR | Adequate | NR | Italian, French |
|  | Spinhoven et al 2014 | Very good | very good | Doubtful | NR | NR | Adequate | Very good | Dutch |
|  | Jennissen et al 2016 | NR | very good | NR | NR | NR | Very good | NR | German |
|  | Kuhlman et al 2013 | NR | very good | NR | NR | NR | Very good | NR | German |
|  | Schulz et al 2014 | NR | NR | NR | NR | NR | Very good | NR | German |
|  | Karos et al 2014 | Very good | Very good | NR | NR | NR | Very good | NR | German |
|  | Dovran et al 2013 | Very good | Very good | Doubtful | NR | NR | NR | NR | Norwegian |
|  | Naqavi et al 2011 | NR | NR | NR | NR | NR | Very Good | NR | Farsi |
|  | Hernandez et al 2013 | Adequate | Very good | NR | NR | NR | NR | NR | Spanish |
|  | Gerdner et al 2009 | Adequate | Very good | NR | NR | NR | NR | NR | Swedish |
| CTA | Kristjanson et al 2016 | Very good | NR | NR | NR | NR | Inadequate | NR | English |
| CCMS | Higgins et al 2001 | Adequate | Very good | NR | Doubtful | NR | Very good | NR | English |
|  | Allen 2011 | NR | Very good | NR | NR | NR | Very good | NR | English |
|  | Allen et al 2013 | NR | Very good | NR | NR | NR | NR | NR | English |
| CCMI | Riddle 1998 | NR | Very good | NR | Adequate | NR | NR | NR | English |
|  | Riddle et al 1999 | NR | Very good | NR | Doubtful | NR | NR | NR | English |
| CAMI | DiLillo et al 2010 | NR | Inadequate | NR | Doubtful | NR | very good | NR | English |
|  | DiLillo et al 2006 | NR | NR | nR | nR | NR | NR | Adequate | English |
|  | Nash et al 2012 | Adequate | Very good | NR | NR | NR | NR | NR | English |
|  | Clemmons 2004 | NR | NR | NR | NR | NR | very good | NR | English |
| CTS-PC | Leary et al 2008 | NR | NR | NR | NR | NR | Inadequate | NR | English |
|  | Lambert 2010 | NR | Inadequate | NR | NR | NR | very good | NR | English |
|  | Sims et al 2008 | NR | Very good | NR | NR | NR | Doubtful | NR | English |
|  | Miller-Perrin et al 2009 | NR | Very good | NR | NR | NR | very good | NR | English |
|  | Merrill 2001 | NR | Very good | NR | NR | NR | very good | NR | English |
|  | McCarty 1999 | NR | NR | NR | NR | NR | very good | NR | English |
|  | Corvo 1993 | NR | Very good | NR | NR | NR | very good | NR | English |
|  | Adamson 1997 | NR | NR | NR | NR | NR | very good | NR | English |
|  | Wolfner 1996 | Adequate | NR | NR | NR | NR | NR | NR | English |
| DDI | Fauchier et al 2010 | Very good | Very good | NR | Doubtful | NR | Very good | NR | English |
|  | Van Leeuwen et al 2012 | Very good | Very good | NR | NR | NR | Very good | NR | Dutch |
| ETI-SF | Singh et al 2012 | NR | NR | NR | NR | NR | Adequate | NR | English |
|  | Hyman et al 2005 | NR | Very good | NR | NR | NR | Doubtful | Adequate | English |
|  | Plaza et al 2011 | NR | Very good | NR | Doubtful | NR | Adequate | NR | Spanish |
|  | Horberg et al 2019 | Very good | Very good | NR | Adequate | NR | Very good | NR | Swedish |
|  | Osorio et al 2013 | Very good | Very good | NR | Adequate | NR | Very good | NR | Portuguese |
|  | Jeon et al 2012 | Very good | Very good | NR | Doubtful | NR | Adequate | NR | Korean |
|  | Bremner et al 2007 | Adequate | Very good | NR | NR | Very good | Doubtful | NR | English |
| ETI | Bremner et al 2007 | Inadequate | Very good | NR | NR | NR | Doubtful | NR | English |
|  | Bremner et al 2000 | NR | NR | NR | NR | Doubtful | NR | Adequate | English |
|  | Karanovic et al 2017 | NR | NR | NR | NR | NR | Adequate | NR | Croatian |
|  | Merza et al 2015^6^ | NR | Very good | NR | NR | NR | Very good | NR | Hungarian |
|  | Plaza et al 2011 | NR | Very good | NR | Doubtful | NR | Adequate | NR | Spanish |
|  | Antonopoulou et al 2017 | Adequate | Very good | NR | Adequate | NR | Very good | NR | Greek |
| EASE-PI | Nicholas et al 1997 | Doubtful | NR | NR | Inadequate | NR | Inadequate | NR | English |
|  | Milletich et al 2010^4^ | NR | Inadequate | NR | NR | NR | Very good | NR | English |
| FBQ-U | Kalemeera 2007 | Very good | Very good | Adequate | Doubtful | NR | Very good | NR | Luganda |
| FBQ | Melchert et al 1998 | NR | Doubtful | NR | Doubtful | NR | Doubtful | NR | English |
| FBQ-SF | Melchert et al 2009 | Very good | Very good | NR | NR | NR | Doubtful | NR | English |
| FEQ | Durrett et al 2004 | NR | NR | NR | Inadequate | NR | Doubtful | NR | English |
| ITEC | Lobbestael et al 2009 | Very good | Very good | NR | Very good | NR | Very good | Very good | Dutch |
| MACE | Teicher et al 2015 | Very good | NR | NR | Doubtful | NR | Very good | Very good | English |
|  | Schalinski et al 2016 | NR | NR | NR | NR | NR | Doubtful | NR | German |
| NLES | Pitzner et al 1997 | Inadequate | Very good | NR | Doubtful | NR | Very good | NR | English |
| TAQ | Merza et al 2015 | NR | NR | NR | NR | NR | Inadequate | NR | Hungarian |
|  | Park et al 2020 | Very good | Very Good | NR | Doubtful | NR | Very good | Very good | Korean |
| TEC | Cristofaro et al. 2013 | Adequate | Very good | NR | NR | NR | Very good | NR | English |
| TES | Briere et al. 2016 | NR | NR | NR | NR | NR | Very good | NR | English |
| TEQ | Lipschitz et al. 1996 | NR | NR | NR | NR | NR | Very good | Doubtful | English |
|  | Thomson and Jaque 2015 | NR | NR | NR | Doubtful | NR | NR | NR | English |
|  | Lev-Wiesel and Daphna Tekoa 2007 | NR | Very good | NR | NR | NR | Very good | NR | Hebrew |
|  | Yampolsky et al. 2010 | NR | Doubtful | NR | NR | NR | NR | NR | Hebrew |
|  | Lev-Wiesel and Markus 2013 | NR | Very good | NR | NR | NR | NR | NR | Hebrew |
| CECA | Craig et al. 2000 | NR | NR | NR | NR | NR | Adequate | NR | English |
|  | Gerra et al. 2016 | NR | NR | NR | NR | NR | Very Good | NR | Italian |
|  | Lara et al. 2015 | NR | NR | NR | NR | NR | Very Good | NR | Spanish |
|  | Tousignant et al. 2011 | NR | NR | NR | NR | NR | Doubtful | NR | English and French |
|  | Fisher et al. 2011 | NR | NR | NR | Doubtful | NR | Doubtful | Very good | English |
|  | Bifulco et al. 2002; | NR | NR | NR | NR | NR | Doubtful | NR | English |
|  | Smith et al. 2002; | NR | Very Good | NR | Doubtful | Inadequate | NR | NR | English |
|  | (Gerra et al. 2014) | NR | NR | NR | NR | NR | Very good | NR | Italian |
|  | Bifulco et al. 2005 | NR | Very good | NR | Adequate | NR | Very good | Adequate | English |
| ACE-ASF | Wade et al. 2017 | NR | NR | NR | NR | Inadequate | Doubtful | NR | English |
|  | Hughes et al. 2016; | NR | NR | NR | NR | NR | Very good | NR | English |
|  | Chegeni et al. 2020 | Very good | Very good | NR | NR | NR | Very good | NR | Persian |
|  | Jewkes et al. 2010 | NR | Very good | NR | NR | NR | Very good | NR | isiXhosa |
| ACE-IQ | Tran et et al. 2015 | NR | NR | NR | NR | NR | Very good | NR | Vietnamese |
|  | Mhamdi et al. 2017 | NR | NR | NR | NR | NR | Very good | NR | NR (Study conducted in Tunisia( |
|  | Kazeem et al. 2015 | NR | Very good | NR | NR | NR | NR | Very good | English |
|  | Kim et al. 2017 | NR | NR | NR | NR | NR | Very good | NR | Korean |
|  | Quinn et al. 2018 | NR | NR | NR | NR | NR | NR | NR | English |
| ACE-S | Afifi et al. 2017 | Very good | NR | NR | NR | NR | Very good | NR | English |
| ACE | Love 2011 | NR | NR | NR | NR | NR | Very good | NR | English |
|  | Bellis et al. 2013 | NR | NR | NR | NR | NR | Very good | NR | English |
|  | Poole et al. 2017 | NR | NR | NR | NR | NR | Very good | NR | English |
|  | Chapman et al. 2004 | NR | NR | NR | NR | NR | Doubtful | NR | English |
|  | Patterson et al. 2014 | NR | NR | NR | NR | NR | Very good | NR | English |
|  | Roh et al. 2015 | NR | Very good | NR | NR | NR | Very good | NR | English |
|  | Ramiro et al. 2010 | NR | NR | NR | NR | NR | Inadequate | NR | Filipino |
|  | Swopes et al. 2013 | NR | Inadequate | NR | NR | NR | Adequate | NR | English |
|  | Rausch 2016 | NR | NR | NR | NR | NR | Very good | NR | English |
|  | McGinn 2015 | NR | NR | NR | NR | NR | Very good | NR | English |
|  | Strine 2010 | NR | NR | NR | NR | NR | Very good | NR | English |
|  | Bruskas 2012 | NR | NR | NR | NR | NR | Very good | NR | English |
|  | Whitfield et al. 2003 | NR | NR | NR | NR | NR | Inadequate | NR | English |
|  | Youssef et al. 2017 | NR | NR | NR | NR | NR | Very good | NR | English |
|  | Park et al. 2015 | NR | NR | NR | NR | NR | Adequate | NR | Korean |
|  | Burnette et al. 2017 | NR | Very good | NR | NR | NR | Adequate | NR | English |
|  | Mair et al. 2012 | NR | Very good | NR | NR | NR | Very good | NR | English |
|  | Cabrera et al. 2007 | NR | NR | NR | NR | NR | Adequate | NR | English |
|  | Welles et al. 2017 | NR | NR | NR | NR | NR | Adequate | NR | English |
|  | Pflugradt et al. 2018 | NR | NR | NR | NR | NR | Very good | NR | English |
|  | Dube et al. 2004 | NR | NR | NR | NR | NR | Adequate | NR | English |
|  | Anda et al. 2002 | NR | NR | NR | NR | NR | Doubtful | NR | English |
|  | Kaier et al. 2015 | NR | NR | NR | NR | NR | Adequate | NR | English |
|  | Edwards et al. 2001 | NR | NR | NR | NR | NR | Inadequate | NR | English |
|  | Basto-Pereira et al. 2016 | NR | NR | NR | NR | NR | Inadequate | NR | Portuguese |
|  | Bruskas and Tessin et al. 2013 | NR | NR | NR | NR | NR | Adequate | NR | English |
|  | Honkalampi et al. 2005 | NR | NR | NR | NR | NR | Adequate | NR | NR (in Finland) |
|  | Chung et al. 2008 | NR | NR | NR | NR | NR | Very good | NR | English |
|  | Hung et al. 2013 | NR | NR | NR | NR | Very good | Very good | NR | NR (in Taiwan) |
|  | Montgomery et al. 2013 | NR | NR | NR | NR | NR | Adequate | NR | English |
|  | Gahm et al. 2007 | NR | NR | NR | NR | NR | Very good | NR | English |
|  | Epperson et al. 2017 | NR | NR | NR | NR | NR | Very good | NR | English |
|  | Levenson and Grady 2016 | NR | NR | NR | NR | NR | Adequate | NR | English |
|  | McCall-Hosenfeld et al. 2014 | NR | NR | NR | NR | NR | Adequate | NR | English |
|  | Schilling et al. 2007 | NR | NR | NR | NR | NR | Adequate | NR | English |
|  | Sacco et al. 2007 | NR | NR | NR | NR | NR | Adequate | NR | English |
|  | Giovanelli et al. 2016 | NR | NR | NR | NR | Nr | Very good | NR | English |
|  | Dong et al. 2004 | NR | Doubtful | NR | NR | NR | NR | NR | English |
|  | De Ravello et al. 2008 | NR | NR | NR | NR | NR | Doubtful | NR | English |
| TLEQ | Villarroel et al. 2012 | NR | NR | NR | Adequate | NR | NR | NR | Spanish |
|  | Pereda and Gallardo-Pujol 2014 | NR | NR | NR | NR | NR | Very good | NR | Spanish |
| CARTS | Simonelli et al., 2017 | NR | Adequate | Adequate | NR | NR | Very good | NR | Italian |
| CTQ | Arata 2002 | NR | NR | NR | NR | NR | Very good | NR | English |
|  | Reuben 2016 | NR | NR | NR | NR | NR | Doubtful | Very good | English |
|  | DAngelo 2006 | NR | NR | NR | NR | NR | Very good | NR | English |
|  | Ferrari 2002 | NR | Inadequate | NR | NR | NR | Inadequate | NR | English |
|  | Karakus 2012 | NR | Inadequate | NR | NR | NR | NR | NR | Turkish |
|  | Dong 2004 | NR | NR | NR | NR | NR | Very good | NR | English |
|  | Paivio 2004 | Adequate | Very good | NR | Doubtful | NR | NR | NR | English |
|  | Bernet 1999 | NR | NR | NR | NR | NR | Adequate | NR | English |
|  | Rosen 1998 | NR | Very good | NR | NR | NR | NR | NR | English |
|  | Paivio 2001 | NR | NR | NR | Inadequate | NR | Doubtful | NR | English |
| CTQ 30 item significantly modified | Rosen 1996 | NR | Inadequate | NR | NR | NR | Adequate | Inadequate | English |
| JVQ | Charak 2016 | NR | Inadequate | NR | NR | NR | Very good | NR | Spanish |
|  | Karatekin & Ahluwalia 2016 | Very good | Inadequate | NR | NR | NR | Doubtful | NR | English |
| NorAQ | Swahnberg 2003 | NR | NR | NR | Doubtful | NR | NR | Doubtful | Swedish |
|  | Swahnberg 2011 | NR | NR | NR | Doubtful | NR | NR | Very good | Swedish |
| PTI | Scher et al. 2002 | Inadequate | Doubtful | NR | Doubtful | NR | Adequate | NR | English |
| SNFI | Simonelli et al. 2005 | NR | Doubtful | NR | Adequate | NR | Doubtful | NR | English |
| Only physical abuse | | | | | | | | | |
| AEIII | Scioli-Salter 2016 | NR | Very good | NR | NR | NR | Very good | NR | English |
|  | Berger et al 1988 | NR | NR | nR | NR | NR | Very good | NR | English |
| APDI-EC | Ast 2006 | Inadequate | Very good | NR | NR | NR | Very good | NR | English |
| APDI-LC | Ast 2006 | Inadequate | Very good | NR | NR | NR | Very good | NR | English |
| VEQ | King and Russell 2017 | Very good | NR | NR | Inadequate | NR | Adequate | NR | English |
|  | King 2014 | NR | NR | NR | NR | NR | Adequate | NR | English |
| Only psychological abuse | | | | | | | | | |
| PMES | Lopez-Stane 2006 | NR | Very good | NR | NR | NR | Very good | NR | English |
| PMI | Jackson 2004 | NR | NR | NR | NR | NR | Very good | NR | English |
|  | Swift and Grayton 1996 | NR | NR | NR | NR | NR | Doubtful | NR | English |
| PMR | Briere et al. 2012 | Very good | Very good | NR | NR | NR | NR | NR | English |
| LEBI | Nickel et al 2004 | NR | NR | NR | NR | NR | Doubtful | NR | German |
| VAQ | Jeong et al. 2015 | Adequate | Very good | NR | NR | NR | Very good | NR | Korean |
| Only sexual abuse | | | | | | | | | |
| CSAI | Andreopulous 2002 | NR | NR | NR | NR | NR | Inadequate | NR | English |
| CSAbS | Marshall et al 1995 | NR | NR | NR | NR | NR | Doubtful | NR | English |
| CSES1 | Lev-Wiesel et al 2007 | NR | Very good | NR | NR | NR | very good | NR | Hebrew |
| CSES2 | Mallow 2000 | NR | NR | NR | NR | NR | Doubtful | NR | English |
| CSAS | Yampolsky et al 2010 | NR | Very good | NR | NR | NR | Very Good | NR | Hebrew |
|  | Lev-Wiesel et al 2013 | NR | NR | NR | NR | NR | Very good | NR | Hebrew |
| SHQ | Steel et al. 2004 | NR | NR | NR | NR | NR | Adequate | NR | English |
| SAEQ | Gauble 2010 | NR | Very good | NR | NR | NR | NR | NR | English |
|  | Scioli-Salter et al. 2016 | NR | Very good | NR | NR | NR | Adequate | NR | English |
|  | Ryan 1993 | NR | NR | NR | Very good | NR | NR | NR | English |
| SAQ 1 | Lock et al. 2005 | Very good | Very good | NR | Very good | Adequate | NR | NR | English |
| SAQ 2 | Zlotnick et al. 1996 | NR | NR | NR | NR | NR | Very good | NR | NR |
| SES | Karabatsos 1997 | NR | NR | NR | NR | NR | Very good | NR | English |
| MMCSA | Williams 2001 | NR | NR | NR | Doubtful | NR | Doubtful | NR | English |
| FCSES | Shchupak 2011 | NR | NR | NR | NR | NR | Very good | Very good | English |
|  | Rosen et al 1996 | NR | NR | NR | NR | NR | Very good | NR | English |
|  | Craig 2002 | NR | NR | NR | NR | NR | Inadequate | NR | English |
| RSAIS | Shchupak 2011 | NR | NR | NR | NR | NR | Very good | Very good | English |
| HEAS | Edwards 1997 | Doubtful | Doubtful | NR | NR | NR | NR | NR | English |
| ESEQ | Cooper 1995 | NR | NR | NR | NR | NR | Doubtful | NR | English |
| SEQ | Merrill 2001 | NR | NR | NR | NR | NR | Very good | NR | English |
| LEQ | Messman-Moore et al 2000 | NR | very good | NR | NR | NR | Doubtful | NR | English |
|  | Messman-Moore et al 2004 | NR | NR | NR | NR | NR | Very good | NR | English |
|  | Long 2002 | NR | NR | NR | Very good | NR | NR | NR | English |
| WSHQ | Allen et al. 2014 | NR | NR | NR | NR | NR | Adequate | NR | English |
| Only neglect | | | | | | | | | |
| MNBS | Straus 2006 | NR | Very good | Very good | NR | NR | Very good | NR | English (translated for 17 different countries) |
| NS | Harrington et al. 2002 | Very good | Very good | NR | NR | NR | NR | NR | English |

**Table 6: Quality of the psychometric properties of each included study**

| Instrument | Reference | Structural validity | Internal consistency | Cross-cultural validity | Reliability | Criterion Validity | Hypothesis testing | Concordance |
| --- | --- | --- | --- | --- | --- | --- | --- | --- |
| Multiple forms of abuse | | | | | | | | |
| ICAST-R | Dunne et al 2009 | NR | - | NR | NR | NR | NR | NR |
|  | Eldeeb et al 2016 | NR | - | NR | NR | NR | NR | NR |
|  | Lee et al 2011 | NR | - | NR | NR | NR | + | NR |
|  | Jangam et al 2016 | NR | NR | NR | NR | NR | + | NR |
|  | Chandraratne et al 2018 | ? | - | NR | + | NR | NR | NR |
| APK | Ehrenthal et al 2020 | NR | + | NR | NR | NR | + | NR |
| ACE- BRFSS | Ford et al 2014 | + | + |  | NR | NR | NR | NR |
|  | Walsh et al 2014 | NR | NR | NR | NR | NR | + | NR |
|  | Campbell et al 2016 | NR | NR | NR | NR | NR | + | NR |
|  | Font et al 2016 | NR | NR | NR | NR | NR | + | NR |
| VICA | Claridge et al 2014 | NR | NR | NR | NR |  | + | NR |
|  | Bizzarro et al 2003 | NR | NR | NR | NR |  | - | NR |
| CTS-PC | Leary et al 2008 | NR | ? | NR | NR | NR | + | NR |
|  | Sims et al 2008 | NR | + | NR | NR | NR | + | NR |
|  | Lambert 2010 | NR | + | NR | NR | NR | + | NR |
|  | Miller-Perrin et al 2009 | NR | + | NR | NR | NR | + | NR |
|  | Merrill 2001 | NR | + | NR | NR | NR | + | NR |
|  | McCarty 1999 | NR | NR | NR | NR | NR | ± | NR |
|  | Corvo 1993 | NR | + | NR | NR | NR | + | NR |
|  | Adamson 1997 | NR | NR | NR | NR | NR | - | NR |
|  | Wolfner 1996 | ? | NR | NR | NR | NR | NR | NR |
| CATS | Sanders et al 1995 | ? | ± | NR | ? | NR | + | NR |
|  | Kennedy et al 2003 | NR | + | NR | NR | NR | NR | NR |
|  | Hocking et al 2016 | NR | ? | NR | NR | NR | NR | NR |
|  | Harmer et al 1999 | NR | ± | NR | NR | NR | + | NR |
|  | Hahn et al 2016 | NR | ? | NR | NR | NR | - | NR |
|  | Mondragon 2005 | NR | ? | NR | NR | NR | + | NR |
|  | Goldsmith 2004 | NR | NR | ? | NR | NR | + | NR |
|  | Rankin 1999 | NR | NR | NR | NR | NR | + | NR |
|  | Grossman 1997 | NR | NR | NR | NR | NR | + | NR |
|  | Kent et al 1998 | NR | ± | NR | NR | NR | + | NR |
|  | Jackson 2004 | NR | NR | NR | NR | NR | + | NR |
|  | Kroll et al 1996 | NR | NR | NR | NR | NR | + | NR |
|  | Nakai et al 2014 | NR | NR | NR | NR | NR | + | NR |
|  | Tesno et al 2013 | NR | + | NR | NR | NR | + | NR |
|  | Hayashi et al 2015 | NR | NR | NR | NR | NR | + | NR |
|  | Toda et al 2016 | NR | NR | NR | NR | NR | + | NR |
|  | Ono et al 2017 | NR | NR | NR | NR | NR | + | NR |
| CAEI | Jakupcevic et al 2011 | NR | - | NR | NR | NR | + | NR |
| CMH-SR | Gartland et al 2016 | NR | NR | NR | NR | NR | + | NR |
|  | Macmillan et al 2001 | NR | NR | NR | NR | NR | + | NR |
|  | Mancini et al 1995 | NR | NR | NR | NR | NR | + | NR |
| CMIS-SF | Nuckols 2010 | NR | NR | NR | NR | NR | + | NR |
|  | Rankin 1999 | NR | NR | NR | NR | NR | + | NR |
|  | Birchfield 1996 | NR | NR | NR | NR | NR | + | NR |
|  | Jacobs 1998 | NR | NR | NR | NR | NR | - | NR |
|  | Fiore 1997 | NR | NR | NR | NR | NR | + | NR |
|  | Thabet et al 2004 | NR | NR | NR | NR | NR | + | NR |
|  | Dietrich 2003 | NR | NR | NR | NR | NR | - | NR |
|  | Dunn 1994 | NR | NR | NR | NR | NR | - | NR |
| CEVQ | Tanaka et al 2012 | NR | + | NR | + | NR | + | NR |
|  | Fuller-Thomson et al 2016 | NR | NR | NR | NR | NR | + | NR |
| CEVQ-SF | Tanaka et al 2012 | NR | + | NR | ± | + | + | - |
|  | Tardif-Williams et al 2017 | NR | + | NR | NR | NR | + | NR |
| CMQ | Marcy 1998 | NR | NR | NR | NR | NR | - | NR |
|  | Demare 2000 | ? | + | NR | ? | NR | + | NR |
| CES | Mersky 2017 | + | + | NR | + | + | NR | NR |
|  |  |  |  |  |  |  |  |  |
| CTI | Fink 1996 | - | NR | NR | + | NR | + | NR |
|  | Spinhoven et al 2014 | NR | NR | NR | NR | NR | + | NR |
| CTQ-SF^a^ | Marquee-Flentje 2017 | NR | + | NR | NR | NR | + | NR |
|  | Arnow 2011 | NR | + | NR | NR | NR | + | NR |
|  | Sacchi 2017 | + | + | NR | NR | NR | + | NR |
|  | Schmidt 2020 | NR | NR | NR | NR | NR | + | + |
|  | Stoltz et al 2007 | NR | + | NR | NR | NR | NR | NR |
|  | van Deusen et al 2007 | NR | ± | NR | NR | NR | NR | NR |
|  | Verona et al 2016^2^ | NR | + | NR | NR | NR | + | NR |
|  | Banducci et al 2014^3^ | NR | + | NR | NR | NR | + | NR |
|  | Grillo et al 2006 | NR | NR | NR | NR | NR | + | NR |
|  | Bernstein et al 1998 | - | NR | NR | NR | NR | NR | NR |
|  | Carr et al 2010 | NR | + | NR | ? | NR | + | NR |
|  | Yuan et al 2006 | NR | NR | NR | NR | NR | + | NR |
|  | Burton 2008 | NR | + | NR | NR | NR | + | NR |
|  | Kuo et al 2015 | NR | + | NR | NR | NR | NR | NR |
|  | Rohlehr 2014^1^ | NR | NR | NR | NR | NR | + | NR |
|  | Kazeem 2015 | NR | ± | NR | NR | NR | + | NR |
|  | Brockie et al 2015 | NR | ± | NR | NR | NR | + | NR |
|  | Thombs et al 2007 | NR | ± | + | NR | NR | NR | NR |
|  | Banducci et al 2014^3^ | NR | + | NR | NR | NR | + | NR |
|  | Ammerman et al 2016 | NR | NR | NR | NR | NR | - | NR |
|  | Andreopulous 2002 | NR | NR | NR | NR | NR | + | NR |
|  | DiLillo et al 2010 | NR | NR | NR | NR | NR | NR | ± |
|  | Powers et al 2011 | NR | NR | NR | NR | NR | + | NR |
|  | Bailey et al 2012 | NR | + | NR | NR | NR | NR | ± |
|  | Cammack et al 2016 | NR | ± | NR | + | NR | NR | NR |
|  | Messmann-Moore et al 2004^4^ | NR | + | NR | NR | NR | - | NR |
|  | DiLillo et al 2006 | NR | NR | NR | NR | NR | NR | ± |
|  | Villano et al 2004 | - | ± | NR | NR | NR | NR | NR |
|  | Rosen 1996 | NR | ± | NR | NR | NR | + | - |
|  | Rosen 1998 | NR | ± | NR | NR | NR | NR | NR |
|  | Galea 2012 | NR | ± | NR | NR | NR | NR | NR |
|  | Festinger et al 2010^5^ | NR | + | NR | NR | NR | NR | NR |
|  | Grewal-Sandh 2008 | NR | ± | NR | NR | NR | + | NR |
|  | Kimball 2003 | NR | ± | NR | NR | NR | - | NR |
|  | Virkler 2005 | NR | NR | NR | NR | NR | + | NR |
|  | Wright Wimberley 2004 | NR | NR | NR | NR | NR | + | NR |
|  | Twomey 1997 | NR | NR | NR | NR | NR | + | NR |
|  | Forde et al 2012 | + | ± | + | NR | NR | NR | NR |
|  | Boillat et al 2017 | NR | NR | NR | NR | NR | + | NR |
|  | Bernstein et al 2003 | + | + | + | NR | NR | + | ± |
|  | Duran et al 2004 | NR | NR | NR | NR | NR | + | NR |
|  | Jewkes et al 2016 | NR | NR | NR | NR | NR | + | NR |
|  | Fitzhenry et al 2015 | NR | + | NR | NR | NR | + | NR |
|  | Wildes et al 2008 | NR | NR | NR | NR | NR | + | NR |
|  | Simon et al 2009 | NR | NR | NR | NR | NR | + | NR |
|  | Bradley et al 2005 | NR | + | NR | NR | NR | ? | NR |
|  | Dunn 2009 | ? | + | NR | NR | NR | NR | NR |
|  | Narayan 2009 | NR | NR | NR | NR | NR | + | NR |
|  | Wright et al 2001 | NR | NR | - | NR | NR | NR | NR |
|  | Spitzer et al 2006 | NR | NR | NR | NR | NR | NR | - |
|  | Beutel et al 2017 | NR | NR | NR | NR | NR | + | NR |
|  | Schilling et al 2016 | NR | ± | NR | NR | NR | NR | NR |
|  | Thombs et al 2009 | + | ± | + | NR | NR | + | NR |
|  | Weibel et al 2017 | NR | NR | NR | NR | NR | + | NR |
|  | Fuchs et al 2016 | NR | ± | NR | NR | NR | NR | NR |
|  | Gil et al 2009 | NR | ± | NR | ± | NR | NR | NR |
|  | Rajkumar 2015 | NR | NR | NR | NR | NR | + | NR |
|  | Allen et al 1998 | NR | NR | NR | NR | NR | + | NR |
|  | Bailer et al 2014 | NR | ± | NR | NR | NR | ± | NR |
|  | Machisa et al 2016 | NR | ? | NR | NR | NR | - | NR |
|  | Spitzer et al 2012 | NR | NR | NR | NR | NR | + | NR |
|  | Pompili et al 2009 | NR | NR | NR | NR | NR | + | NR |
|  | Lotzin et al 2016 | NR | NR | NR | NR | NR | + | NR |
|  | Fosse et al 2007 | NR | ± | NR | NR | NR | NR | NR |
|  | Voorthuis et al 2014 | NR | ± | NR | NR | NR | NR | NR |
|  | Pompili et al 2014 | NR | ± | NR | NR | NR | + | NR |
|  | Kim et al 2011 | ? | ± | NR | ? | NR | NR | ± |
|  | Kong et al 2009 | NR | ± | NR | NR | NR | + | NR |
|  | Jewkes et al 2010 | NR | NR | NR | NR | NR | + | NR |
|  | Kim et al 2013 | NR | ± | NR | ? | NR | + | - |
|  | Bonevski et al 2012 | NR | NR | NR | NR | NR | + | NR |
|  | Bahk et al 2017 | NR | NR | NR | NR | NR | + | NR |
|  | Li et al 2017 | NR | NR | NR | NR | NR | + | NR |
|  | Saracli et al 2016 | NR | NR | NR | NR | NR | + | NR |
|  | Senkal et al 2015 | NR | NR | NR | NR | NR | + | NR |
|  | Reider et al 2013 | NR | ± | NR | NR | NR | + | NR |
|  | Sfoggia et al 2008 | NR | NR | NR | NR | NR | + | NR |
|  | Sarchiapone et al 2009 | NR | ± | NR | NR | NR | ± | NR |
|  | Spinhoven et al 2014 | + | ± | + | NR | NR | + | - |
|  | Jennissen et al 2016 | NR | + | NR | NR | NR | + | NR |
|  | Kuhlman et al 2013 | NR | ± | NR | NR | NR | + | NR |
|  | Schulz et al 2014 | NR | NR | NR | NR | NR | + | NR |
|  | Karos et al 2014 | + | ± | NR | NR | NR | + | NR |
|  | Dovran et al 2013 | + | + | + | NR | NR | NR | NR |
|  | Naqavi et al 2011 | NR | NR | NR | NR | NR | + | NR |
|  | Hernandez et al 2013 | + | ± | NR | NR | NR | NR | NR |
|  | Gerdner et al 2009 | ? | ± | NR | NR | NR | NR | NR |
| CTA | Kristjansson et al 2016 | + | NR | NR | NR | NR | + | NR |
| CCMS | Higgins et al 2001 | ? | ± | NR | ? | NR | + | NR |
|  | Allen 2011 | NR | + | NR | NR | NR | + | NR |
|  | Allen et al 2013 | NR | + | NR | NR | NR | NR | NR |
| CCMI | Riddle 1998 | NR | - | NR | ? | NR | NR | NR |
|  | Riddle et al 1999 | NR | - | NR | + | NR | NR | NR |
| CAMI | DiLillo et al 2010 | NR | ? | NR | ± | NR | + | NR |
|  | DiLillo et al 2006^2^ | NR | NR | NR | NR | NR | NR | + |
|  | Nash et al 2012 | ? | + | NR | NR | NR | NR | NR |
|  | Clemmons 2004 | NR | NR | NR | NR | NR | + | NR |
| DDI | Fauchier et al 2010 | - | + | NR | + | NR | + | NR |
|  | Van Leeuwen et al 2012 | + | - | NR | NR | NR | + | NR |
| ETI-SF | Singh et al 2012 | NR | NR | NR | NR | NR | + | NR |
|  | Hyman et al 2005 | NR | + | NR | NR | NR | + | ± |
|  | Plaza et al 2011 | NR | ± | NR | + | NR | + | NR |
|  | Jeon et al 2012 | + | - | NR | + | NR | + | NR |
|  | Horberg et al 2019 | - | ± | NR | + | NR | + | NR |
|  | Osorio et al 2013 | - | ± | NR | + | NR | + | NR |
|  | Bremner et al 2007 | ? | + | NR | NR | + | + | NR |
| ETI | Bremner et al 2007 | ? | + | NR | NR | NR | + | NR |
|  | Bremner et al 2000 | NR | NR | NR | NR | + | NR | - |
|  | Karanovic et al 2017 | NR | NR | NR | NR | NR | + | NR |
|  | Merza et al 2015^6^ | NR | + | NR | NR | NR | + | NR |
|  | Plaza et al 2011 | NR | ± | NR | + | NR | + | NR |
|  | Antonopoulou et al 2017 | ? | + | NR | + | NR | + | NR |
| EASE-PI | Nicholas et al 1997 | ? | NR | NR | ? | NR | ? | NR |
|  | Milletich et al 2010^4^ | NR | + | NR | NR | NR | + | NR |
| FBQ-U | Kalemeera 2007 | - | + | + | ? | NR | + | NR |
| FBQ | Melchert et al 1998 | NR | - | NR | ? | NR | + | NR |
| FBQ-SF | Melchert et al 2009 | + | + | NR | NR | NR | ? | NR |
| FEQ | Durret et al 2004 | NR | NR | NR | - | NR | + | NR |
| ITEC | Lobbestael et al 2009 | - | ± | NR | + | NR | + | - |
| MACE | Teicher et al 2015 | ? | NR | NR | ? | NR | + | ± |
|  | Schalinski et al 2016 | NR | NR | NR | NR | NR | + | NR |
| NLES | Pitzner et al 1997 | ? | + | NR | ? | NR | + | NR |
| TAQ | Merza et al2015 | NR | NR | NR | NR | NR | + | NR |
|  | Park et al 2020 | + | ± | NR | ? | NR | + | + |
| TEC | Cristofaro et al 2013 | + | + | NR | NR | NR | ? | NR |
| TES | Briere et al 2016 | NR | NR | NR | NR | NR | + | NR |
| TEQ | Lipschitz et al 1996 | NR | NR | NR | NR | NR | + | + |
|  | Thomson and Jaque 2015 | NR | NR | NR | + | NR | NR | NR |
|  | Lev-Wiesel and Daphna Tekoa 2007 | NR | + | NR | NR | NR | - | NR |
|  | Yampolsky et al 2010 | NR | + | NR | NR | NR | NR | NR |
|  | Lev-Wiesel and Markus 2013 | NR | + | NR | NR | NR | NR | NR |
| CECA | Craig et al 2000 | NR | NR | NR | NR | NR | + | NR |
|  | Gerra et al. 2016 | NR | NR | NR | NR | NR | + | NR |
|  | Lara et al 2015 | NR | NR | NR | NR | NR | + | NR |
|  | Tousignant et al 2011 | NR | NR | NR | NR | NR | - | NR |
|  | Fisher et al 2011 | NR | NR | NR | - | NR | - | + |
|  | Bifulco et al 2002 | NR | NR | NR | NR | NR | + | NR |
|  | Smith et al 2002 | NR | + | NR | + | ? | NR | NR |
|  | Gerra et al. 2014 | NR | NR | NR | NR | NR | + | NR |
|  | Bifulco et al 2005 | NR | + | NR | + | NR | + | + |
| ACE-ASF | Wade et al. 2017 | NR | NR | NR | NR | ± | ? | NR |
|  | Hughes et al 2016 | NR | NR | NR | NR | NR | + | NR |
|  | Chegeni et al 2020 | + | + | NR | NR | NR | + | NR |
|  | Jewkes et al 2010 | NR | + | NR | NR | NR | + | NR |
| ACE-IQ | Tran et et al. 2015 | NR | NR | NR | NR | NR | + | NR |
|  | Mhamdi et al 2017 | NR | NR | NR | NR | NR | ? | NR |
|  | Kazeem et al. 2015 | NR | + | NR | NR | NR | NR | + |
|  | Kim et al. 2017 | NR | NR | NR | NR | NR | + | NR |
|  | Quinn et al 2018 | NR | NR | NR | NR | NR | NR | NR |
| ACE-S | Afifi et al 2017 | + | NR | NR | NR | NR | + | NR |
| ACE | Love 2011 | NR | NR | NR | NR | NR | + | NR |
|  | Bellis et al 2013 | NR | NR | NR | NR | NR | + | NR |
|  | Poole et al 2017 | NR | NR | NR | NR | NR | + | NR |
|  | Chapman et al 2004 | NR | NR | NR | NR | NR | + | NR |
|  | Patterson et al 2014 | NR | NR | NR | NR | NR | + | NR |
|  | Roh et al 2015 | NR | - | NR | NR | NR | + | NR |
|  | Ramiro et al 2010 | NR | NR | NR | NR | NR | + | NR |
|  | Swopes et al 2013 | NR | ? | NR | NR | NR | + | NR |
|  | Rausch 2016 | NR | NR | NR | NR | NR | + | NR |
|  | McGinn 2015 | NR | NR | NR | NR | NR | + | NR |
|  | Strine 2010 | NR | NR | NR | NR | NR | + | NR |
|  | Bruskas 2012 | NR | NR | NR | NR | NR | + | NR |
|  | Whitfield et al 2003 | NR | NR | NR | NR | NR | + | NR |
|  | Youssef et al 2017 | NR | NR | NR | NR | NR | + | NR |
|  | Park et al 2015 | NR | NR | NR | NR | NR | + | NR |
|  | Burnette et al. 2017 | NR | - | NR | NR | NR | + | NR |
|  | Mair et al 2012 | NR | - | NR | NR | NR | + | NR |
|  | Cabrera et al 2007 | NR | NR | NR | NR | NR | + | NR |
|  | Welles et al 2017 | NR | NR | NR | NR | NR | + | NR |
|  | Pflugradt et al 2018 | NR | NR | NR | NR | NR | + | NR |
|  | Dube et al 2004 | NR | NR | NR | NR | NR | ? | NR |
|  | Anda et al 2002 | NR | NR | NR | NR | NR | + | NR |
|  | Kaier et al 2015 | NR | NR | NR | NR | NR | + | NR |
|  | Edwards et al 2001 | NR | NR | NR | NR | NR | + | NR |
|  | Basto-Pereira et al 2016 | NR | NR | NR | NR | NR | ± | NR |
|  | Bruskas et al 2013 | NR | NR | NR | NR | NR | + | NR |
|  | Honkalampi et al 2005 | NR | NR | NR | NR | NR | - | NR |
|  | Chung et al 2008 | NR | NR | NR | NR | NR | + | NR |
|  | Hung et al 2013 | NR | NR | NR | NR | + | + | NR |
|  | Montgomery et al 2013 | NR | NR | NR | NR | NR | + | NR |
|  | Gahm et al 2007 | NR | NR | NR | NR | NR | + | NR |
|  | Epperson et al 2017 | NR | NR | NR | NR | NR | + | NR |
|  | Levenson and Grady 2016 | NR | NR | NR | NR | NR | ± | NR |
|  | McCall-Hosenfeld et al 2014 | NR | NR | NR | NR | NR | ± | NR |
|  | Schilling et al 2007 | NR | NR | NR | NR | NR | + | NR |
|  | Sacco et al 2007 | NR | NR | NR | NR | NR | ± | NR |
|  | Giovanelli et al 2016 | NR | NR | NR | NR | Nr | + | NR |
|  | Dong et al 2004 | NR | ? | NR | NR | NR | NR | NR |
|  | De Ravello et al 2008 | NR | NR | NR | NR | NR | + | NR |
| TLEQ | Villarroel et al 2012 | NR | NR | NR | + | NR | NR | NR |
|  | Pereda and Gallardo-Pujol 2014 | NR | NR | NR | NR | NR | ? | NR |
| CARTS | Simonelli et al 2017 | NR | ± | + | NR | NR | + | NR |
| CTQ | Arata 2002 | NR | NR | NR | NR | NR | + | NR |
|  | Reuben 2016 | NR | NR | NR | NR | NR | + | + |
|  | DAngelo 2006 | NR | NR | NR | NR | NR | + | NR |
|  | Ferrari 2002 | NR | - | NR | NR | NR | + | NR |
|  | Karakus 2012 | NR | + | NR | NR | NR | NR | NR |
|  | Dong 2004 | NR | NR | NR | NR | NR | + | NR |
|  | Paivio 2004 | + | - | NR | + | NR | NR | NR |
|  | Rosen 1998 | NR | -^a^ | NR | NR | NR | NR | NR |
|  | Bernet 1999 | NR | NR | NR | NR | NR | + | NR |
|  | Paivio 2001 | NR | NR | NR | + | NR | - | NR |
| CTQ 30 item significantly modified | Rosen 1996 | NR | -^a^ | NR | NR | NR | + | - |
| JVQ | Charak 2016 | NR | ? | NR | NR | NR | + | NR |
|  | Karatekin & Ahluwalia 2016 | + | ? | NR | NR | NR | + | NR |
| NorAQ | Swahnberg 2003 | NR | NR | NR | + | NR | NR | + |
|  | Swahnberg 2011 | NR | NR | NR | ? | NR | NR | + |
| PTI | Scher et al 2002 | ? | ? | NR | ? | NR | + | NR |
| SNFI | Simonelli et al 2005 | NR | + | NR | + | NR | ? | NR |
| Only Physical abuse | | | | | | | | |
| AEIII | Scioli-Salter 2016 | NR | - | NR | NR | NR | + | NR |
|  | Berger et al 1988 | NR | NR | NR | NR | nR | + | NR |
| APDI-EC | Ast 2006 | - | + | NR | NR | NR | + | NR |
| APDI-LC | Ast 2006 | - | + | NR | NR | NR | + | NR |
| CHQ1 | Litty et al 1996 | NR | ? | NR | NR | NR | NR | NR |
|  | Lamela et al 2013 | NR | ± | NR | NR | NR | NR | NR |
|  | Figuerido et al 2004 | NR | - | NR | NR | NR | NR | NR |
| CHQ2 | Pereira da silva et al 2013 | NR | NR | NR | - | NR | - | NR |
| VEQ | King and Russell 2017 | + | NR | NR | ? | NR | ? | NR |
|  | King 2014 | NR | NR | NR | NR | NR | ? | NR |
| Only psychological abuse | | | | | | | | |
| LEBI | Nickel et al 2004 | NR | NR | NR | NR | NR | ? | NR |
| PMES | Lopez-Stane 2006 | NR | - | NR | NR | NR | + | NR |
| PMI | Jackson 2004 | NR | NR | NR | NR | NR | + | NR |
|  | Swift and Grayton 1996 | NR | NR | NR | NR | NR | + | NR |
| PMR | Briere et al 2012 | + | + | NR | NR | NR | NR | NR |
| VAQ | Jeong et al 2015 | ± | + | NR | NR | NR | + | NR |
| Only sexual abuse | | | | | | | | |
| CSAS | Yampolsky et al 2010 | NR | + | NR | NR | NR | + | NR |
|  | Lev-Wiesel et al 2013 | NR | NR | NR | NR | NR | + | NR |
| CSAI | Andreopulous 2002 | NR | NR | NR | NR | NR | + | NR |
| CSAbS | Marshall et al 1995 | NR | NR | NR | NR | NR | + | NR |
| CSES1 | Lev-Wiesel et al 2007 | NR | + | NR | NR | NR | - | NR |
| CSES2 | Mallow 2000 | NR | NR | NR | NR | NR | - | NR |
| ESEQ | Cooper 1995 | NR | NR | NR | NR | NR | + | NR |
| FCSES | Shchupak 2011 | NR | NR | NR | NR | NR | + | + |
|  | Rosen 1996 | NR | NR | NR | NR | NR | + | NR |
|  | Craig 2002 | NR | NR | NR | NR | NR | + | NR |
| RSAIS | Shchupak 2011 | NR | NR | NR | NR | NR | + | + |
| HEAS | Edwards 1997 | - | - | NR | NR | NR | NR | NR |
| LEQ | Messman-Moore 2000 | NR | + | NR | NR | NR | - | NR |
|  | Long 2002 | NR | NR | NR | ± | NR | NR | NR |
|  | Messman-Moore 2004 | NR | NR | NR | NR | NR | + | NR |
| MMCSA | Williams 2001 | NR | NR | NR | ? | NR | + | NR |
| SEQ | Merrill 2001 | NR | NR | NR | NR | NR | + | NR |
| SAQ 1 | Lock et al 2005 | + | + | NR | + | + | NR | NR |
| SAQ2 | Zlotnick et al 1996 | NR | NR | NR | NR | NR | ? | NR |
| SES | Karabatsos 1997 | NR | NR | NR | NR | NR | + | NR |
| WSHQ | Allen et al 2014 | NR | NR | NR | NR | NR | + | NR |
| SHQ | Steel et al 2004 | NR | NR | NR | NR | NR | + | NR |
| SAEQ | Gauble 2010 | NR | + | NR | NR | NR | NR | NR |
|  | Scioli-Salter et al 2016 | NR | + | NR | NR | NR | ? | NR |
|  | Ryan 1993 | NR | NR | NR | + | NR | NR | NR |
| Only neglect | | | | | | | | |
| MNBS | Straus 2006 | NR | ± | + | NR | NR | ± | NR |
| NS | Harrington et al 2002 | + | + | NR | NR | NR | NR | NR |

**Table 7: Overall quality of the psychometric properties and evidence quality per instrument**

| Instrument | Structural validity | | Internal consistency | | Cross-cultural validity | | Reliability | | Criterion Validity | | Hypothesis testing | | Concordance | |
| --- | --- | --- | --- | --- | --- | --- | --- | --- | --- | --- | --- | --- | --- | --- |
|  | Overall rating | Quality of Evidence | Overall rating | Quality of Evidence | Overall rating | Quality of Evidence | Overall rating | Quality of Evidence | Overall rating | Quality of Evidence | Overall rating | Quality of Evidence | Overall rating | Quality of Evidence |
| Multiple forms of abuse | | | | | | | | | | | | | | |
| ACE | NR | NR | - | High | NR | NR | NR | NR | + | High | + | High | NR | NR |
| ACE-ASF | + | High | + | High | NR | NR | NR | NR | ± | Very low | + | Moderate | NR | NR |
| ACE-BRFSS | + | High | + | High | + | Moderate | NR | NR | NR | NR | + | High | NR | NR |
| ACE-IQ | NR | NR | + | High | NR | NR | NR | NR | NR | NR | ? | High | + | High |
| ACE-S | + | High | NR | NR | NR | NR | NR | NR | NR | NR | + | High | NR | NR |
| APK | NR | NR | + | High | NR | NR | NR | NR | NR | NR | + | High | NR | NR |
| CAEI | NR | NR | ? | Low | NR | NR | NR | NR | NR | NR | + | Moderate | NR | NR |
| CAMI | ? | Moderate | + | High | NR | NR | ± | NR | NR | NR | + | High | + | Moderate |
| CARTS | NR | NR | ± | Moderate | + | Moderate | NR | NR | NR | NR | + | High | NR | NR |
| CATS | ? | High | ± | High | ? | Very low | ? | Very low | NR | NR | + | High | NR | NR |
| CCMI | NR | NR | - | High | NR | NR | ? | Low | NR | NR | NR | NR | NR | NR |
| CCMS | ? | Moderate | + | High | NR | NR | ? | Low | NR | NR | + | High | NR | NR |
| CECA | NR | NR | + | High | NR | NR | ± | Moderate | ? | Low | + | Moderate | + | High |
| CES | + | Moderate | + | High | NR | NR | + | Low | + | high | NR | NR | NR | NR |
| CEVQ | NR | NR | + | High | NR | Nr | + | Low | NR | NR | + | High | NR | NR |
| CEVQ-SF | NR | NR | + | High | NR | NR | ± | NR | + | High | + | High | - | Moderate |
| CHQ1 | NR | NR | ± | NR | NR | NR | NR | NR | NR | NR | NR | NR | NR | NR |
| CHQ2 | NR | NR | NR | NR | NR | NR | - | very low | NR | NR | - | Very low | Nr | NR |
| CMH-SR | NR | NR | NR | NR | NR | NR | NR | NR | NR | NR | + | High | NR | NR |
| CMIS-SF | NR | NR | NR | NR | NR | NR | NR | NR | NR | NR | ± | Low | NR | NR |
| CMQ | ? | Moderate | NR | NR | NR | NR | ? | Moderate | NR | NR | NR | NR | - | High |
| CTA | + | High | NR | NR | NR | NR | NR | NR | NR | NR | + | Very low | NR | NR |
| CTI | - | Low | NR | NR | NR | NR | + | Moderate | NR | NR | + | High | NR | NR |
| CTQ | + | Moderate | + | Moderate | NR | NR | + | Low | NR | NR | + | High | + | Moderate |
| CTQ-SF | ± | NR | + | Moderate | + | High | ? | Moderate | NR | NR | + | High | - | Moderate |
| CTS-PC | ? | Moderate | + | HIgh | NR | NR | NR | NR | NR | NR | + | High | NR | NR |
| DDI | ± | NR | ± | NR | NR | NR | + | Low | NR | NR | + | High | NR | NR |
| EASE-PI | ? | Low | + | Very low | NR | NR | ? | Very low | NR | NR | + | Moderate | NR | NR |
| ETI | ? | Moderate | + | Moderate | NR | NR | + | Moderate | + | low | + | High | - | Moderate |
| ETI-SF | ± | NR | + | Moderate | NR | NR | + | Moderate | + | High | + | High | ± | Moderate |
| FBQ | NR | NR | - | Low | NR | NR | ? | Low | NR | NR | + | Low | NR | NR |
| FBQ-SF | + | High | + | High | NR | NR | NR | NR | NR | NR | ? | Low | NR | NR |
| FBQ-U | - | High | + | High | + | Moderate | ? | Low | NR | Nr | + | High | NR | NR |
| FEQ | NR | NR | NR | NR | NR | NR | - | very low | NR | NR | + | Low | NR | NR |
| ICAST-R | ? | High | - | High | NR | NR | + | Very low | NR | NR | + | Moderate | NR | NR |
| ITEC | - | High | ± | NR | NR | NR | + | High | NR | NR | + | High | - | high |
| JVQ | + | High | ? | Low | NR | NR | NR | NR | NR | NR | + | High | NR | NR |
| MACE | ? | High | NR | NR | NR | NR | ? | Low | NR | NR | + | High | ± | NR |
| NLES | ? | Very low | + | High | NR | NR | ? | Low | NR | NR | + | HIgh | NR | NR |
| NorAQ | NR | NR | NR | NR | NR | NR | + | Low | NR | NR | NR | NR | + | Moderate |
| PTI | ? | Very low | ? | Low | NR | NR | ? | Low | NR | NR | + | Moderate | NR | NR |
| SNFI | NR | NR | + | Low | NR | NR | + | Moderate | NR | NR | ? | Low | NR | NR |
| TAQ | + | High | ± | NR | NR | NR | ? | Low | NR | NR | + | High | + | high |
| TEC | + | Moderate | + | High | NR | NR | NR | NR | NR | NR | ? | High | NR | NR |
| TEQ | NR | NR | + | Moderate | NR | NR | + | Low | NR | NR | ± | High | + | Low |
| TES | NR | NR | NR | NR | NR | NR | NR | NR | NR | NR | + | High | NR | NR |
| TLEQ | NR | NR | NR | NR | NR | NR | + | Moderate | NR | NR | ? | High | NR | NR |
| VICA | NR | NR | NR | NR | NR | NR | NR | NR | NR | NR | + | High | NR | NR |
| Only physical abuse | | | | | | | | | | | | | | |
| AEIII | NR | NR | - | High | NR | NR | NR | NR | NR | NR | + | High | NR | NR |
| APDI-EC | - | Very low | + | High | NR | NR | NR | NR | NR | NR | + | High | NR | NR |
| APDI-LC | - | Very low | + | High | NR | NR | NR | NR | NR | NR | + | High | NR | NR |
| VEQ | + | High | NR | NR | NR | NR | ? | Low | NR | NR | ? | Moderate | NR | NR |
| Only psychological abuse | | | | | | | | | | | | | | |
| LEBI | NR | NR | NR | NR | NR | NR | NR | NR | NR | NR | ? | Low | NR | NR |
| PMES | NR | NR | - | High | NR | NR | NR | NR | NR | NR | + | High | NR | NR |
| PMI | NR | NR | NR | NR | NR | NR | NR | NR | NR | NR | + | Low | NR | NR |
| PMR | + | High | + | High | NR | NR | NR | NR | NR | NR | NR | NR | NR | NR |
| VAQ | ± | Moderate | + | High | NR | NR | NR | NR | NR | NR | + | High | NR | NR |
| Only sexual abuse | | | | | | | | | | | | | | |
| CSAbS | NR | NR | NR | NR | NR | NR | NR | NR | NR | NR | + | Very low | NR | NR |
| CSAI | NR | NR | NR | NR | NR | NR | NR | NR | NR | NR | + | Very low | NR | NR |
| CSAS | NR | NR | + | High | NR | NR | NR | NR | NR | NR | + | High | NR | NR |
| CSES1 | NR | NR | + | High | NR | NR | NR | NR | NR | NR | - | High | NR | NR |
| CSES2 | NR | NR | NR | NR | NR | NR | NR | NR | NR | NR | - | Low | NR | NR |
| ESEQ | NR | NR | NR | NR | NR | NR | NR | NR | NR | NR | + | Low | NR | NR |
| FCSES | NR | NR | NR | NR | NR | NR | NR | NR | NR | NR | + | High | + | High |
| HEAS | - | Low | - | Low | NR | NR | NR | NR | NR | NR | NR | NR | NR | NR |
| LEQ | NR | NR | + | High | NR | NR | ± | NR | NR | NR | ± | NR | NR | NR |
| MMCSA | NR | NR | NR | NR | NR | NR | ? | Low | NR | NR | + | Low | NR | NR |
| RSAIS | NR | NR | NR | NR | NR | NR | NR | NR | NR | NR | + | High | + | High |
| SAEQ | NR | NR | + | High | NR | NR | + | Moderate | NR | NR | ? | Moderate | NR | NR |
| SAQ 1 | + | High | + | High | NR | NR | + | High | + | Moderate | NR | NR | NR | NR |
| SAQ2 | NR | NR | NR | NR | NR | NR | NR | NR | NR | NR | ? | High | NR | NR |
| SEQ | NR | NR | NR | NR | NR | NR | NR | NR | NR | NR | + | High | NR | NR |
| SES | NR | NR | NR | NR | NR | NR | NR | NR | NR | NR | + | High | NR | NR |
| SHQ | NR | NR | NR | NR | NR | NR | NR | NR | NR | NR | + | Moderate | NR | NR |
| WSHQ | NR | NR | NR | NR | NR | NR | NR | NR | NR | NR | + | Moderate | NR | NR |
| Only neglect | | | | | | | | | | | | | | |
| MNBS | NR | NR | ± | High | + | High | NR | NR | NR | NR | ± | High | NR | NR |
| NS | + | High | + | High | NR | NR | NR | NR | NR | NR | NR | NR | NR | NR |
|  |  |  |  |  |  |  |  |  |  |  |  |  |  |  |

+sufficient rating, ? indeterminate rating, -insufficient rating, ± inconsistent rating
