# Supplementary material for: Measuring Violence Against Children: A COSMIN Systematic Review of the Psychometric and Administrative Properties of Adult Retrospective Self-report Instruments on Child Abuse and Neglect
Source: Trauma Violence Abuse. 2023 Jan 25;25(1):183–96. doi: 10.1177/15248380221145912 (PMC10666516; doi:10.1177/15248380221145912)
Supplement: sj-docx-3-tva-10.1177_15248380221145912 – Supplemental material for Measuring Violence Against Children: A COSMIN Systematic Review of the Psychometric and Administrative Properties of Adult Retrospective Self-report Instruments on Child Abuse and Neglect [file sj-docx-3-tva-10.1177_15248380221145912.docx]

Data Extraction form for phase 1: Individual studies on Content Validity

# **Part 1: Eligibility Assessment**

# Gatekeeping questions

**!! Before commencing data extraction on this study, please answer the following questions:**

1. Does the self-report tool measure actual child abuse exposure or perpetration (e.g. the Childhood Experiences of Care and Abuse scale), as opposed to measuring only *perceptions of*, *attitudes towards*, or *risk of perpetrating* child abuse (e.g. the Child Abuse Potential Inventory)?
2. Does the study fall into one of the following categories?

- A study describing the development of a new child abuse/corporal punishment measure.
- A study that includes information on the validity of a named child abuse/corporal punishment measure
- A study of an intervention that aims to prevent child abuse/corporal punishment and uses a standardised child abuse/corporal punishment measure to report pre- and post-intervention scores.
- A study that analyses the relationship between 1) scores on a validated, self-report measure of child abuse/corporal punishment and 2) scores on a validated, self-report measure of one of our correlates of interest (e.g. depression, anxiety, suicidal behaviour, substance abuse, revictimization, or violence perpetration).
- A study that analyses the relationship between 1) scores of different respondents (e.g. parent and child) on a validated self-report measure of child abuse/corporal punishment; 2) scores of the same respondents on two different validated self-report measures of child abuse/corporal punishment; or 3) scores on a self-report measure of child abuse and child abuse exposure as measured by registry data, for example from CPS records.
- A study that compares differences in child abuse exposure (as assessed using a named child abuse/corporal punishment measure) between two populations with **known differences** that are relevant to our correlates of interest (e.g. presence vs absence of suicide attempt history; presence vs absence of a clinical diagnosis of depression, anxiety, or substance use disorders, etc)

**If the answer to either of these questions is ‘no,’ then the study is ineligible for inclusion and data should not be extracted.**

# **Part 2: Main study details**

| **Table 1a. Data Extraction for Study on PROM Development** | | | | | | |
| --- | --- | --- | --- | --- | --- | --- |
| ***1a.1 Study details*** | | | | | | |
| **Author** | **Year** | | | **Full reference (APA)** | | |
|  |  | | |  | | |
| **PROM name** [version, if applicable] | | **Construct**  (e.g. child abuse history, family environment, adverse childhood experiences) | | | **Type of Abuse** (delete as appropriate) | **Language** [original language, if applicable] |
|  | |  | | |  |  |
| **Analyses included**  (delete as appropriate) | | | | **Main aims of study**  (e.g. to build a construct to be measured; to assess the content validity of an existing measure) | | |
| - *Quantitative – pilot test* - *Qualitative – concept elicitation* - *Qualitative – cognitive interview* | | | |  | | |
| Construct Specification - Detailed | | | | | | |
| **Construct to be measured**  (*Please briefly summarise the authors’ description of the construct to be measured, including its theoretical or medical origin if mentioned*)^1^ | | |  | | | |
| **Target population**  (*Please briefly summarise the author’s description of the population that the PROM is designed to be administered to*) | | |  | | | |
| **Intended context of administration**  (*Please summarise the author’s description of the context in which the measure is intended to be used, e.g. administered by a helping professional, via computer, or pencil-and-paper self-report)* | | |  | | | |

# Instructions: Identifying studies on Content Validity

| 1. Content Validity Approach (adapted from the COSMIN taxonomy)   *Please use this table to determine which data extraction tables and Risk of Bias checklists to complete.^1^* | | | | |
| --- | --- | --- | --- | --- |
| Approach + description | | Dimension | RoB checklist box | Data extraction table |
| PROM Development  A study describing the process through which the PROM was developed and tested. | | Qualitative interview study for *concept elicitation* (i.e. to identify aspects of the construct to include in the new PROM) – this includes delphi studies. | Box 1a | Table 1a |
|  |  | Cognitive Interview or other pilot study to administer the PROM to the target population and ask about the *comprehensibility* and *comprehensiveness* of the PROM | Box 1b | Table 1b |
| Content Validity  A study examining the degree to which the content of an existing PROM is an adequate reflection of all facets of the construct being measured. | | Asking respondents from the target population about relevance | Box 2a | Table 2 |
|  |  | Asking respondents from the target population about comprehensiveness | Box 2b |  |
|  |  | Asking respondents from the target population about comprehensibility | Box 2c |  |
|  |  | Asking professionals about relevance | Box 2d |  |
|  |  | Asking professionals about comprehensiveness | Box 2e |  |
| Important Definitions | | | | |
| Relevance | This refers to the extent to which items of the PROM are considered relevant to the understandings and/or individual experiences of the respondents with the construct in question.  For a PROM to be judged as ‘relevant to its target population, researchers must ask respondents about the relevance of each individual PROM instruction, item, response option, and recall period separately. | | | |
| Comprehensiveness | This refers to the extent to which all of the items of the PROM comprehensively cover the construct that the PROM intends to measure. Even if the concept elicitation phase of the PROM development study is strong, there may be additional dimensions of the construct that are salient to respondents in the cognitive interview study, pilot study, or content validity study. | | | |
| Comprehensibility | This refers to the extent to which the PROM instructions, items, response options, and recall period are understood by respondents as intended. If the comprehensibility of a PROM is poor, there is the risk that incorrect information will be gathered.  For a PROM to be judged as ‘comprehensible’ to its target population, researchers must ask respondents about the comprehensibility of each individual PROM instruction, item, response option, and recall period separately. | | | |

| For further information on evaluating the content validity of a PROM, see the *COSMIN Methodology for assessing the Content Validity of PROMS User Manual Version 1.0*, **page 11.** |
| --- |

# **Part 3: Extraction & Risk of Bias Assessment for Studies on PROM Content Validity**

Below are tailored data extraction boxes for the results of each study of the PROM’s measurement property. With reference to **table 4** above, please delete irrelevant boxes and/or duplicate relevant boxes as appropriate.

Note on Risk of Bias ratings (from User manual for Assessing Content Validity of PROMs, p. 16)

- ‘The “worst score counts” method is used in all COSMIN boxes because poor methodological aspects of a study cannot be compensated by good aspects. In defining the response options, the ‘‘worst score counts’’ method was taken into consideration. Only fatal flaws in the design or statistical analyses were regarded as inadequate quality.’

# Studies on PROM Development

| For further information on evaluating the content validity of a PROM as assessed in a PROM Development Study, see the *COSMIN Methodology for assessing the Content Validity of PROMS User Manual Version 1.0*, **page 16** onwards**.** |
| --- |

# 1a. Qualitative study on Concept Elicitation

| **Table 1a. Data Extraction for Study on PROM Development** | | | | | | | | | | | | | | | | |
| --- | --- | --- | --- | --- | --- | --- | --- | --- | --- | --- | --- | --- | --- | --- | --- | --- |
| ***1a.1 Study details*** | | | | | | | | | | | | | | | | |
| **Study orientation**  (delete as appropriate) | | | | | | | | | **Main outcomes** | | | | | | | |
| - *Quantitative* - *Qualitative* | | | | | | | | |  | | | | | | | |
| Population | | | | | | | | | | | | | | | | |
| **N** | | | **Age in years**  mean [SD, range] | | | | | **Gender**  (% female) | | | **Country** [Language] | | **Response Rate** [%]  (*How many of the individuals recruited actually participated?)* | | | |
|  | | |  | | | | |  | | |  | |  | | | |
| **Relevance of Study Population** (*Please describe the characteristics of the study population, including how well they compare to the target population)* | | | | | | |  | | | | | | | | | |
| Study Design | | | | | | | | | | | | | | | | |
| **Data collection method** (*delete as appropriate)* | | | | | **Interview schedule**  (*Please specify whether the authors used a standardised/ recognised interview guide, whether it was structured or unstructured, whether the questions were closed or open ended, and whether the interview guide was piloted before use*) | | | | | | | | | **Skill level of interviewers** (*Please briefly describe the skill-level of interviewers/group moderators. Mark as not applicable if survey method was used.)* | | |
| - Face-to-face, group (e.g. panel, focus group) - Face-to-face, individual interview - Digital or pencil-and-paper survey | | | | |  | | | | | | | | |  | | |
| **Recording strategy**  (*Please specify whether answers were recorded or transcribed verbatim. Mark as not applicable if survey method was used)* | | | | **Approach to analysis** (*Please describe the approach to coding or analysis, e.g. thematic analysis, framework analysis, content analysis)* | | | | | | **Approach to coding**  (*Please specify the coding method (e.g. axial vs open coding), whether at least some of the data was single- or dual-coded, and if dual coded, whether researchers coded data independently)^2^* | | | | | **Approach to saturation**  (*Please specify whether authors coded data until reaching saturation)* | |
|  | | | |  | | | | | |  | | | | |  | |
| Notes: | | | | | | | | | | | | | | | | |
| ^1^The construct measured by the PROM doesn’t necessarily have to be defined before the PROM is developed – it can also be defined based on the results of the PROM development study, and in some cases it can be one of the aims of the PROM development study. The important thing is that once PROM development is over, the authors have clearly described the construct that the final PROM is intended to measure. Whether it is ‘clear’ must be decided by the review team – it should be sufficient to judge whether the PROM items are *relevant* to the construct, and the construct is *comprehensively covered* by the items in the PROM | | | | | | | | | | | | | | | | |
| ***1a.2 Rating Content Validity - PROM Development Studies*** | | | | | | | | | | | | | | | | |
| **Domain** | | | | | | **Criteria for a sufficient (+) rating** | | | | | | | | | | Researcher Verdict |
| **Relevance** | | | | | | | | | | | | | | | | **+ / - / ?** |
| 1 | Are the included items relevant for the construct of interest? | | | | | The construct of interest is clearly described (i.e. ‘very good’ rating of box 1 standard 1), the origin of construct is clear (i.e. ‘very good’ rating of box 1 standard 2) and there is evidence from concept elicitation, literature, or professionals that at least 85% of the items refer to the construct of interest. | | | | | | | | | |  |
| 2 | Are the included items relevant for the target population of interest? | | | | | The target population of interest is clearly described (i.e. ‘very good’ rating box 1 standard 3) and representative respondents from the target population were involved in the elicitation of relevant items (i.e. ‘very good’ or ‘adequate’ rating box 1 standard 5) and concept elicitation (‘worst score counts’ box 1a standards 6‐13) was not inadequate.  If it is doubtful whether the study was performed in a sample representing the target population, we recommend to give an indeterminate (?) rating. | | | | | | | | | |  |
| 3 | Are the included items relevant for the context of use of interest? | | | | | The context of use of interest is clearly described (i.e. ‘very good’ rating box 1 standard 4). | | | | | | | | | |  |
| 4 | Are the response options appropriate? | | | | | A justification is provided for the response options. | | | | | | | | | |  |
| 5 | Is the recall period appropriate? | | | | | A justification is provided for the recall period. | | | | | | | | | |  |
| **Comprehensiveness** | | | | | | | | | | | | | | | | |
| 6 | Are all key concepts included? | | | | | Respondents from the target population were asked about the comprehensiveness of the PROM in  the concept elicitation phase or in a cognitive interview study that  was not inadequate (rating for quality of comprehensiveness study, i.e. box 1a standards 6‐13, or box 1b standards 26‐35) and no  key concepts were missing. | | | | | | | | | |  |
| **Comprehensibility** | | | | | | | | | | | | | | | | |
| 7 | Are the PROM instructions understood by the population of interest as intended? | | | | | Respondents were asked about the comprehensibility of the instructions (including recall period) in a cognitive interview study that was not inadequate (rating for quality of comprehensibility study, i.e. box 1b  standards 16‐25) and problems were adequately addressed. | | | | | | | | | |  |
| 8 | Are the PROM items and response options understood by the population of interest as intended? | | | | | Respondents from the target population were asked about the comprehensibility of the items and response options (including wording of the items and response  options) in a cognitive interview study that was not inadequate  (rating for quality of comprehensibility study, box 1b standards 16‐25) and problems were adequately addressed. | | | | | | | | | |  |
| ***1a.3 Overall ratings – PROM Development Study*** | | | | | | | | | | | | | | | | |
| **Domain** | | | | | | | | | | | | **Rating (+/-/?)** | | | | |
| **Relevance** (criteria 1-5, pooled^1^) | | | | | | | | | | | |  | | | | |
| **Comprehensiveness** (criteria 6) | | | | | | | | | | | |  | | | | |
| **Comprehensibility** (criteria 7-8, pooled^2^) | | | | | | | | | | | |  | | | | |
| **^1^Guide to establishing an overall relevance rating per study** | | | | | | | | | | | | | | | | |
| **+** | | At least criteria 1 and 2 are rated + AND at least two of the other three criteria on relevance are rated + Criteria 1 and 2 (relevance for construct and population) are considered the most important criteria and therefore they need to be rated +. A maximum of 1 criterion rated – is allowed, but reviewers can also rate ± in that case. | | | | | | | | | | | | | | |
| **-** | | At least criteria 1 and 2 are rated ‐ AND at least two of the other three criteria on relevance are rated ‐ | | | | | | | | | | | | | | |
| ? | | At least two of the criteria are rated ‘?’ | | | | | | | | | | | | | | |
| ± | | All other situations | | | | | | | | | | | | | | |
| **^2^Guide to establishing an overall comprehensibility rating *Per Study*** | | | | | | | | | | | | | | | | |
| **+** | | At least criterion 8 is rated + and criterion 7 is NOT rated ‐ Criterion 8 is considered the most important, but for a  sufficient rating criterion 7 should NOT be rated ‐ (it may be rated ?). | | | | | | | | | | | | | | |
| **-** | | Criterion 8 is rated – (independent of the rating for criterion 7) | | | | | | | | | | | | | | |
| ? | | Criterion 8 is rated ? (independent of the rating for criterion 7) | | | | | | | | | | | | | | |
| ± | | Criterion 8 is rated + and criterion 7 is rated ‐ | | | | | | | | | | | | | | |

| For further information on how to apply criteria 1-10 on the PROM’s relevance, comprehensiveness, and comprehensibility, see the *COSMIN Methodology for assessing the Content Validity of PROMS User Manual Version 1.0*, **page 52-59** |
| --- |

| For further information on how to develop overall assessments of PROM’s relevance, comprehensiveness, and comprehensibility, see the *COSMIN Methodology for assessing the Content Validity of PROMS User Manual Version 1.0*, **page 60-62** |
| --- |

| **Box 1a. Risk of Bias Assessment for Studies on PROM Development** | | | | | | |
| --- | --- | --- | --- | --- | --- | --- |
| *General Design Requirements* | | Very Good | Adequate | Doubtful | Inadequate | NA |
| 1. | Is a clear description provided of the construct to be measured? | Construct clearly described | Cell not relevant - do not use | Cell not relevant - do not use | Construct not clearly described | Cell not relevant - do not use |
| 2. | Is the origin of the construct clear: was a theory, conceptual framework or disease model used or clear rationale provided to define the construct to be measured? | Origin of the construct clear | Cell not relevant - do not use | Origin of the construct not clear | Cell not relevant - do not use | Cell not relevant - do not use |
| 3. | Is a clear description provided of the target population for which the PROM was developed? | Target population clearly described | Cell not relevant - do not use | Cell not relevant - do not use | Target population not clearly described | Cell not relevant - do not use |
| 4. | Is a clear description provided of the context of use? | Context of use clearly described | Cell not relevant - do not use | Context of use not clearly described | Cell not relevant - do not use | Cell not relevant - do not use |
| 5. | Was the PROM development study performed in a sample representing the target population for which the PROM was developed? | Study performed  in a sample  representing the target population | Assumable that the study was performed in a sample representing the target population, but not clearly described | Doubtful whether the study was performed in a sample representing the target population | Study not performed in a sample representing the target population (**SKIP items 6‐12**) | Cell not relevant - do not use |
| *Concept Elicitation (relevance and comprehensiveness)* | | | | | | |
| 6. | Was an appropriate qualitative data collection method used to identify relevant items for a new PROM? | Widely recognized or well justified qualitative method used, suitable for the construct and study population | Assumable that the qualitative method was appropriate and suitable for the construct and study population, but not clearly described | Only quantitative (survey) method(s) used or doubtful whether the method was suitable for the construct and study population | Method used not  appropriate or not  suitable for the construct or study population | Cell not relevant - do not use |
| 7. | Were skilled group moderators/ interviewers used? | Skilled group moderators/ interviewers used | Group moderators /interviewers had limited experience or were trained specifically for the study | Not clear if group moderators /interviewers were trained or group moderators /interviewers not trained and no experience | Cell not relevant - do not use | N/A |
| 8. | Were the group meetings or interviews based on an appropriate topic or interview guide? | Appropriate topic  or interview guide | Assumable that the topic or interview guide was appropriate, but not clearly described | Not clear if a topic guide was used or doubtful if topic or interview guide was appropriate or no guide | Cell not relevant - do not use | N/A |
| 9. | Were the group meetings or interviews recorded and transcribed verbatim? | All group meetings or interviews were recorded and transcribed verbatim | Assumable that all group meetings or interviews were recorded and transcribed verbatim, but not clearly described | Not clear if all group  Meetings of interviews were recorded and transcribed verbatim or recordings not transcribed verbatim or only notes were made during the group meetings/ interviews | No recordings and no notes | N/A |
| 10. | Was an appropriate approach used to analyse the data? | A widely recognized or well justified approach was used | Assumable that the approach was  appropriate, but not clearly described | Not clear what approach was used or doubtful whether the approach was appropriate | Approach not appropriate | Cell not relevant - do not use |
| 11. | Was at least part of the data coded independently? | At least 50% of the data was coded by  at least two researchers independently | 11‐49% of the data was coded by at least two researchers independently | Doubtful if two researchers were involved in the coding or only 1‐10% of the data was coded by at least two researchers independently | Only one researcher was involved in coding or no coding | N/A |
| 12. | Was data collection continued until saturation was reached? | Evidence provided that saturation was reached | Assumable that saturation was reached | Doubtful whether saturation was reached | Evidence suggests that saturation was not reached | N/A |
| 13. | For quantitative studies (surveys): was the sample size  appropriate? | ≥100 | 50-99 | 30-49 | <30 | N/A |
| **Final Score (based on the “worst score counts” principle)** | | | | | | |
| What is the lowest rating in standards 1-13 in box 1a? | | | |  | | |
| Any further notes on reasons for the score? | | | | | | |
|  | | | | | | |

# 1b. Cognitive Interview or Pilot study

| **Table 1b. Data Extraction for Cognitive interview or Pilot Study** | | | | | | | | | | | | | | | | | | |
| --- | --- | --- | --- | --- | --- | --- | --- | --- | --- | --- | --- | --- | --- | --- | --- | --- | --- | --- |
| ***1b.1 Study details*** | | | | | | | | | | | | | | | | | | |
| **Study orientation**  (delete as appropriate) | | | | | | | | **Main outcomes** | | | | | | | | | | |
| - *Quantitative* - *Qualitative* | | | | | | | |  | | | | | | | | | | |
| Population | | | | | | | | | | | | | | | | | | |
| **N** | | | **Age in years**  mean [SD, range] | | | | | | **Gender**  (% female) | | **Country** [Language] | | | | **Response Rate** [%]  (*How many of the individuals recruited actually participated?)* | | | |
|  | | |  | | | | | |  | |  | | | |  | | | |
| **PROM characteristics explored** (*delete as appropriate*) | | | | | | | **Were the PROM items tested in their final form?** | | | | | | **What was the minimum number of respondents that each PROM item was tested in?** *(Please specify if reported by authors)* | | | | | |
| - Comprehensibility - Comprehensiveness | | | | | | |  | | | | | |  | | | | | |
| **Relevance of Study Population** (*Please describe the characteristics of the study population, including how well they compare to the target population)* | | | | | | | | | |  | | | | | | | | |
| Design – quantitative (*Complete if applicable*) | | | | | | | | | | | | | | | | | | |
| **Response options** (please specify whether questions used multiple-choice, likert-scale, or other quantitative response mechanism) | | | | | | | | | **Approach to analysis** | | | | | | | | | |
|  | | | | | | | | |  | | | | | | | | | |
| Study Design – qualitative (*Complete if applicable)* | | | | | | | | | | | | | | | | | | |
| **Data collection method** (*delete as appropriate)* | | | | | | **Interview schedule**  (*Please specify whether the authors used a standardised/ recognised interview guide, whether it was structured or unstructured, whether the questions were closed or open ended, and whether the interview guide was piloted before use*) | | | | | | | | | | **Skill level of interviewers**  (*Please briefly describe the skill-level of interviewers/group moderators)* | | |
| - Face-to-face, group (e.g. panel, focus group) - Face-to-face, individual interview - Digital or pencil-and-paper survey with open-ended questions | | | | | |  | | | | | | | | | |  | | |
| **Recording strategy**  (*Please specify whether answers were recorded or transcribed verbatim)* | | | | **Approach to analysis** (*Please describe the approach to coding or analysis, e.g. thematic analysis, framework analysis, content analysis, and specify how many researchers participated in analysis)* | | | | | | | **Approach to coding**  (*Please specify the coding method (e.g. axial vs open coding), whether at least some of the data was single- or dual-coded, and if dual coded, whether researchers coded data independently)^2^* | | | | | | **Approach to saturation**  (*Please specify whether authors coded data until reaching saturation)* | |
|  | | | |  | | | | | | |  | | | | | |  | |
| ***1b.2 Study Findings*** | | | | | | | | | | | | | | | | | | |
| Were **respondents from the target population** asked about comprehensiveness? | | | If yes, did **respondents from the target population** rate the PROM as being comprehensive? (*Please summarise findings as described by authors)* | | | | | | | | | If the **respondents from the target population** rated the comprehensiveness of the PROM poorly, were steps taken to revise the corresponding items? *(If yes, please briefly summarise measures taken by authors)* | | | | | | |
|  | | |  | | | | | | | | |  | | | | | | |
| Were **respondents from the target population** asked about comprehensibility? | | | If yes, did **respondents from the target population** rate the PROM as being comprehensible? (*Please summarise findings as described by authors)* | | | | | | | | | If the **respondents from the target population** rated the comprehensibility of the PROM poorly, were steps taken to revise the corresponding items? *(If yes, please briefly summarise measures taken by authors)* | | | | | | |
|  | | |  | | | | | | | | |  | | | | | | |
| ***1b.2 Rating Content Validity – Cognitive Interview or Pilot Study*** | | | | | | | | | | | | | | | | | | |
| **Domain** | | | | | **Criteria for a sufficient (+) rating** | | | | | | | | | | | | | Researcher Verdict |
| **Relevance** | | | | | | | | | | | | | | | | | | **+ / - / ?** |
| 1 | Are the included items relevant for the construct of interest? | | | | The construct of interest is clearly described (i.e. ‘very good’ rating of box 1 standard 1), the origin of construct is clear (i.e. ‘very good’ rating of box 1 standard 2) and there is evidence from concept elicitation, literature, or professionals that at least 85% of the items refer to the construct of interest. | | | | | | | | | | | | |  |
| 2 | Are the included items relevant for the target population of interest? | | | | The target population of interest is clearly described (i.e. ‘very good’ rating box 1 standard 3) and representative respondents from the target population were involved in the elicitation of relevant items (i.e. ‘very good’ or ‘adequate’ rating box 1 standard 5) and concept elicitation (‘worst score counts’ box 1a standards 6‐13) was not inadequate.  If it is doubtful whether the study was performed in a sample representing the target population, we recommend to give an indeterminate (?) rating. | | | | | | | | | | | | |  |
| 3 | Are the included items relevant for the context of use of interest? | | | | The context of use of interest is clearly described (i.e. ‘very good’ rating box 1 standard 4). | | | | | | | | | | | | |  |
| 4 | Are the response options appropriate? | | | | A justification is provided for the response options. | | | | | | | | | | | | |  |
| 5 | Is the recall period appropriate? | | | | A justification is provided for the recall period. | | | | | | | | | | | | |  |
| **Comprehensiveness** | | | | | | | | | | | | | | | | | | |
| 6 | Are all key concepts included? | | | | Respondents from the target population were asked about the comprehensiveness of the PROM in  the concept elicitation phase or in a cognitive interview study that  was not inadequate (rating for quality of comprehensiveness study, i.e. box 1a standards 6‐13, or box 1b standards 26‐35) and no  key concepts were missing. | | | | | | | | | | | | |  |
| **Comprehensibility** | | | | | | | | | | | | | | | | | | |
| 7 | Are the PROM instructions understood by the population of interest as intended? | | | | Respondents from the target population were asked about the comprehensibility of the instructions (including recall period) in a cognitive interview study that was not inadequate (rating for quality of comprehensibility study, i.e. box 1b  standards 16‐25) and problems were adequately addressed. | | | | | | | | | | | | |  |
| 8 | Are the PROM items and response options understood by the population of interest as intended? | | | | Respondents from the target population were asked about the comprehensibility of the items and  response options (including wording of the items and response  options) in a cognitive interview study that was not inadequate  (rating for quality of comprehensibility study, box 1b standards 16‐25) and problems were adequately addressed. | | | | | | | | | | | | |  |
| ***1b.3 Overall ratings – PROM Development Study*** | | | | | | | | | | | | | | | | | | |
| **Domain** | | | | | | | | | | | | | | **Rating (+/-/?)** | | | | |
| **Relevance** (criteria 1-5, pooled^1^) | | | | | | | | | | | | | |  | | | | |
| **Comprehensiveness** (criteria 6) | | | | | | | | | | | | | |  | | | | |
| **Comprehensibility** (criteria 7-8, pooled^2^) | | | | | | | | | | | | | |  | | | | |
| **^1^Guide to establishing a pooled/overall relevance rating *per study*** | | | | | | | | | | | | | | | | | | |
| **+** | | At least criteria 1 and 2 are rated + AND at least two of the other three criteria on relevance are rated + Criteria 1 and 2 (relevance for construct and population) are considered the most important criteria and therefore they need to be rated +. A maximum of 1 criterion rated – is allowed, but reviewers can also rate ± in that case. | | | | | | | | | | | | | | | | |
| **-** | | At least criteria 1 and 2 are rated ‐ AND at least two of the other three criteria on relevance are rated ‐ | | | | | | | | | | | | | | | | |
| ? | | At least two of the criteria are rated ‘?’ | | | | | | | | | | | | | | | | |
| ± | | All other situations | | | | | | | | | | | | | | | | |
| **^2^Guide to establishing a pooled/overall comprehensibility rating *Per Study*** | | | | | | | | | | | | | | | | | | |
| **+** | | At least criterion 8 is rated + and criterion 7 is NOT rated ‐ Criterion 8 is considered the most important, but for a  sufficient rating criterion 7 should NOT be rated ‐ (it may be rated ?). | | | | | | | | | | | | | | | | |
| **-** | | Criterion 8 is rated – (independent of the rating for criterion 7) | | | | | | | | | | | | | | | | |
| ? | | Criterion 8 is rated ? (independent of the rating for criterion 7) | | | | | | | | | | | | | | | | |
| ± | | Criterion 8 is rated + and criterion 7 is rated ‐ | | | | | | | | | | | | | | | | |

| **1b. Risk of Bias Assessment for Cognitive Interview Study or Other Pilot Test** | | | | | | | | | |
| --- | --- | --- | --- | --- | --- | --- | --- | --- | --- |
|  | | | | Very Good | Adequate | Doubtful | | Inadequate | NA |
| 14. | Was a cognitive interview study or other pilot test conducted? | | | YES | Cell not relevant - do not use | Cell not relevant - do not use | | NO (**skip items 15-35**) | Cell not relevant - do not use |
| *General Design Requirements* | | | | | | | | | |
| 15. | Was the cognitive interview study or other pilot test performed in a sample representing the target population? | | | Study performed  in a sample  representing the  target population | Assumable that  the study was performed in a sample representing the target population, but not clearly described | Doubtful whether the study was performed in a sample representing the target population | | Study not performed in a sample representing the target population | Cell not relevant - do not use |
| *Comprehensibility* | | | | | | | | | |
| 16. | Were respondents from the target population asked about the comprehensibility of the PROM? | | | Yes | Cell not relevant - do not use | Not clear (**SKIP standards 17-25**) | | **NO** (**SKIP standards 17-25**) | Cell not relevant - do not use |
| 17. | Were all items tested in their final form? | | | All items were tested in their final form | Assumable that all items were  tested in their final form, but not clearly described | Not clear if all items  were tested in their  final form | | Items were not tested in their final  form or items were not retested after substantial adjustments | Cell not relevant - do not use |
| 18. | Was an appropriate qualitative method used to assess the comprehensibility of the PROM instructions, items, response  options, and recall period? | | | Widely recognised or well-justified qualitative method used | Assumable that  the method was appropriate but not clearly described | Only quantitative  (survey) method(s) used or doubtful whether the method was appropriate or not clear if respondents from the target population were asked about the comprehensibility of the items, response options or recall period or respondents from the target population not asked  about the comprehensibility of the PROM instructions or the recall period | | Method used not  Appropriate or respondents from the target population not asked  about the comprehensibility of the items or the response options | Cell not relevant - do not use |
| 19. | Was each item tested in an appropriate number of respondents from the target population? | | Qualitative studies | ≥7 | 4-6 | <4 or not clear | | Cell not relevant - do not use | Cell not relevant - do not use |
|  |  |  | Quantitative studies | ≥50 | ≥30 | <30 or not clear | | Cell not relevant - do not use | Cell not relevant - do not use |
| 20. | Were skilled interviewers used? | | | Skilled group moderators/ interviewers used | Group moderators /interviewers had limited experience or were trained specifically for the study | Not clear if group moderators /interviewers were  trained or group moderators /interviewers not  trained and no experience | | Cell not relevant - do not use | N/A |
| 21. | Were the interviews based on an appropriate interview guide? | | | Appropriate topic  or interview guide | Assumable that the topic or interview guide was appropriate, but not clearly described | Not clear if a topic guide was used or doubtful if topic or interview guide was appropriate or no guide | | Cell not relevant - do not use | N/A |
| 22. | Were the interviews recorded and transcribed verbatim? | | | All group meetings  or interviews were  recorded and transcribed verbatim | Assumable that  all group meetings or interviews were recorded and transcribed verbatim, but not clearly described | Not clear if all group meetings or interviews were recorded and transcribed verbatim or recordings not transcribed verbatim or only notes were made during the group  meetings/interviews | | No recordings and no notes | N/A |
| 23. | Was an appropriate approach used to analyse the data? | | | A widely  recognized or well  justified approach  was used | Assumable that the approach was appropriate, but not clearly described | Not clear what approach was used or doubtful whether the approach was appropriate | | Approach not appropriate | Cell not relevant - do not use |
| 24. | Were at least two researchers involved in the analysis? | | | At least two researchers involved in the analysis | Assumable that at least two researchers were involved in the analysis, but not clearly described | Not clear if two researchers were included in the analysis or only one researcher involved in the analysis | | Cell not relevant - do not use | Cell not relevant - do not use |
| 25. | Were problems regarding the comprehensibility of the PROM instructions, items, response options, and recall period appropriately addressed by adapting the PROM? | | | No problems found or problems appropriately addressed and PROM was adapted and retested if necessary | Assumable that there were no problems or that problems were appropriately addressed, but  not clearly described | Not clear if there were problems or doubtful if problems were appropriately addressed | | Problems not appropriately addressed or PROM was adapted but items were not re‐tested after substantial adjustments. | N/A |
| *Comprehensiveness* | | | | | | | | | |
| 26. | Were respondents from the target population asked about the comprehensiveness of the PROM? | | | Yes | Cell not relevant - do not use | Not clear (**SKIP standards 27-35**) | | **NO** (**SKIP standards 27-35**) | Cell not relevant - do not use |
| 27. | Was the final set of items tested? | | | The final set of items was tested | Assumable that the final set of items was tested, but not clearly described | Not clear if the final set of items was tested or the set of items was not re-tested after items were removed or added | | Cell not relevant - do not use | Cell not relevant - do not use |
| 28. | Was an appropriate qualitative method used to assess the comprehensiveness of the PROM? | | | Widely recognised or well-justified qualitative method used | Assumable that  the method was appropriate but not clearly described or only quantitative (survey) method(s) used | Doubtful whether the method was appropriate or method used not appropriate | | Cell not relevant - do not use | Cell not relevant - do not use |
| 29. | Was each item tested in an appropriate number of respondents from the target population? | Qualitative studies | | ≥7 | 4-6 | <4 or not clear | | Cell not relevant - do not use | Cell not relevant - do not use |
|  |  | Quantitative studies | | ≥50 | ≥30 | <30 or not clear | | Cell not relevant - do not use | Cell not relevant - do not use |
| 30. | Were skilled interviewers used? | | | Skilled interviewers used | Interviewers had limited experience or were trained specifically for the study | Not clear if interviewers were trained or interviewers not trained and no experience | | Cell not relevant - do not use | N/A |
| 31. | Were the interviews based on an appropriate interview guide? | | | Appropriate topic  or interview guide | Assumable that the topic or interview guide was appropriate, but not clearly described | Not clear if a topic guide was used or doubtful if topic or interview guide was appropriate or no guide | | Cell not relevant - do not use | N/A |
| 32. | Were the interviews recorded and transcribed verbatim? | | | All group meetings  or interviews were  recorded and transcribed verbatim | Assumable that  all group meetings or interviews were recorded and transcribed verbatim, but not clearly described | Not clear if all group meetings or interviews were recorded and transcribed verbatim or recordings not transcribed verbatim or only notes were made during the group  meetings/interviews or no recording and no notes | | Cell not relevant - do not use | N/A |
| 33. | Was an appropriate approach used to analyse the data? | | | A widely  recognized or well  justified approach  was used | Assumable that the approach was appropriate, but not clearly described | Not clear what approach was used or doubtful whether the approach was appropriate or approach not appropriate | | Cell not relevant - do not use | Cell not relevant - do not use |
| 34. | Were at least two researchers involved in the analysis? | | | At least two researchers involved in the analysis | Assumable that at least two researchers were involved in the analysis, but not clearly described | Not clear if two researchers were included in the analysis or only one researcher involved in the analysis | | Cell not relevant - do not use | Cell not relevant - do not use |
| 35. | Were problems regarding the comprehensiveness of the PROM appropriately addressed by adapting the PROM? | | | No problems found or problems  Appropriately addressed and PROM was adapted and retested if necessary | Assumable that  there were no problems or that problems  were appropriately addressed, but  not clearly described | Not clear if there were problems or doubtful if problems were appropriately addressed or PROM was adapted but items were not retested after substantial adjustments | | Problems not appropriately addressed | N/A |
| **Final Score (based on the “worst score counts” principle)** | | | | | | | | | |
| What is the lowest rating of any of the standards in box 1b? | | | | | | |  | | |
| Any further notes on reasons for the score? | | | | | | | | | |
|  | | | | | | | | | |

| For further information on evaluating the Risk of Bias in a PROM Development Study, see the *COSMIN Methodology for assessing the Content Validity of PROMS User Manual Version 1.0*, **page 16** onwards**.** |
| --- |

# Studies on Content Validity

| For further information on evaluating the content validity of a PROM as assessed in a Content Validity Study, see the *COSMIN Methodology for assessing the Content Validity of PROMS User Manual Version 1.0*, **page 36** onwards**.** |
| --- |

| **Table 2. Data Extraction for Study on Content Validity** | | | | | | | | | | | | | | | | | | | |
| --- | --- | --- | --- | --- | --- | --- | --- | --- | --- | --- | --- | --- | --- | --- | --- | --- | --- | --- | --- |
| - 1. ***Study details*** | | | | | | | | | | | | | | | | | | | |
| **Study orientation**  (delete as appropriate) | | | | | | | | | | **Main outcomes** | | | | | | | | | |
| - *Quantitative* - *Qualitative* | | | | | | | | | |  | | | | | | | | | |
| Population | | | | | | | | | | | | | | | | | | | |
| **N** | | | | **Age in years**  mean [SD, range] | | | | | **Gender**  (% female) | | | **Country** [Language] | | | **Response Rate** [%]  (*How many of the individuals recruited actually participated?)* | | | | |
|  | | | |  | | | | |  | | |  | | |  | | | | |
| **Study population** (*delete as appropriate)* | | | | | | | | | | | | **Relevance of Study Population** (*Please describe the characteristics of the study population, including how well they compare to the target population)* | | | | | | | |
| - Professionals (*e.g. social workers, researchers with expertise in child/family welfare*) - General population (*e.g. community sample, not selected specifically for exposure to child abuse or neglect*) - Targeted population (*i.e. sample of respondents who are likelier to be exposed to child abuse or neglect, e.g. psychiatric inpatients, foster children, or people with depression*) | | | | | | | | | | | |  | | | | | | | |
| **PROM characteristics explored** (*delete as appropriate*) | | | | | | | | **What was the minimum number of respondents that each PROM item was tested in?** *(Please specify if reported by authors)* | | | | | | | | | | | |
| - Relevance - Comprehensibility - Comprehensiveness | | | | | | | |  | | | | | | | | | | | |
| Design – quantitative (*Complete if applicable*) | | | | | | | | | | | | | | | | | | | |
| **Response options** (*please specify whether questions used multiple-choice, likert-scale, or other quantitative response mechanism*) | | | | | | | | | | | | | **Approach to analysis** | | | | | | |
|  | | | | | | | | | | | | |  | | | | | | |
| Study Design – qualitative (*Complete if applicable)* | | | | | | | | | | | | | | | | | | | |
| **Data collection method** (*delete as appropriate)* | | | | | | **Interview schedule**  (*Please specify whether the authors used a standardised/ recognised interview guide, whether it was structured or unstructured, whether the questions were closed or open ended, and whether the interview guide was piloted before use*) | | | | | | | | | | | **Skill level of interviewers** (*Please briefly describe the skill-level of interviewers/group moderators. Mark as not applicable if survey method was used.)* | | |
| - Face-to-face, group (e.g. panel, focus group) - Face-to-face, individual interview - Digital or pencil-and-paper survey | | | | | |  | | | | | | | | | | |  | | |
| **Recording strategy**  (*Please specify whether answers were recorded or transcribed verbatim. Mark as not applicable if survey method was used)* | | | | | **Approach to analysis** (*Please describe the approach to coding or analysis, e.g. thematic analysis, framework analysis, content analysis)* | | | | | | **Approach to coding**  (*Please specify the coding method (e.g. axial vs open coding), whether at least some of the data was single- or dual-coded, and if dual coded, whether researchers coded data independently)^2^* | | | | | | | | **Approach to saturation**  (*Please specify whether authors coded data until reaching saturation)* |
|  | | | | |  | | | | | |  | | | | | | | |  |
| ***2.2 Study Findings*** | | | | | | | | | | | | | | | | | | | |
| Were **respondents from the target population** asked about relevance? | | | If yes, did **respondents from the target population** rate the PROM as being relevant?  *(Please summarise findings as described by authors)* | | | | | | | | | | | If the **respondents from the target population** rated the relevance of the PROM poorly, were the corresponding PROM items revised?  *(If yes, please briefly summarise measures taken by authors)* | | | | | |
|  | | |  | | | | | | | | | | |  | | | | | |
| Were **respondents from the target** **population** asked about comprehensiveness? | | | If yes, did **respondents from the target population** rate the PROM as being comprehensive? (*Please summarise findings as described by authors)* | | | | | | | | | | | If the **respondents from the target population** rated the comprehensiveness of the PROM poorly, were steps taken to revise the corresponding items? *(If yes, please briefly summarise measures taken by authors)* | | | | | |
|  | | |  | | | | | | | | | | |  | | | | | |
| Were **respondents from the target population** asked about comprehensibility? | | | If yes, did **respondents from the target population** rate the PROM as being comprehensible? (*Please summarise findings as described by authors)* | | | | | | | | | | | If the **respondents from the target population** rated the comprehensibility of the PROM poorly, were steps taken to revise the corresponding items? *(If yes, please briefly summarise measures taken by authors)* | | | | | |
|  | | |  | | | | | | | | | | |  | | | | | |
| Were **professionals** asked about relevance? | | | If yes, did **professionals** rate the PROM as being relevant?  *(Please summarise findings as described by authors)* | | | | | | | | | | | If the **professionals** rated the relevance of the PROM poorly, were the corresponding PROM items revised?  *(If yes, please briefly summarise measures taken by authors)* | | | | | |
|  | | |  | | | | | | | | | | |  | | | | | |
| Were p**rofessionals** asked about comprehensiveness? | | | If yes, did **Professionals** rate the PROM as being comprehensive? (*Please summarise findings as described by authors)* | | | | | | | | | | | If the **professionals** rated the comprehensiveness of the PROM poorly, were steps taken to revise the corresponding items? *(If yes, please briefly summarise measures taken by authors)* | | | | | |
|  | | |  | | | | | | | | | | |  | | | | | |
| ***2.3 Rating Content Validity – Content Validity Studies*** | | | | | | | | | | | | | | | | | | | |
| **Domain** | | | | | | | **Criteria for a sufficient (+) rating** | | | | | | | | | | | Verdict (study 1) | |
| **Relevance** | | | | | | | | | | | | | | | | | | **+ / - / ?** | |
| 1 | Are the included items relevant for the construct of interest? | | | | | | Professionals rated the relevance of the items for the construct of  interest in a content validity study that was not inadequate (i.e. ‘very good’, ‘adequate’ or ‘doubtful’ rating for quality of relevance  study in box 2d, standards 22‐26) and found at least 85% of the items  relevant for the construct. | | | | | | | | | | |  | |
| 2 | Are the included items relevant for the target population of interest? | | | | | | Respondents from the target population rated the relevance of the items for them in a content validity study that was not inadequate (rating for quality of relevance study, box 2a standards 1‐7) and found at least 85% of the items relevant for them. | | | | | | | | | | |  | |
| 3 | Are the included items relevant for the context of use of interest? | | | | | | Professionals rated the relevance of the items for the context of use  of interest in a content validity study that was not inadequate  (rating for quality of relevance study, i.e. box 2d, standards 22‐26)  and found at least 85% of the items relevant for the context of use. | | | | | | | | | | |  | |
| 4 | Are the response options appropriate? | | | | | | Respondents from the target population or professionals rated the appropriateness of the response options in a content validity study that was not inadequate (rating for quality of relevance study, i.e. box 2a standards 1‐7 or box 2d standards 22‐26) and found at least 85% of the response options relevant. | | | | | | | | | | |  | |
| 5 | Is the recall period appropriate? | | | | | | Respondents from the target population or professionals rated the appropriateness of the recall period in a content validity study that was not inadequate (rating for quality of relevance study, i.e. box 2a standards 1‐7 or box 2d standards 22‐26) and found the recall period appropriate. | | | | | | | | | | |  | |
| **Comprehensiveness** | | | | | | | | | | | | | | | | | | | |
| 6 | Are all key concepts included? | | | | | | Respondents from the target population or professionals were asked about the comprehensiveness of the PROM in a content validity study that was not inadequate (rating for quality of comprehensiveness study, i.e. box 2b standards 8‐14, or box 2e standards 27‐31) and no key concepts were missing. | | | | | | | | | | |  | |
| **Comprehensibility** | | | | | | | | | | | | | | | | | | | |
| 7 | Are the PROM instructions understood by the population of interest as intended? | | | | | | Respondents from the target population were asked about the comprehensibility of the instructions (including recall period) in a content validity study that was not inadequate (rating for quality of comprehensibility study, box 2c standards 15‐21) and no important problems were found. | | | | | | | | | | |  | |
| 8 | Are the PROM items and response options understood by the population of interest as intended? | | | | | | Respondents from the target population were asked about the comprehensibility of the items and  response options in a content validity study that was not inadequate (rating for quality of comprehensibility study, box 2c standards 15‐21) and no important problems were found for at least 85% of the items and response options. | | | | | | | | | | |  | |
| ***2.4 Overall ratings – PROM Development Study*** | | | | | | | | | | | | | | | | | | | |
| **Domain** | | | | | | | | | | | | | | | | **Rating (+/-/?)** | | | |
| **Relevance** (criteria 1-5, pooled^1^) | | | | | | | | | | | | | | | |  | | | |
| **Comprehensiveness** (criteria 6) | | | | | | | | | | | | | | | |  | | | |
| **Comprehensibility** (criteria 7-8, pooled^2^) | | | | | | | | | | | | | | | |  | | | |
| **^1^Guide to establishing a pooled/overall relevance rating *per study*** | | | | | | | | | | | | | | | | | | | |
| **+** | | At least criteria 1 and 2 are rated + AND at least two of the other three criteria on relevance are rated + Criteria 1 and 2 (relevance for construct and population) are considered the most important criteria and therefore they need to be rated +. A maximum of 1 criterion rated – is allowed, but reviewers can also rate ± in that case. | | | | | | | | | | | | | | | | | |
| **-** | | At least criteria 1 and 2 are rated ‐ AND at least two of the other three criteria on relevance are rated ‐ | | | | | | | | | | | | | | | | | |
| ? | | At least two of the criteria are rated ‘?’ | | | | | | | | | | | | | | | | | |
| ± | | All other situations | | | | | | | | | | | | | | | | | |
| **^2^Guide to establishing a pooled/overall comprehensibility rating p*er study*** | | | | | | | | | | | | | | | | | | | |
| **+** | | At least criterion 8 is rated + and criterion 7 is NOT rated ‐ Criterion 8 is considered the most important, but for a  sufficient rating criterion 7 should NOT be rated ‐ (it may be rated ?). | | | | | | | | | | | | | | | | | |
| **-** | | Criterion 8 is rated – (independent of the rating for criterion 7) | | | | | | | | | | | | | | | | | |
| ? | | Criterion 8 is rated ? (independent of the rating for criterion 7) | | | | | | | | | | | | | | | | | |
| ± | | Criterion 8 is rated + and criterion 7 is rated ‐ | | | | | | | | | | | | | | | | | |

| For further information on how to apply criteria 1-10 on the PROM’s relevance, comprehensiveness, and comprehensibility, see the *COSMIN Methodology for assessing the Content Validity of PROMS User Manual Version 1.0*, **page 52-59** |
| --- |

| For further information on how to develop *overall* assessments of PROM’s relevance, comprehensiveness, and comprehensibility, see the *COSMIN Methodology for assessing the Content Validity of PROMS User Manual Version 1.0*, **page 60-62** |
| --- |

| **Box 2. Risk of Bias Assessment for Studies on Content Validity** | | | | | | | | |
| --- | --- | --- | --- | --- | --- | --- | --- | --- |
| **2a. Asking target population about Relevance** | | | | | | | | |
| *Design Requirements* | | | Very Good | Adequate | Doubtful | | Inadequate | NA |
| 1. | Was an appropriate method used to ask respondents from the target population whether each item is relevant for their experience with the condition? | | Widely recognised or well-justified qualitative method used | Only quantitative (survey) method(s) used or assumable that the method was appropriate but not clearly described | Not clear if respondents from the target population were asked whether each item is relevant or doubtful whether the method was appropriate | | Not clear if respondents from the target population were asked whether each item is relevant or doubtful whether the method was appropriate | Cell not relevant - do not use |
| 2. | Was each item tested in an appropriate number of respondents from the target population? | Qualitative studies | ≥7 | 4-6 | <4 or not clear | | Cell not relevant - do not use | Cell not relevant - do not use |
|  |  | Quantitative studies | ≥50 | ≥30 | <30 or not clear | | Cell not relevant - do not use | Cell not relevant - do not use |
| 3. | Were skilled group moderators or interviewers used? | | Skilled group moderators/ interviewers used | Group moderators /interviewers had limited experience or were trained specifically for the study | Not clear if group moderators /interviewers were  trained or group moderators /interviewers not  trained and no experience | | Cell not relevant - do not use | N/A |
| 4. | Were the interviews based on an appropriate interview guide? | | Appropriate topic  or interview guide | Assumable that the topic or interview guide was appropriate, but not clearly described | Not clear if a topic guide was used or doubtful if topic or interview guide was appropriate or no guide | | Cell not relevant - do not use | N/A |
| 5. | Were the group meetings or interviews recorded and transcribed verbatim? | | All group meetings  or interviews were  recorded and transcribed verbatim | Assumable that  all group meetings or interviews were recorded and transcribed verbatim, but not clearly described | Not clear if all group meetings or interviews were recorded and transcribed verbatim or recordings not transcribed verbatim or only notes were made during the group meetings/ interviews | | no recording and no notes | N/A |
| *Analyses* | | | | | | | | |
| 6. | Was an appropriate approach used to analyse the data? | | A widely  recognized or well  justified approach  was used | Assumable that the approach was appropriate, but not clearly described | Not clear what approach was used or doubtful whether the approach was appropriate | | approach not appropriate | Cell not relevant - do not use |
| 7. | Were at least two researchers involved in the analysis? | | At least two researchers involved in the analysis | Assumable that at least two researchers were involved in the analysis, but not clearly described | Not clear if two researchers were included in the analysis or only one researcher involved in the analysis | | Cell not relevant - do not use | Cell not relevant - do not use |
| **Final Score (based on the “worst score counts” principle)** | | | | | | | | |
| What is the lowest rating of any of the standards in box 2a? | | | | | |  | | |
| Any further notes on reasons for the score? | | | | | | | | |
|  | | | | | | | | |

| **2b. Asking target population about comprehensiveness** | | | | | | | | |
| --- | --- | --- | --- | --- | --- | --- | --- | --- |
| *Design Requirements* | | | Very Good | Adequate | Doubtful | | Inadequate | NA |
| 8. | Was an appropriate method used for assessing the comprehensiveness of the PROM? | | Widely recognised or well-justified qualitative method used | Only quantitative (survey) method(s) used or assumable that the method was appropriate but not clearly described | Doubtful whether the method was appropriate | | Method used not appropriate | Cell not relevant - do not use |
| 9. | Was each item tested in an appropriate number of respondents from the target population? | Qualitative studies | ≥7 | 4-6 | <4 or not clear | | Cell not relevant - do not use | Cell not relevant - do not use |
|  |  | Quantitative studies | ≥50 | ≥30 | <30 or not clear | | Cell not relevant - do not use | Cell not relevant - do not use |
| 10. | Were skilled group moderators or interviewers used? | | Skilled group moderators/ interviewers used | Group moderators /interviewers had limited experience or were trained specifically for the study | Not clear if group moderators /interviewers were  trained or group moderators /interviewers not  trained and no experience | | Cell not relevant - do not use | N/A |
| 11. | Were the group meetings or interviews based on an appropriate interview guide? | | Appropriate topic  or interview guide | Assumable that the topic or interview guide was appropriate, but not clearly described | Not clear if a topic guide was used or doubtful if topic or interview guide was appropriate or no guide | | Cell not relevant - do not use | N/A |
| 12. | Were the group meetings or interviews recorded and transcribed verbatim? | | All group meetings  or interviews were  recorded and transcribed verbatim | Assumable that  all group meetings or interviews were recorded and transcribed verbatim, but not clearly described | Not clear if all group meetings or interviews were recorded and transcribed verbatim or recordings not transcribed verbatim or only notes were made during the group meetings/ interviews | | no recording and no notes | N/A |
| *Analyses* | | | | | | | | |
| 13. | Was an appropriate approach used to analyse the data? | | A widely  recognized or well  justified approach  was used | Assumable that the approach was appropriate, but not clearly described | Not clear what approach was used or doubtful whether the approach was appropriate | | approach not appropriate | Cell not relevant - do not use |
| 14. | Were at least two researchers involved in the analysis? | | At least two researchers involved in the analysis | Assumable that at least two researchers were involved in the analysis, but not clearly described | Not clear if two researchers were included in the analysis or only one researcher involved in the analysis | | Cell not relevant - do not use | Cell not relevant - do not use |
| **Final Score (based on the “worst score counts” principle)** | | | | | | | | |
| What is the lowest rating of any of the standards in box 2b? | | | | | |  | | |
| Any further notes on reasons for the score? | | | | | | | | |
|  | | | | | | | | |

| **2c. Asking target population about comprehensibility** | | | | | | | | |
| --- | --- | --- | --- | --- | --- | --- | --- | --- |
| *Design Requirements* | | | Very Good | Adequate | Doubtful | | Inadequate | NA |
| 15. | Was an appropriate qualitative method used for assessing the comprehensibility of the PROM instructions, items, response options, and recall period? | | Widely recognized or well justified qualitative method used | Assumable that the method was appropriate but not clearly described | Only quantitative (survey) method(s) used or doubtful whether the method was appropriate or not clear if respondents from the target population were asked about the comprehensibility of the items, response options or recall period or respondents from the target population not asked about the comprehensibility of the PROM instructions | | Method used not appropriate of respondents from the target population not asked about the comprehensibility of the items, response options, or recall period | Cell not relevant - do not use |
| 16. | Was each item tested in an appropriate number of respondents from the target population? | Qualitative studies | ≥7 | 4-6 | <4 or not clear | | Cell not relevant - do not use | Cell not relevant - do not use |
|  |  | Quantitative studies | ≥50 | ≥30 | <30 or not clear | | Cell not relevant - do not use |  |
| 17. | Were skilled group moderators or interviewers used? | | Skilled group moderators/ interviewers used | Group moderators /interviewers had limited experience or were trained specifically for the study | Not clear if group moderators /interviewers were  trained or group moderators /interviewers not  trained and no experience | | Cell not relevant - do not use | Cell not relevant - do not use |
| 18. | Were the group meetings or interviews based on an appropriate interview guide? | | Appropriate topic  or interview guide | Assumable that the topic or interview guide was appropriate, but not clearly described | Not clear if a topic guide was used or doubtful if topic or interview guide was appropriate or no guide | | Cell not relevant - do not use | N/A |
| 19. | Were the group meetings or interviews recorded and transcribed verbatim? | | All group meetings  or interviews were  recorded and transcribed verbatim | Assumable that  all group meetings or interviews were recorded and transcribed verbatim, but not clearly described | Not clear if all group meetings or interviews were recorded and transcribed verbatim or recordings not transcribed verbatim or only notes were made during the group meetings/ interviews | | no recording and no notes | N/A |
| *Analyses* | | | | | | | | |
| 20. | Was an appropriate approach used to analyse the data? | | A widely  recognized or well  justified approach  was used | Assumable that the approach was appropriate, but not clearly described | Not clear what approach was used or doubtful whether the approach was appropriate | | approach not appropriate | Cell not relevant - do not use |
| 21. | Were at least two researchers involved in the analysis? | | At least two researchers involved in the analysis | Assumable that at least two researchers were involved in the analysis, but not clearly described | Not clear if two researchers were included in the analysis or only one researcher involved in the analysis | | Cell not relevant - do not use | Cell not relevant - do not use |
| **Final Score (based on the “worst score counts” principle)** | | | | | | | | |
| What is the lowest rating of any of the standards in box 2c? | | | | | |  | | |
| Any further notes on reasons for the score? | | | | | | | | |
|  | | | | | | | | |

| **2d. Asking Professionals about relevance** | | | | | | | | |
| --- | --- | --- | --- | --- | --- | --- | --- | --- |
| *Design Requirements* | | | Very Good | Adequate | Doubtful | | Inadequate | NA |
| 22. | Was an appropriate method used to ask professionals whether each item is relevant for the construct of interest? | | Widely recognized or well justified qualitative method used | Only quantitative (survey) method(s) used or assumable that the method was appropriate but not clearly described | Not clear if professionals were asked about the comprehensibility of the items, response options or recall period or respondents from the target population not asked about the comprehensibility of the PROM instructions | | Method used not appropriate of respondents from the target population not asked about the comprehensibility of the items, response options, or recall period | Cell not relevant - do not use |
| 23. | Were professionals from all relevant disciplines included? | | Professionals from all required disciplines were included | Assumable that professionals from all required disciplines were included, but not clearly described | Doubtful whether professionals from all required disciplines were included or relevant professionals were not included | | Cell not relevant - do not use | Cell not relevant - do not use |
| 24. | Was each item tested in an appropriate number of Professionals? | Qualitative studies | ≥7 | 4-6 | <4 or not clear | | Cell not relevant - do not use | Cell not relevant - do not use |
|  |  | Quantitative studies | ≥50 | ≥30 | <30 or not clear | | Cell not relevant - do not use | Cell not relevant - do not use |
| *Analyses* | | | | | | | | |
| 25. | Was an appropriate approach used to analyse the data? | | A widely  recognized or well  justified approach  was used | Assumable that the approach was appropriate, but not clearly described | Not clear what approach was used or doubtful whether the approach was appropriate | | approach not appropriate | Cell not relevant - do not use |
| 26. | Were at least two researchers involved in the analysis? | | At least two researchers involved in the analysis | Assumable that at least two researchers were involved in the analysis, but not clearly described | Not clear if two researchers were included in the analysis or only one researcher involved in the analysis | | Cell not relevant - do not use | Cell not relevant - do not use |
| **Final Score (based on the “worst score counts” principle)** | | | | | | | | |
| What is the lowest rating of any of the standards in box 2d? | | | | | |  | | |
| Any further notes on reasons for the score? | | | | | | | | |
|  | | | | | | | | |

| **2e. Asking about Comprehensiveness** | | | | | | | | |
| --- | --- | --- | --- | --- | --- | --- | --- | --- |
| *Design Requirements* | | | Very Good | Adequate | Doubtful | | Inadequate | NA |
| 27. | Was an appropriate method used for assessing the comprehensiveness of the PROM? | | Widely recognized or well justified method used | Only quantitative (survey) method(s) used or assumable that the method was appropriate but not clearly described | Doubtful whether the method was appropriate | | Method used not appropriate | Cell not relevant - do not use |
| 28. | Were professionals from all relevant disciplines included? | | Professionals from all required disciplines were included | Assumable that professionals from all required disciplines were included, but not clearly described | Doubtful whether professionals from all required disciplines were included or relevant professionals were not included | | Cell not relevant - do not use | Cell not relevant - do not use |
| 29. | Was each item tested in an appropriate number of Professionals? | Qualitative studies | ≥7 | 4-6 | <4 or not clear | | Cell not relevant - do not use | Cell not relevant - do not use |
|  |  | Quantitative studies | ≥50 | ≥30 | <30 or not clear | | Cell not relevant - do not use | Cell not relevant - do not use |
| *Analyses* | | | | | | | | |
| 30. | Was an appropriate approach used to analyse the data? | | A widely  recognized or well  justified approach  was used | Assumable that the approach was appropriate, but not clearly described | Not clear what approach was used or doubtful whether the approach was appropriate | | approach not appropriate | Cell not relevant - do not use |
| 31. | Were at least two researchers involved in the analysis? | | At least two researchers involved in the analysis | Assumable that at least two researchers were involved in the analysis, but not clearly described | Not clear if two researchers were included in the analysis or only one researcher involved in the analysis | | Cell not relevant - do not use | Cell not relevant - do not use |
| **Final Score (based on the “worst score counts” principle)** | | | | | | | | |
| What is the lowest rating of any of the standards in box 2e? | | | | | |  | | |
| Any further notes on reasons for the score? | | | | | | | | |
|  | | | | | | | | |

| For further information on evaluating the Risk of Bias of a Content Validity Study, see the *COSMIN Methodology for assessing the Content Validity of PROMS User Manual Version 1.0*, **page 36** onwards**.** |
| --- |

# 3. Overall Content validity summary

| ***3.1 Overall scores - PROM Development Study*** | | |
| --- | --- | --- |
| **Domain** | **Content Validity Rating**  **(+/-/?)** | **Evidence Quality Rating**  **(+/-/?)** |
| **Relevance** (criteria 1-5, pooled) |  |  |
| **Comprehensiveness** (criteria 6) |  |  |
| **Comprehensibility** (criteria 7-8, pooled) |  |  |

| ***3.2 Overall scores – Content Validity Study*** | | |
| --- | --- | --- |
| **Domain** | **Content Validity Rating**  **(+/-/?)** | **Evidence Quality Rating**  **(+/-/?)** |
| **Relevance** (criteria 1-5, pooled) |  |  |
| **Comprehensiveness** (criteria 6) |  |  |
| **Comprehensibility** (criteria 7-8, pooled) |  |  |
