# Supplementary material for: Measuring Violence Against Children: A COSMIN Systematic Review of the Psychometric and Administrative Properties of Adult Retrospective Self-report Instruments on Child Abuse and Neglect
Source: Trauma Violence Abuse. 2023 Jan 25;25(1):183–96. doi: 10.1177/15248380221145912 (PMC10666516; doi:10.1177/15248380221145912)
Supplement: sj-docx-4-tva-10.1177_15248380221145912 – Supplemental material for Measuring Violence Against Children: A COSMIN Systematic Review of the Psychometric and Administrative Properties of Adult Retrospective Self-report Instruments on Child Abuse and Neglect [file sj-docx-4-tva-10.1177_15248380221145912.docx]

Data Extraction form for phase 1: Individual studies on Other Property Measures of Patient-Reported Outcome Measures (PROMs)

# Part 1: Gatekeeping Questions

| **!! Before commencing data extraction on this study, please answer the following questions:**   1. Does the self-report tool measure actual child abuse exposure or perpetration (e.g. the Childhood Experiences of Care and Abuse scale), as opposed to measuring only *perceptions of*, *attitudes towards*, or *risk of perpetrating* child abuse (e.g. the Child Abuse Potential Inventory)? 2. Does the study fall into one of the following categories?  - A study that includes some psychometrics of a named child abuse/corporal punishment measure. - A study describing the development of a new child abuse/corporal punishment measure. - A study of an intervention that aims to prevent child abuse/corporal punishment and uses a standardised child abuse/corporal punishment measure to report pre- and post-intervention scores. - A study that analyses the relationship between 1) scores on a validated, self-report measure of child abuse/corporal punishment and 2) scores on a validated, self-report measure of one of our correlates of interest (e.g. depression, anxiety, suicidal behaviour, substance abuse, revictimization, or violence perpetration). - A study that analyses the relationship between 1) scores of different respondents (e.g. parent and child) on a validated self-report measure of child abuse/corporal punishment; 2) scores of the same respondents on two different validated self-report measures of child abuse/corporal punishment; or 3) scores on a self-report measure of child abuse and child abuse exposure as measured by registry data, for example from CPS records. - A study that compares differences in child abuse exposure (as assessed using a named child abuse/corporal punishment measure) between two populations with **known differences** that are relevant to our correlates of interest (e.g. presence vs absence of suicide attempt history; presence vs absence of a clinical diagnosis of depression, anxiety, or substance use disorders, etc)   **If the answer to either of these questions is ‘no,’ then the study is ineligible for inclusion and data should not be extracted.** |
| --- |

# Part 2. Overview of article details

| 1. Study details | | | | | |
| --- | --- | --- | --- | --- | --- |
| **Author** | **Year** | | **Full reference (APA)** | | |
|  |  | |  | | |
| **PROM name** [version, if applicable] | | **Construct**  (e.g. child abuse history, family environment, adverse childhood experiences) | | **Type of Abuse** (delete as appropriate) | **Language** [original language, if applicable] |
|  | |  | |  |  |
| **Study orientation**  (delete as appropriate) | | | **Main outcomes**  e.g. ***correlational*** (depression, suicidal ideation, anxiety) and/or ***psychometric*** (internal consistency, inter-rater reliability, etc) outcomes | | |
| - *Correlates of child abuse* - *Psychometrics of Measure* - *Child abuse prevention intervention* | | |  | | |

| 1. Study Characteristics *(if any of these details are not immediately available, enter ‘****NR****’ (‘not reported’) into the relevant cell)* | | | | | | | | | | | |
| --- | --- | --- | --- | --- | --- | --- | --- | --- | --- | --- | --- |
| Population | | | | | | | | | | | |
| **Recruitment criteria / population characteristics** | **N** | **Age in years**  mean [SD, range] | | | **Gender**  % female | | | **Setting** | | **Country (Languages)** | **Response Rate** (%) |
|  |  |  | | |  | | |  | |  |  |
| Design | | | | | | | | | | | |
| **Study type**  (delete as appropriate) | | **Study design**  (e.g. RCT, case-control, cohort, cross-sectional) | | | | | **Year of data collection** | | | | **Number of data collection points [frequency]** |
| - Interventional - Observational - Psychometric | |  | | | | |  | | | |  |
| PROM Scores in sample | | | | | | | | | | | |
| Subscale | | N | Mean | | | SD | | | Lowest score | | Highest score |
| Total | |  |  | | |  | | |  | |  |
| Subscale 1 | |  |  | | |  | | |  | |  |
| Subscale 2… (*duplicate rows below if needed)* | |  |  | | |  | | |  | |  |
| Source of data on sample PROM scores (page number + e.g. table or figure number ) | | | |  | | | | | | | |

# Part 3: Identifying Measurement Properties Studied

| 1. Measurement Properties evaluated in the article (adapted from the COSMIN taxonomy)   *Please use this table to determine which data extraction tables and Risk of Bias checklists to complete.^1^* | | | | |
| --- | --- | --- | --- | --- |
| Enter ‘X’ if present | Measurement Property | Description | Corresponding RoB checklist box | Corresponding data extraction table |
| *Internal Structure* | | | | |
|  | Structural Validity  *See COSMIN User Manual for Systematic Reviews p. 47* | A study examining the degree to which the scores of a PROM are an adequate reflection of the *dimensionality* of the construct to be measured.   - Structural validity is only relevant for instruments that are based on a reflective model (in which all items are manifestations of the same construct) rather than a formative model (in which the items each manifest separate components of a construct, and can only fully manifest the construct when taken together).^2^   **Suitable analyses:**   - Classical Test Theory (CTT) such as Confirmatory Factor Analysis, Exploratory Factor Analysis, or Principal Component Analysis - Item Response Theory (IRT) such as Rasch or Mokken Scale Analysis | Box 3 | Table 3 |
|  | Internal Consistency  *See COSMIN User Manual for Systematic Reviews p. 50* | A study of the degree of *interrelatedness* among the PROM’s items. Studies usually compare one of the PROM’s (sub)scales against another of the (sub)scales to determine whether they correlate, and may assess the *unidimensionality* of each subscale if appropriate.  **Suitable analyses:**  Internal Consistency Coefficient, calculated for each subscale   - *For continuous scores:* Cronbach’s alphas or omegas - *For dichotomous scores:* Cronbach’s alphas for each unidimensional scale or subscale - *For Item Response Theory (IRT)-based scores:* Standard Error of the Theta (SE (θ)) or reliability coefficient | Box 4 | Table 4 |
|  | Cross-Cultural Validity/ Measurement Invariance  *See COSMIN User Manual for Systematic Reviews p. 51* | A study that assesses the degree to which the performance of the items on a translated or culturally adapted PROM are an adequate reflection of the performance of the items of the original version of the PROM  **OR**  A study that determines whether the PROM is measurement invariant by looking for meaningful differences in scores between (sub)groups who have the same key level of exposure to the key indicator (in our case child abuse/neglect), but differ according to e.g. age, gender, language, ethnicity or geographic location.  **Suitable analyses could include:**   - Classical test theory - Multi-Group factor analysis - Ordinal regression models - Item response theory - Assessments of Differential Item Functioning (DIF), including a McFadden’s R measure | Box 5 | Table 5 |
| ***Remaining Measurement Properties*** | | | | |
|  | Reliability  *See COSMIN User Manual for Systematic Reviews p. 53* | Reliability refers to the proportion of total variance in the measurements which is due to ‘true’ differences in respondents from the target population.  A study on a PROM’s reliability examines whether the scores generated by a PROM remain the same when repeated under several different conditions, such as:   - When using different subscales/sets of items from the same PROM (*internal consistency*)^3^ - When administered at different time-points (*test-retest reliability*) - When administered by different persons on the same occasion (*inter-rater reliability*) - When administered by the same person on different occasions (*intra-rater reliability*)   **Suitable analyses could include:^4^**   - Intraclass Correlation Coefficient (for continuous scores) - Kappa (for dichotomous or nominal scores) - Weighted Kappa (for ordinal scores) | Box 6 | Table 6 |
|  | Measurement Error  *See COSMIN User Manual for Systematic Reviews p. 56* | Studies that examine the systematic and/or random error of an individual patient’s score that is not attributable to true changes in the construct to be measured.  **Suitable analyses include:^5^**   - Classic Test Theory & Standard Error of Measurement (SEM) based on a test-retest design. - Percentage (%) of agreement (positive and negative) - Comparisons of the Smallest Detectable Change (SDC) with the Minimal Important Change (MIC) - Comparisons of the Limits of Agreement (LoA) with the Minimal Important Change (MIC) | Box 7 | Table 7 |
|  | Criterion Validity  *See COSMIN User Manual for Systematic Reviews, p. 57* | The degree to which the scores of the PROM align with the scores of a pre-specified ‘gold standard’ PROM. **PLEASE NOTE: There is no recognised gold standard child abuse measure, so for the purposes of our review, this box should only be used in instances where a shortened or adapted version of a PROM is compared to the original version.**  **Suitable analyses:**   - Correlation (r) with a ‘gold standard’ comparator measure - Area Under the Curve (AUC) | Box 8 | Table 8 |
|  | Hypotheses Testing for construct validity  *See COSMIN User Manual for Systematic Reviews, p. 58* | Studies assessing the degree to which a patient’s PROM scores are consistent with specific hypotheses, for example about the relationship of the PROM score to scores of other outcome measures, or about differences in PROM scores between relevant groups.  This can include assessments of the degree to which the scores of the PROM align with the scores of an outcome assessed either at the same time (*concurrent validity*) or at a later time (*predictive validity*).  **Suitable approach:**   - Hypothesis testing when comparing PROM scores to another PROM, PROM scores between sub-groups, or PROM scores over time. | Box 9a or 9b | Table 9 |
|  | Responsiveness  *See COSMIN User Manual for Systematic Reviews, p. 60* | Studies examining the ability of the PROM to detect score changes in the construct to be measured over time.  Note from the COSMIN User Manual, pg. 60: “the only difference between cross‐sectional (construct and criterion) validity and responsiveness is that **validity refers to the validity of a single score**, and **responsiveness refers to the validity of a *change score***.”  Assessments of Responsiveness can take the following approaches:   - *A. Criterion approach*: comparing a population’s PROM scores to their scores on a comparator ‘gold standard’ PROM - *B. Construct Approach*: comparing a population’s PROM scores to their scores on another outcome measure - *C. Construct approach*: comparing PROM scores between sub-groups - *D. Construct approach*: comparing a population’s PROM scores before and after an intervention   **Suitable analyses include:**   - Hypothesis testing - Area Under the Curve (AUC) - Correlations | Box 10a, 10b, 10c, and/or 10d | Table 10, section 10a, 10b, 10c, and 10d |
|  | Concordance | Studies examining the extent to which child abuse scores correspond depending on whether the child, parent, or another respondent is filling in the questionnaire. NOTE: only use where two child abuse measures are used, or when a child abuse self-report measure is compared to registry data (e.g. CPS records). DO NOT use when one measure only measures potential.   - **Example:** a comparison between responses of parent-child dyads to both the parent-reported Parent-Child Conflict Tactics Scale and the child-reported Parent-Child Conflict Tactics Scale.   **Suitable analyses could include:**   - Correlations - Sensitivity & Specificity | Table 11 | |
|  | Interpretability | Studies examining the degree to which one can assign qualitative meaning (i.e. clinical judgements) to a PROM’s quantitative scores or change in scores. The interpretability of a score can be outlined by providing any of the following:   - The distribution of PROM scores in a general population, and floor or ceiling effects - A specific Minimal Important Change (MIC) value - A ‘response shift’^5^ | Table 12 | |
| **Things to note**   - From the COSMIN User guide to systematic reviews, p. 25-6 - ^1^One article may evaluate the same measurement property of the same PROM, but among multiple (sub)groups in different settings or populations. In this case, the same box may need to be filled out multiple times. - From the COSMIN User guide to systematic reviews, p. 47 - ^2^”Structural validity is only relevant for instruments that are based on a reflective model. A reflective model is a model in which all items are a manifestation of the same underlying construct. These kinds of items are called effect indicators. These items are expected to be highly correlated and interchangeable. Its counterpart is a formative model, in which the items together form the construct. These items do not need to be correlated. Therefore, structural validity is not relevant for items that are based on a formative model… Often, authors do not explicitly describe whether their instrument is based on a reflective or formative model. To decide afterwards which model is used, one can do a simple ‘thought test’. With this test one should consider whether all item scores are expected to change when the construct changes. If yes, the construct can be considered a reflective model. If not, the PROM is probably based on a formative model.” PLEASE NOTE: Reflective indicators are typical of classical test theory (e.g. factor analysis models, cronbach’s alpha); they are invoked in an attempt to account for observed variances or covariances. There will be very few formative models, if any. - From the COSMIN User guide to systematic reviews, p. 53 on reliability - ^3^ Note that when comparing subscales to assess a PROM’s reliability, this is not the same as assessing internal consistency: in the context of testing reliability, subscales/item sets are used to see if they are equally able to identify differences between respondents from the target population, and different analytical techniques are used. - ^4^ These are the analyses defined as being eligible according to COSMIN’s updated criteria for good measurement properties (COSMIN User manual p. 28), but there could be other analyses that count as assessment of reliability – studies will simply be scored with ‘?’ if ICCs or Weighted Kappas are not reported - From the COSMIN User guide to systematic reviews, p. 44 on Interpretability & Feasibility - ^5^ Response shift = “changes in the meaning of one's self evaluation of a target construct as a result of: (a) a change in the patient's internal standards of measurement (i.e. scale recalibration); (b) a change in the patient's values (i.e. the importance of component subdomains constituting the target construct); or (c) a redefinition of the target construct (i.e. reconceptualization)” | | | | |

| For further instructions on how to classify which types of studies are assessing which psychometric properties of a measure, see the **COSMIN Taxonomy** and the *COSMIN Methodology for Systematic Reviews of Patient-Reported Outcome Measures (PROMS) User Manual Version 1.0*, **page 26-27** |
| --- |

# Part 4. Extraction & Risk of Bias Assessment for results of each measurement property study

Below are tailored data extraction boxes for the results of each study of the PROM’s measurement property. With reference to **table 3** above, please **delete irrelevant boxes and/or duplicate relevant boxes as appropriate**.

**Some notes on use:**

- If the article does not report information asked for in any of the forms, please enter ‘**NR**’ into the cell to indicate that it was not reported.
- If pasting author’s words directly into the form, please use quotation marks and include the page number where the quote can be found.
- Where the forms ask for quantitative data to be entered, it is acceptable to add screenshots of tables provided these are clear and time-saving. Please note the page number where the table can be found.

**Note on Risk of Bias ratings (from User manual for Assessing Content Validity of PROMs, p. 16)**

- ‘The “worst score counts” method is used in all COSMIN boxes because poor methodological aspects of a study cannot be compensated by good aspects. In defining the response options, the ‘‘worst score counts’’ method was taken into consideration. Only fatal flaws in the design or statistical analyses were regarded as inadequate quality.’

# Studies on Structural Validity

| **Table 3. Data extraction for Studies on Structural Validity** | | | | | | | | | | | | | | | | | |
| --- | --- | --- | --- | --- | --- | --- | --- | --- | --- | --- | --- | --- | --- | --- | --- | --- | --- |
| 3.1 Results | | | | | | | | | | | | | | | | | |
| Approach(es) used (*delete as appropriate*) | | | | | | | | | | | | | | | | | |
| - Classical Test Theory - Confirmatory Factor Analysis - Exploratory Factor Analysis - Principal Component Analysis - Item Response Theory - Rasch - Mokken scale analysis   Other IRT model | | | | | | | | | | | | | | | | | |
| **Classical Test theory (*please delete if irrelevant*)** | | | | | | | | | | | | | | | | | |
| Analysis & Method used (*delete as appropriate & expand if needed)*: | | Sample size: | | | | Factors Tested/Identified (*please list, by name, the factors explored or identified)* | | | | | Measure(s) reported (*delete as appropriate and include result, e.g. CFI >0.95 or RMSEA <0.06)*: | | | | | | |
| - Confirmatory Factor Analysis - Exploratory Factor Analysis - Principal Component Analysis | |  | | | |  | | | | | - Comparative Fit Index (CFI) - Tucker-Lewis Index (TLI) - Root Mean Square Error of Approximation (RMSEA) - Standardized Root Mean Residuals (SRMR**)** - Comparable measure (*please specify*) - Not reported by authors | | | | | | |
| Results – quantitative (*please include quantitative results – this can include screenshots of tables)* | | | | | | | | | | | | | | | | | |
|  | | | | | | | | | | | | | | | | | |
| Results – narrative (*please summarise how the results should be interpreted, e.g. in your own words or the authors’)* | | | | | | | | | | | | | | | | | |
|  | | | | | | | | | | | | | | | | | |
| **Item Response theory (*please delete if irrelevant)*** | | | | | | | | | | | | | | | | | |
| Analysis used (*delete as appropriate)*: | | | | | | Sample size | | | | **Assessment of Unidimensionality** | | | | | | | |
|  |  |  |  |  |  |  |  |  |  | Statistical measure reported (*delete as appropriate and enter results, e.g. RMSEA <0.06*) | | | | | | | |
| - Rasch - Mokken scale analysis - Other IRT model (*please specify*) | | | | | |  | | | | - Comparative Fit Index (CFI) - Tucker-Lewis Index (TLI) - Other comparable measure - Root Mean Square Error of Approximation (RMSEA) - Standardized Root Mean Residuals (SRMR**)** - Not conducted by authors | | | | | | | |
| **Analysis of Local Independence:** | | | | | | | **Evidence of Monotonicity** | | | | | | | **Assessment of Model Fit** | | | |
| Measure(s) reported (*delete as appropriate and include result(s), e.g. r<0.20)*: | | | | | | | Evidence reported (*delete as appropriate and include results, e.g. scalability >0.30)*: | | | | | | | Measure(s) used (*delete as appropriate and include results)*: | | | |
| - Residual correlations among items, controlling for the dominant factor - Yen’s Q3 - None | | | | | | | - Item scalability - Graph of an item characteristic curve (ICC) with an appropriate ‘S’ shape^a^ - None | | | | | | | - **For IRT models:** X^2^ value - **For Rasch:** infit and outfit mean squares - **For Rasch:** Z-standardized values - None | | | |
| Reasons for result rating: (*briefly explain your rationale below*) | | | | | | | | | | | | | | | Result rating (+, ?, -) | | |
|  | | | | | | | | | | | | | | |  | | |
| 3.2 Guide to assessing results | | | | | | | | | | | | | | | | | |
| **Approach** | | | **Rating** | | **Criteria** | | | | | | | | | | | | |
| Classical Test Theory (CTT) | | | **+** | | - Confirmatory Factor Analysis (CFA) or Exploratory Factor Analysis (EFA) (*please note that principal component analysis (PCA) is a form of exploratory factor analysis*) finding **one** of the following: - Comparative Fit Index (CFI) **>0.95** - Tucker-Lewis Index (TLI) **>0.95** - Comparable measure **>0.95** - Root Mean Square Error of Approximation (RMSEA) **<0.06** - Standardized Root Mean Residuals (SRMR**) <0.08**^b^ | | | | | | | | | | | | |
|  |  |  | **?** | | Not all information for ‘+’ reported | | | | | | | | | | | | |
|  |  |  | **-** | | Criteria for ‘+’ not met | | | | | | | | | | | | |
| Rasch/Item Response Theory (IRT) | | | **+** | | **Must meet criteria 1-4**   1. **No violation of unidimensionality**^c^ **–** Must report one of the following:  - Comparative Fit Index (CFI) **>0.95** - Tucker-Lewis Index (TLI) **>0.95** - Comparable measure **>0.95** - Root Mean Square Error of Approximation (RMSEA) **<0.06** - Standardized Root Mean Residuals (SRMR**) <0.082**  1. **No violation of local independence –** Must report one of the following:  - Residual correlations among the items after controlling for the dominant factor <0.20 - Q3’s <0.37  1. **No violation of monotonicity –** Must report one of the following:  - Adequate looking graphs - Item scalability >0.30 (as in the case of e.g. a Mokken analysis)  1. **Adequate model fit –** Must report one of the following:  - ***For IRT specifically:*** X^2^ >0.01 - ***For Rasch specifically:*** - infit and outfit mean squares ≥ 0.5 and ≤ 1.5 - Z-standardized values > ‐2 and <2 | | | | | | | | | | | | |
|  |  |  | **?** | | Model fit not reported | | | | | | | | | | | | |
|  |  |  | **-** | | Criteria for ‘+’ not met | | | | | | | | | | | | |
| Note:  ^a^**Note, taken from Yang 2014 <https://www.ncbi.nlm.nih.gov/pmc/articles/PMC4118016/>:** “One assumption of IRT is monotonicity, which is best displayed on a graph as a curve shaped like an ‘S’ between the latent trait level on the X-axis and the probability of a more extreme response on the item (e.g., a question about depression) on the Y-axis. This curve, called an item characteristic curve (ICC), is assumed to graphically depict the true relationship between the trait and the responses to the item. In our example, the ICC is assumed to reflect the true monotonic relationship between the patient’s level of positive affect (the latent trait) and the patient’s responses to the three CES-D questions.”  ^b^To rate the quality of the summary score, the factor structures should be equal across studies  ^c^unidimensionality refers to a factor analysis per subscale, while structural validity refers to a factor analysis of a (multidimensional) patient‐reported outcome measure | | | | | | | | | | | | | | | | | |
| **Box 3. Risk of Bias Assessment for Studies on Structural Validity** | | | | | | | | | | | | | | | | | |
| Does the scale consist of effect indicators, i.e. is it based on a reflective model? (Delete as appropriate) | | - Yes - No | | | | | | | | **Note:** *If the scale is not based on a reflective model, unidimensionality or structural validity is not relevant.* | | | | | | | |
| Does the study concern unidimensionality or structural validity? (Delete as appropriate) | | - Unidimensionality - structural validity | | | | | | | | **Note:** In a systematic review, it is helpful to make a distinction between studies where factor analysis is performed on each (sub)scale separately to evaluate whether the (sub)scales are unidimensional (unidimensionality studies) and studies where factor analysis is performed on all items of an instrument to evaluate the (expected) number of subscales in the instrument and the clustering of items within subscales (structural validity studies). | | | | | | | |
| *Statistical Methods* | | | | | | | | Very Good | Adequate | | | | Doubtful | | | Inadequate | NA |
| 1. | **For CTT:** was exploratory or confirmatory factor analysis performed? | | | | | | | Confirmatory factor analysis performed | Exploratory factor analysis (EFA) performed (please note: principal component analysis is a form of EFA) | | | | Cell not relevant - do not use | | | No exploratory or confirmatory factor analysis performed | N/A |
| 2. | **For IRT/Rasch:** does the chosen model fit the research question? | | | | | | | Chosen model fits well to the research question | Assumable that the chosen model fits well to the research question | | | | Doubtful if the chosen model fits well to the research question | | | Chosen model does not fit to the research question | N/A |
| 3. | Was the sample size included in the analysis adequate? | | | Factor analysis: | | | | 7 times the number of items and ≥100 | at least 5 times the number of items and ≥100; OR at least 6 times number of  items but <100 | | | | 5 times the number of items  but <100 | | | < 5 times the  number of items | Cell not relevant - do not use |
|  |  |  |  | Rasch/1PL models: | | | | ≥ 200 subjects | 100-199 subjects | | | | 50-99 subjects | | | <50 subjects |  |
|  |  |  |  | 2PL parametric IRT models OR Mokken scale analysis: | | | | ≥ 1000 subjects | 500-999 subjects | | | | 250-499 subjects | | | <250 subjects | Cell not relevant - do not use |
| *Analyses* | | | | | | | | | | | | | | | | | |
| 4. | Were there any other important flaws in the design or statistical methods of the study? | | | | | | | No other important methodological flaws | Cell not relevant - do not use | | | | Other minor methodological flaws (e.g. rotation method not described) | | | Other important methodological flaws (e.g. inappropriate rotation method) | Cell not relevant - do not use |
| **Final Score (based on the “worst score counts” principle)** | | | | | | | | | | | | | | | | | |
| What is the lowest rating of any of the standards in box 3? | | | | | | | | | | | |  | | | | | |
| Any further notes on reasons for the score? | | | | | | | | | | | | | | | | | |
|  | | | | | | | | | | | | | | | | | |

| For further instructions on how to evaluate the structural validity of the measure, see the *COSMIN Methodology for Systematic Reviews of Patient-Reported Outcome Measures (PROMS) User Manual Version 1.0*, **page 38** |
| --- |

| For further instructions on how to assess the Risk of Bias in a study on structural validity, see the *COSMIN Methodology for Systematic Reviews of Patient-Reported Outcome Measures (PROMS) User Manual Version 1.0*, **page 47** |
| --- |

# Studies on Internal Consistency

| **Table 4. Data extraction for Studies of** **Internal Consistency** | | | | | | | | | | | |
| --- | --- | --- | --- | --- | --- | --- | --- | --- | --- | --- | --- |
| 4.1 Results | | | | | | | | | | | |
| Number of unidimensional (sub)scales: | | | | Which approach was used? (e.g. comparing subscales, splitting scale in half, comparing different items in same scale) | | | | | | | |
|  | | | |  | | | | | | | |
| Analysis 1 for unidimensional subscale 1 (*replace underlined area with subscale name if available)* | | | | | | | | | | | |
| Score type (*delete as appropriate*) | | | Analysis type: (*delete as appropriate)* | | | Output (e.g. alpha value >0.70) | | | | | |
| - Continuous - Binary/Dichotomous - Item Response Theory (IRT)-based | | | - Cronbach’s Alpha - Omega - KR-20 - Raykov’s Rho - Standard Error of the Theta (SE (θ)) | | |  | | | | | |
| Analysis 2… (*please replicate the cells above to summarise any further analyses of subscales)* | | | | | | | | | | | |
| Score type (*delete as appropriate*) | | | Analysis type: (*delete as appropriate)* | | | Output (e.g. alpha value >0.70) | | | | | |
|  | | |  | | |  | | | | | |
| Reasons for result rating: (*briefly explain your rationale below*) | | | | | | | | | Result rating (+, ?, -) | | |
|  | | | | | | | | |  | | |
| 4.2 Guide to assessing results | | | | | | | | | | | |
| **Rating** | | **Criteria** | | | | | | | | | |
| **+** | | Must have:   - At least low evidence^4^ for sufficient structural validity^5^ - Cronbach’s alpha(s) of ≥0.70 for each unidimensional scale or subscale | | | | | | | | | |
| **?** | | Criteria for “at least low evidence^4^ for sufficient structural validity” not met | | | | | | | | | |
| **-** | | Has:   - At least low evidence^4^ for sufficient structural validity^5^ - Cronbach’s alpha(s) <0.70 for each unidimensional scale or subscale | | | | | | | | | |
| Notes:  ^4^’Low evidence’ refers to the grading of the evidence according to the GRADE approach – this is only relevant during the pooling of evidence for each PROM  ^5^ When considering this, evidence can come from any other available studies on the structural validity of the PROM | | | | | | | | | | | |
| **Box 4. Risk of Bias Assessment for Studies of Internal consistency** | | | | | | | | | | | |
| Does the scale consist of effect indicators, i.e. is it based on a reflective model? (Delete as appropriate) | | | - Yes - No | | **Note:** *If the scale is not based on a reflective model, internal consistency is not relevant* | | | | | | |
| *Design Requirements* | | | | Very Good | | Adequate | | Doubtful | | Inadequate | NA |
| 1. | Was an internal consistency statistic calculated for each unidimensional scale or subscale separately? | | | Internal consistency statistic calculated for each unidimensional scale or subscale | | Cell not relevant - do not use | | Unclear whether scale or sub scale is unidimensional | | Internal consistency statistic  NOT calculated for each unidimensional scale or sub  scale | Cell not relevant - do not use |
| *Statistical Methods* | | | | | | | | | | | |
| 2. | **For continuous scores:** Was cronbach’s alpha or omega calculated? | | | Cronbach’s alpha, or Omega calculated | | Cell not relevant - do not use | | Only item-total correlations calculated | | No Cronbach’s alpha and no item-total correlations calculated | N/A |
| 3. | **For dichotomous scores:** Was cronbach’s alpha or KR‐20 calculated? | | | Cronbach’s alpha or KR-20 calculated | | Cell not relevant - do not use | | Only item-total correlations calculated | | No cronbach’s alpha or KR-20 and no item-total correlations calculated | N/A |
| 4. | **For IRT‐based scores:** Was standard error of the theta (SE (θ)) or reliability coefficient of estimated latent trait value (index of (subject or item) separation) calculated? | | | SE(θ) or reliability coefficient calculated | | Cell not relevant - do not use | | Cell not relevant - do not use | | SE(θ) or reliability coefficient NOT calculated | N/A |
| *Other* | | | | | | | | | | | |
| 5. | Were there any other important flaws in the design or statistical methods of the study? | | | No other important methodological flaws | | Cell not relevant - do not use | | Other minor methodological flaws | | Other important methodological flaws | Cell not relevant - do not use |
| **Final Score (based on the “worst score counts” principle)** | | | | | | | | | | | |
| What is the lowest rating of any of the standards in box 4? | | | | | | |  | | | | |
| Any further notes on reasons for the score? | | | | | | | | | | | |
|  | | | | | | | | | | | |

| For further instructions on how to evaluate the internal consistency of the measure, see the *COSMIN Methodology for Systematic Reviews of Patient-Reported Outcome Measures (PROMS) User Manual Version 1.0*, **page 38** |
| --- |

| For further instructions on how to assess the Risk of Bias in a study on internal consistency, see the *COSMIN Methodology for Systematic Reviews of Patient-Reported Outcome Measures (PROMS) User Manual Version 1.0*, **page 50** |
| --- |

# Studies on Cross-Cultural Validity/Measurement Invariance

| **Table 5. Data extraction for studies on Cross-cultural validity/measurement invariance** | | | | | | | | | | | | | | | | | | |
| --- | --- | --- | --- | --- | --- | --- | --- | --- | --- | --- | --- | --- | --- | --- | --- | --- | --- | --- |
| 5.1 Results | | | | | | | | | | | | | | | | | | |
| Approaches used: (*Delete as appropriate)* | | | | Groups formed/independent variables analysed (e.g. age, sex, ethnicity) | | | | | | Sample size | | | | | Degree of similarity between groups (*please summarize any evidence that the groups were similar across all other variables aside from the group differences being analysed*) | | | |
| - Classical test theory - Multiple group factor analysis - Regression analyses - Item response theory - Differential Item Functioning (DIF) analysis | | | |  | | | | | |  | | | | |  | | | |
| **Classical test theory – Multiple Group Factor analysis (*please delete these cells if irrelevant)*** | | | | | | | | | | | | | | | | | | |
| Analysis & method used (*delete as appropriate and expand if needed)*: | | | | | | | Approach to Equivalence Testing^1^ | | | | | Measure(s) reported (*delete as appropriate and include result, e.g. CFI >0.95 or RMSEA <0.06)*: | | | | | | |
| - Confirmatory Factor Analysis - Exploratory Factor Analysis - Principal Component Analysis | | | | | | | - Configural equivalence - Metric equivalence - Scalar equivalence | | | | | - Comparative Fit Index (CFI) - Tucker-Lewis Index (TLI) - Comparable measure - Root Mean Square Error of Approximation (RMSEA) - Standardized Root Mean Residuals (SRMR**)** - Not reported by authors | | | | | | |
| - Results – Quantitative (*please include quantitative results – this can include screenshots of tables)* | | | | | | | | | | | | | | | | | | |
|  | | | | | | | | | | | | | | | | | | |
| Results – Narrative (*please briefly summarise how the results should be interpreted, either in your words or in the authors’)* | | | | | | | | | | | | | | | | | | |
|  | | | | | | | | | | | | | | | | | | |
| **Differential Item Functioning analysis (*please delete these cells if irrelevant)*** | | | | | | | | | | | | | | | | | | |
| Statistical approach (*please specify which methods were used for e.g. DIF detection, DIF magnitude, etc*) | | | | | | Criterion for evidence of DIF (expressed as minimum % change in β_1_) | | | | | | | | | | Were sensitivity analyses conducted? (*if so, please specify which methods were used*) | | |
|  | | | | | |  | | | | | | | | | |  | | |
| Detected instances of DIF (*please summarize which items, if any, were found to function differently, including degree of difference and p-values*) | | | | | | | | | Overall Results (please briefly summarise findings as interpreted by authors) | | | | | | | | | |
|  | | | | | | | | |  | | | | | | | | | |
| Reasons for result rating: (*briefly explain your rationale below*) | | | | | | | | | | | Result rating (+,?, -) | | | | | | | |
|  | | | | | | | | | | |  | | | | | | | |
| Notes  ^1^ Equivalence can be categorized according to three hierarchical levels of measurement equivalence, with scalar being the most stringent:   1. Configural equivalence: The factor structure is the same across groups in a multi-group confirmatory factor analysis. 2. Metric equivalence: Factor loadings are similar across groups. 3. Scalar equivalence: Values/Means are also equivalent across groups. | | | | | | | | | | | | | | | | | | |
| 5.2 COSMIN Guide to assessing results | | | | | | | | | | | | | | | | | | |
| **Rating** | | **Criteria** | | | | | | | | | | | | | | | | |
| **+** | | Must have one of the following:   - No important differences found between group factors (such as age, gender, language) in multiple group factor analysis - No important Differential Item Functioning (DIF) for group factors (McFadden’s R^2^ <0.02) | | | | | | | | | | | | | | | | |
| **?** | | No multiple group factor analysis OR Differential Item Functioning (DIF) analysis performed | | | | | | | | | | | | | | | | |
| **-** | | Important differences between group factors OR important Differential item Functioning (DIF) found | | | | | | | | | | | | | | | | |
| **Box 5. Risk of Bias Assessment for Cross-Cultural Validity/Measurement Invariance** | | | | | | | | | | | | | | | | | | |
| *Design Requirements* | | | | | Very Good | | | Adequate | | | | | | Doubtful | | | Inadequate | NA |
| 1. | Were the samples similar for relevant characteristics except for the group variable? | | | | Evidence provided that samples were similar for relevant characteristics  except group variable | | | Stated (but no evidence provided) that samples were similar for relevant characteristics  except group variable | | | | | | Unclear whether samples were similar for relevant characteristics  except group variable | | | Samples were NOT similar for relevant characteristics  except group variable | Cell not relevant - do not use |
| *Statistical Methods* | | | | | | | | | | | | | | | | | | |
| 2. | Was an appropriate approach used to analyse the data? | | | | A widely  recognized or well  justified approach  was used | | | Assumable that the approach was appropriate, but not clearly described | | | | | | Not clear what approach was used or doubtful whether the approach was appropriate | | | Approach not appropriate | N/A |
| 3. | Was the sample size included in the analysis adequate? | | Regression analyses or IRT/Rasch-based analyses: | | 200 subjects per group | | | 150 subjects per group | | | | | | 100 subjects per group | | | <100 subjects per group | Cell not relevant - do not use |
|  |  |  | Multi-group confirmatory factor analyses (MGCFA): | | 7 times the number of items and ≥100 | | | 5 times the number  of items and ≥100;  OR 5‐7 times the number of items but <100 | | | | | | 5 times the number of items but <100 | | | <5 times the number of items | Cell not relevant - do not use |
| *Other* | | | | | | | | | | | | | | | | | | |
| 4. | Were there any other important flaws in the design or statistical methods of the study? | | | | No other important methodological flaws | | | Cell not relevant - do not use | | | | | | Other minor methodological flaws | | | Other important methodological flaws | Cell not relevant - do not use |
| **Final Score (based on the “worst score counts” principle)** | | | | | | | | | | | | | | | | | | |
| What is the lowest rating of any of the standards in box 5? | | | | | | | | | | | | |  | | | | | |
| Any further notes on reasons for the score? | | | | | | | | | | | | | | | | | | |
|  | | | | | | | | | | | | | | | | | | |

| For further instructions on how to evaluate the measurement invariance/cross-cultural validity of the measure, see the *COSMIN Methodology for Systematic Reviews of Patient-Reported Outcome Measures (PROMS) User Manual Version 1.0*, **page 38** |
| --- |

| For further instructions on how to assess the Risk of Bias in a study on cross-cultural validity/measurement invariance, see the *COSMIN Methodology for Systematic Reviews of Patient-Reported Outcome Measures (PROMS) User Manual Version 1.0*, **page 51** |
| --- |

# 4. Studies on Reliability

| **Table 6. Data extraction for Studies on Reliability** | | | | | | | | | | | | |
| --- | --- | --- | --- | --- | --- | --- | --- | --- | --- | --- | --- | --- |
| 6.1 Results (*please include ICC or Kappa if reported)* | | | | | | | | | | | | |
| Type of Reliability tested (*please delete as appropriate)* | | | Approach to testing (*please include details of e.g. the time between test and re-test, approach to inter-rater reliability, similarity of test conditions, etc)*: | | | | | | Approach to analysis (*please summarize the analytical techniques used, eg. Pearson or Spearman Correlation Coefficients, etc)* | | | |
| - Test-retest reliability - Inter-rater reliability - Intra-rater reliability | | |  | | | | | |  | | | |
| Data/scoring type (*delete as appropriate)*: | | | | Were Cohen’s (weighted) Kappa or Intraclass Correlation Coefficients calculated? (*delete as appropriate)* | | | | | | Additional details (e.g. weighting scheme of weighted Kappa if applicable) | | |
| - Continuous/interval - Categorical - Nominal - Dichotomous - Ordinal | | | | - Cohen’s Kappa (weighted) - Cohen’s Kappa (unweighted) - Intraclass Correlation Coefficient - None calculated | | | | | |  | | |
| Results – Quantitative (*please include quantitative results – this can be screenshots of tables)* | | | | | | | | | | | | |
|  | | | | | | | | | | | | |
| Results – Narrative (*please summarise how the results should be interpreted, either in the author’s words or your own*) | | | | | | | | | | | | |
|  | | | | | | | | | | | | |
| Reasons for result rating: (*briefly explain your rationale below*) | | | | | | | Result rating (+,?, -) | | | | | |
|  | | | | | | |  | | | | | |
| 6.2 COSMIN Guide to assessing results | | | | | | | | | | | | |
| **Rating** | | **Criteria** | | | | | | | | | | |
| **+** | | Must have one of the following:   - For continuous scores: Intraclass Correlation Coefficient (ICC) **≥0.70** - For ordinal scores: weighted Kappa **≥0.70** | | | | | | | | | | |
| **?** | | - Intraclass Correlation Coefficient (ICC) or weighted kappa not reported | | | | | | | | | | |
|  | | - Intraclass Correlation Coefficient (ICC) or weighted kappa **<0.70** | | | | | | | | | | |
| **Box 6. Risk of Bias Assessment for Studies on Reliability** | | | | | | | | | | | | |
| *Design Requirements* | | | | | Very Good | Adequate | | Doubtful | | | Inadequate | NA |
| 1. | Were respondents from the target population stable in the interim period on the construct to be measured? | | | | Evidence provided that respondents from the target population were stable | Assumable that respondents from the target population were stable | | Unclear if respondents from the target population were stable | | | Respondents from the target population were NOT stable | Cell not relevant - do not use |
| 2. | Was the time interval appropriate? | | | | Time interval  appropriate | Cell not relevant - do not use | | Doubtful whether time interval was appropriate or time interval was not stated | | | Time interval NOT appropriate | Cell not relevant - do not use |
| 3. | Were the test conditions similar for the instruments? E.g. type of administration, environment, instructions. | | | | Test conditions  were similar (evidence provided) | Assumable that test conditions  were similar | | Unclear if test conditions  were similar | | | Test conditions  were NOT similar | Cell not relevant - do not use |
| *Statistical Methods* | | | | | | | | | | | | |
| 4. | **For continuous scores:** Was an intraclass correlation coefficient (ICC) calculated? | | | | ICC calculated and  model or formula  of the ICC is  described | ICC calculated but  model or formula of the ICC not described or not optimal. Pearson or Spearman correlation coefficient calculated with evidence provided  that no systematic  change has occurred | | Pearson or Spearman  correlation coefficient  calculated WITHOUT evidence provided  that no systematic  change has occurred or WITH evidence that systematic change has occurred | | | No ICC or Pearson or  Spearman correlations calculated | N/A |
| 5. | **For dichotomous/ nominal/ ordinal scores:** Was kappa calculated? | | | | Kappa calculated | Cell not relevant - do not use | | Cell not relevant - do not use | | | No kappa calculated | N/A |
| 6. | **For ordinal scores:** Was a weighted kappa calculated? | | | | Weighted Kappa calculated |  | | Unweighted Kappa calculated or not described | | | Cell not relevant - do not use | N/A |
| 7. | **For ordinal scores:** Was the weighting scheme described? e.g.  linear, quadratic | | | | Weighting scheme described | Weighting scheme NOT described | | Cell not relevant - do not use | | | Cell not relevant - do not use | N/A |
| *Other* | | | | | | | | | | | | |
| 8. | Were there any other important flaws in the design or statistical methods of the study? | | | | No other important methodological flaws | Cell not relevant - do not use | | Other minor methodological flaws | | | Other important methodological flaws | Cell not relevant - do not use |
| **Final Score (based on the “worst score counts” principle)** | | | | | | | | | | | | |
| What is the lowest rating of any of the standards in box 6? | | | | | | | |  | | | | |
| Any further notes on reasons for the score? | | | | | | | | | | | | |
|  | | | | | | | | | | | | |

| For further instructions on how to evaluate the reliability of the measure, see the *COSMIN Methodology for Systematic Reviews of Patient-Reported Outcome Measures (PROMS) User Manual Version 1.0*, **page 40** |
| --- |

| For further instructions on how to assess the Risk of Bias in a study on reliability, see the *COSMIN Methodology for Systematic Reviews of Patient-Reported Outcome Measures (PROMS) User Manual Version 1.0*, **page 53** |
| --- |

# 5. Studies on Measurement Error

| **Table 7. Data extraction for Studies on Measurement Error** | | | | | | | | | | | |
| --- | --- | --- | --- | --- | --- | --- | --- | --- | --- | --- | --- |
| **7.1 Results** | | | | | | | | | | | |
| Approach to assessment of measurement error (*please summarise if available e.g. the interim period, the stability of respondents from the target population over time, & the similarity of test conditions over time)* | | | | | Data/Scoring type (*delete as appropriate)* | | Analytical technique used (*please delete/amend as appropriate)* | | | | |
|  | | | | | - Continuous/interval - Categorical - Nominal - Dichotomous - Ordinal | | - Comparison of Minimal Important Change (MIC) to: - Smallest Detectable Change (SDC) - Limits of Agreement (LoA) - Standard error of measurement (SEM) - Percentage (%) of agreement (positive and negative) - Other (*please describe*) | | | | |
| **For Continuous Scores (*please delete these boxes if not applicable*)** | | | | | | | | | | | |
| Measures calculated (*delete as appropriate and include results)* | | | Results – Narrative | | | | | | | | |
| - MIC: - SDC: - LoA: - SEM: | | |  | | | | | | | | |
| **For Nominal, Dichotomous, or Ordinal Scores** | | | | | | | | | | | |
| Percentage agreement (*please include positive and negative agreement)* | | | Results – Narrative | | | | | | | | |
|  | | |  | | | | | | | | |
| Reasons for result rating: (*briefly explain your rationale below*) | | | | | | | Result rating (+,?, -) | | | | |
|  | | | | | | |  | | | | |
| 7.2 COSMIN Guide to assessing results | | | | | | | | | | | |
| **Rating** | | **Criteria** | | | | | | | | | |
| **+** | | Must have one of the following:   - Smallest Detectable Change > Minimal Important Change^5^ - Limits of Agreement > Minimal Important Change^5^ | | | | | | | | | |
| **?** | | Minimal Important Change^5^ not defined | | | | | | | | | |
| **-** | | Has one of the following:   - Smallest Detectable Change < Minimal Important Change^5^ - Limits of Agreement < Minimal Important Change^5^ | | | | | | | | | |
| Note:  ^5^The Minimal Important Change can be sourced from other studies of the PROM if available | | | | | | | | | | | |
| **Box 7. Risk of Bias Assessment for Studies on Measurement Error** | | | | | | | | | | | |
| *Design Requirements* | | | | Very Good | | Adequate | | | Doubtful | Inadequate | NA |
| 1. | Were respondents from the target population stable in the interim period on the construct to be measured? | | | Evidence provided that respondents from the target population were stable | | Assumable that respondents from the target population were stable | | | Unclear if respondents from the target population were stable | Respondents from the target population were NOT stable | Cell not relevant - do not use |
| 2. | Was the time interval appropriate? | | | Time interval  appropriate | | Cell not relevant - do not use | | | Doubtful whether time interval was appropriate or time interval was not stated | Time interval NOT appropriate | Cell not relevant - do not use |
| 3. | Were the test conditions similar for the instruments? E.g. type of administration, environment, instructions. | | | Test conditions  were similar (evidence provided) | | Assumable that test conditions  were similar | | | Unclear if test conditions  were similar | Test conditions  were NOT similar | Cell not relevant - do not use |
| *Statistical Methods* | | | | | | | | | | | |
| 4. | For continuous scores: Was the Standard Error of Measurement (SEM), Smallest Detectable Change (SDC) or Limits of Agreement (LoA) calculated? | | | SEM, SDC, or LoA calculated | | Possible to calculate LoA from the data presented | | | Cell not relevant - do not use | SEM calculated  based on Cronbach’s alpha, or on SD from another population | N/A |
| 5. | For dichotomous/nominal/ordinal scores: Was the percentage (positive and negative) agreement calculated? | | | % positive and  negative agreement  calculated | | % agreement calculated | | | Cell not relevant - do not use | % agreement not calculated | N/A |
| *Other* | | | | | | | | | | | |
| 6. | Were there any other important flaws in the design or statistical methods of the study? | | | No other important methodological flaws | | Cell not relevant - do not use | | | Other minor methodological flaws | Other important methodological flaws | Cell not relevant - do not use |
| **Final Score (based on the “worst score counts” principle)** | | | | | | | | | | | |
| What is the lowest rating of any of the standards in box 7? | | | | | | | |  | | | |
| Any further notes on reasons for the score? | | | | | | | | | | | |
|  | | | | | | | | | | | |

| For further instructions on how to evaluate measurement error of the measure, see the *COSMIN Methodology for Systematic Reviews of Patient-Reported Outcome Measures (PROMS) User Manual Version 1.0*, **page 38** |
| --- |

| For further instructions on how to assess the Risk of Bias in a study on measurement error, see the *COSMIN Methodology for Systematic Reviews of Patient-Reported Outcome Measures (PROMS) User Manual Version 1.0*, **page 56** |
| --- |

# 6. Studies on Criterion Validity

| **Table 8. Data extraction for Studies on Criterion Validity**  ***Please only use in instances where a modified (e.g. shortened or adapted) version of a PROM is compared to the original*** | | | | | | | | | | | | |
| --- | --- | --- | --- | --- | --- | --- | --- | --- | --- | --- | --- | --- |
| 8.1 Results | | | | | | | | | | | | |
| Comparator measure used | | | |  | | | | | | | | |
| Data/Scoring type: | | | Sample size (response rate) | | | Analysis used (*delete/amend as appropriate)* | | | | | | |
| - Continuous - Dichotomous | | |  | | | - Correlation coefficient - Area Under the Receiver Operating Curve (AUC) analysis - Sensitivity and Specificity analysis - Other (*please describe)* | | | | | | |
| Results – Narrative (*please summarise how the results should be interpreted, in the author’s words or your own)* | | | | | | | | | | | | |
|  | | | | | | | | | | | | |
| Results – Quantitative (*please summarise quantitative results – this can be screenshots)* | | | | | | | | | | | | |
|  | | | | | | | | | | | | |
| Reasons for result rating: (*briefly explain your rationale below*) | | | | | | | | Result rating (+,?, -) | | | | |
|  | | | | | | | |  | | | | |
| 8.2 COSMIN Guide to assessing results | | | | | | | | | | | | |
| **Rating** | | **Criteria** | | | | | | | | | | |
| **+** | | Must have one of the following:   - Correlation with gold standard **≥0.70** - Area under the curve (AUC) **≥0.70** | | | | | | | | | | |
| **?** | | Not all information for ‘+’ reported | | | | | | | | | | |
| **-** | | Has one of the following:   - Correlation with gold standard **≥0.70** - Area under the curve (AUC) **≥0.70** | | | | | | | | | | |
| **Box 8. Risk of Bias Assessment for Studies on Criterion Validity** | | | | | | | | | | | | |
| *Statistical Methods* | | | | | Very Good | | Adequate | | | Doubtful | Inadequate | NA |
| 1. | For continuous scores: Were correlations, or the area under the receiver operating curve calculated? | | | | Correlations or AUC calculated | | Cell not relevant - do not use | | | Cell not relevant - do not use | Correlations or AUC NOT calculated | N/A |
| 2. | For dichotomous scores: Were sensitivity and specificity determined? | | | | Sensitivity and  specificity  calculated | | Cell not relevant - do not use | | | Cell not relevant - do not use | Sensitivity and  specificity NOT  calculated | N/A |
| *Other* | | | | | | | | | | | | |
| 3. | Were there any other important flaws in the design or statistical methods of the study? | | | | No other important methodological flaws | | Cell not relevant - do not use | | | Other minor methodological flaws | Other important methodological flaws | Cell not relevant - do not use |
| **Final Score (based on the “worst score counts” principle)** | | | | | | | | | | | | |
| What is the lowest rating of any of the standards in box 8? | | | | | | | | |  | | | |
| Any further notes on reasons for the score? | | | | | | | | | | | | |
|  | | | | | | | | | | | | |

| For further instructions on how to evaluate the criterion validity of the measure, see the *COSMIN Methodology for Systematic Reviews of Patient-Reported Outcome Measures (PROMS) User Manual Version 1.0*, **page 40** |
| --- |

| For further instructions on how to assess the Risk of Bias in a study on criterion validity, see the *COSMIN Methodology for Systematic Reviews of Patient-Reported Outcome Measures (PROMS) User Manual Version 1.0*, **page 57** |
| --- |

# 7. Hypothesis Tests for Construct Validity

| **Table 9. Data extraction on Hypothesis testing for construct validity** | | | | |
| --- | --- | --- | --- | --- |
| 9.1 Results | | | | |
| Hypothesis tested (*please summarise the hypotheses as the authors described them, if available)* | | Approach to hypothesis testing (*delete as appropriate):* | | |
|  | | - **Convergent validity** (comparison with another outcome measurement instrument) – *see box 9a* - **Discriminative validity** (detecting known differences between two groups of respondents) – *see box 9b* | | |
| 9.2 Convergent Validity Approach (*please delete this section if irrelevant)* | | | | |
| Comparison PROM | | | | Statistical method used |
|  | | | |  |
| Results – Narrative (*please summarise how the results should be interpreted, in the author’s words or your own)* | | | | |
|  | | | | |
| Results – Quantitative (*please summarise quantitative results – this can be screenshots)* | | | | |
|  | | | | |
| Convergent Validity Result rating | | | | |
| Reasons for result rating: (*briefly explain your rationale below if needed*) | | | Result rating (+,?, -) | |
|  | | |  | |
| 9.3 Discriminative Validity Approach *(please delete this section if irrelevant)* | | | | |
| Known difference between groups *(e.g. presence or absence of suicide attempt history, clinical diagnosis, etc)* | | Statistical method used *(e.g. was testing done to assess the statistical significance of the difference?)* | | |
|  | |  | | |
| Results – Narrative (*please summarise how the results should be interpreted, in the author’s words or your own)* | | | | |
|  | | | | |
| Results – Quantitative (*please summarise quantitative results – this can be screenshots)* | | | | |
|  | | | | |
| Discriminative Validity Result rating | | | | |
| Reasons for result rating: (*briefly explain your rationale below if needed*) | | | Result rating (+,?, -) | |
|  | | |  | |
| COSMIN Guide to assessing results | | | | |
| **Rating** | **Criteria** | | | |
| **+** | The result is in accordance with the hypothesis^7^ | | | |
| **?** | No hypothesis defined by the review team | | | |
| **-** | The result is not in accordance with the hypothesis^7^ | | | |
| Note:  ^7^ If there are multiple studies, the results of all studies should be taken together and it should then be decided if 75% of the results are in accordance with the hypotheses | | | | |

| **Box 9. Risk of Bias Assessment on Hypothesis Testing for Construct Validity** | | | | | | | |
| --- | --- | --- | --- | --- | --- | --- | --- |
| 9a. Comparison with Other Outcome Measurement Instruments (Convergent Validity) | | | | | | | |
| *Design Requirements* | | Very Good | Adequate | Doubtful | | Inadequate | NA |
| 1. | Is it clear what the comparator instrument(s) measure(s)? | Constructs measured by the comparator instrument(s) is clear | Cell not relevant - do not use | Cell not relevant - do not use | | Constructs measured by the comparator instrument(s) is not clear | Cell not relevant - do not use |
| 2. | Were the measurement properties of the comparator instrument(s) sufficient? | Sufficient measurement  properties of the comparator instrument(s) in a population similar to the study population | Sufficient measurement  properties of the comparator instrument(s) but not sure if these apply to the study population | Some information on measurement  properties of the  comparator instrument(s) in any study population | | No information on  the measurement  properties of the  comparator instrument(s), OR  evidence for insufficient measurement  properties of the  comparator instrument(s) | Cell not relevant - do not use |
| *Statistical Methods* | | | | | | | |
| 3. | Was the statistical method appropriate for the hypotheses to be tested? | Statistical method was appropriate | Assumable that statistical method was appropriate | Statistical method applied NOT optimal | | Statistical method applied NOT appropriate | Cell not relevant - do not use |
| *Other* | | | | | | | |
| 4. | Were there any other important flaws in the design or statistical methods of the study? | No other important methodological flaws | Cell not relevant - do not use | Other minor methodological flaws (e.g. only data presented on a comparison with an instrument that measures another construct) | | Other important methodological flaws | Cell not relevant - do not use |
| **Final Score (based on the “worst score counts” principle)** | | | | | | | |
| What is the lowest rating of any of the standards in box 9? | | | | |  | | |
| Any further notes on reasons for the score? | | | | | | | |
|  | | | | | | | |

| Box 9b. Comparison between subgroups (discriminative or known-groups validity) | | | | | | | |
| --- | --- | --- | --- | --- | --- | --- | --- |
| *Design Requirements* | | Very Good | Adequate | Doubtful | | Inadequate | NA |
| 5. | Was an adequate description provided of important characteristics of the subgroups? | Adequate description of the important characteristics of the subgroups | Adequate description of most of the important characteristics of the subgroups | Poor or no description of the important characteristics of the subgroups | | Cell not relevant - do not use | Cell not relevant - do not use |
| *Statistical methods* | | | | | | | |
| 6. | Was the statistical method appropriate for the hypotheses to be tested? | Statistical method was appropriate | Assumable that statistical method was appropriate | Statistical method applied NOT optimal | | Statistical method applied NOT appropriate | Cell not relevant - do not use |
| *Other* | | | | | | | |
| 7. | Were there any other important flaws in the design or statistical methods of the study? | No other important methodological flaws | Cell not relevant - do not use | Other minor methodological flaws | | Other important methodological flaws | Cell not relevant - do not use |
| **Final Score (based on the “worst score counts” principle)** | | | | | | | |
| What is the lowest rating of any of the standards in box 9? | | | | |  | | |
| Any further notes on reasons for the score? | | | | | | | |
|  | | | | | | | |

| For further instructions on how to evaluate the construct validity of the measure, see the *COSMIN Methodology for Systematic Reviews of Patient-Reported Outcome Measures (PROMS) User Manual Version 1.0*, **page 40** |
| --- |

| For further instructions on how to assess the Risk of Bias in a study on construct validity, see the *COSMIN Methodology for Systematic Reviews of Patient-Reported Outcome Measures (PROMS) User Manual Version 1.0*, **page 58** |
| --- |

# 8. Studies on Responsiveness

| **Table 10. Data extraction for Studies on Responsiveness** | | | | | | | | | | | | | | | | | | | | | |
| --- | --- | --- | --- | --- | --- | --- | --- | --- | --- | --- | --- | --- | --- | --- | --- | --- | --- | --- | --- | --- | --- |
| 10.1 Results | | | | | | | | | | | | | | | | | | | | | |
| Approach taken (*delete as appropriate*) | | | | | | | | | | | | | | Data/Score type | | | | | Interval period (*please specify time between each test)* | | |
| - **Criterion approach** – comparison to gold standard PROM - **Construct approach** – hypothesis testing via comparison with another outcome measure - **Construct approach** – hypothesis testing via comparison between subgroups - **Construct approach** – comparison before and after exposure to intervention | | | | | | | | | | | | | | - Dichotomous / binary - Continuous | | | | |  | | |
| **10.a Criterion approach – comparison to a gold standard PROM (*please delete if not applicable)*** | | | | | | | | | | | | | | | | | | | | | |
| Comparator ‘gold standard’ measure used | | | Sample size (response rate) | | | Change scores | | | | | | | | | Statistical method(s) used (*delete as appropriate)* | | | | | | |
|  | | |  | | |  | | | | | | | | | - Correlations - Area under the Receiver Operating Curve (AUC) - Sensitivity and Specificity | | | | | | |
| Results – Narrative (*please summarise how the results should be interpreted, in the author’s words or your own)* | | | | | | | | | | | | | | | | | | | | | |
|  | | | | | | | | | | | | | | | | | | | | | |
| Results – Quantitative (*please summarise quantitative results – this can be screenshots)* | | | | | | | | | | | | | | | | | | | | | |
|  | | | | | | | | | | | | | | | | | | | | | |
| **10.b Construct approach – hypothesis testing via comparison to another outcome measure (*please delete if not applicable)*** | | | | | | | | | | | | | | | | | | | | | |
| Comparator outcome measure used (*please describe relevant details, such as the construct it measures and whether it has been validated)* | | | | | | | | | | | Hypothesis tested | | | | | | | | | | |
|  | | | | | | | | | | |  | | | | | | | | | | |
| Sample size (*please specify sample sizes for both measurement points and include details of attrition if applicable*) | | | | | | | | | Change scores | | | | | | | | Statistical method used | | | | |
|  | | | | | | | | |  | | | | | | | |  | | | | |
| Results – Narrative (*please summarise how the results should be interpreted, in the author’s words or your own)* | | | | | | | | | | | | | | | | | | | | | |
|  | | | | | | | | | | | | | | | | | | | | | |
| Results – Quantitative (*please summarise quantitative results – this can be screenshots)* | | | | | | | | | | | | | | | | | | | | | |
|  | | | | | | | | | | | | | | | | | | | | | |
| **10.c Construct approach – hypothesis testing via comparison between subgroups (*please delete if not applicable)*** | | | | | | | | | | | | | | | | | | | | | |
| Sub-groups examined (*please describe relevant details, such as the construct it measures and whether it has been validated)* | | | | | | | | | | | Hypothesis tested | | | | | | | | | | |
|  | | | | | | | | | | |  | | | | | | | | | | |
| Sample size (*please specify sample sizes for both groups at both measurement points, including details on attrition of applicable*) | | | | | | | | Change scores | | | | | | | | | | Statistical method used | | | |
|  | | | | | | | |  | | | | | | | | | |  | | | |
| Results – Narrative (*please summarise how the results should be interpreted, in the author’s words or your own)* | | | | | | | | | | | | | | | | | | | | | |
|  | | | | | | | | | | | | | | | | | | | | | |
| Results – Quantitative (*please summarise quantitative results – this can be screenshots)* | | | | | | | | | | | | | | | | | | | | | |
|  | | | | | | | | | | | | | | | | | | | | | |
| **10.d Construct approach – comparison before and after intervention (*please delete if not applicable)*** | | | | | | | | | | | | | | | | | | | | | |
| Intervention used (*please include brief details on e.g. the aims, duration, and type of intervention)* | | | | | | | | | | Hypothesis tested (*please explain whether/how authors hypothesize the intervention to impact the PROM)* | | | | | | | | | | | |
|  | | | | | | | | | |  | | | | | | | | | | | |
| Sample size (*please specify sample sizes for both groups at all measurement points, including details on attrition of applicable*) | | | | | Intervention effect size (if measured using a separate PROM) | | | | | | | | Statistical method used (*please delete/amend as appropriate*) | | | | | | | | |
|  | | | | |  | | | | | | | | - Effect size – Odd’s ratio - Effect size – Cohen’s d, hedge’s g - Effect size – β coefficient - ANOVA/ANCOVA - Standardized response mean - Norman’s responsiveness coefficient - Relative efficacy statistic - Other | | | | | | | | |
| Results – Narrative (*please summarise how the results should be interpreted, in the author’s words or your own)* | | | | | | | | | | | | | | | | | | | | | |
|  | | | | | | | | | | | | | | | | | | | | | |
| Results – Quantitative (*please summarise quantitative results – this can be screenshots)* | | | | | | | | | | | | | | | | | | | | | |
|  | | | | | | | | | | | | | | | | | | | | | |
| Reasons for result rating: (*briefly explain your rationale below*) | | | | | | | | | | | | | | | | Result rating (+,?, -) | | | | | |
|  | | | | | | | | | | | | | | | |  | | | | | |
| 10.2 COSMIN Guide to assessing results | | | | | | | | | | | | | | | | | | | | | |
| **Rating** | | **Criteria** | | | | | | | | | | | | | | | | | | | |
| **+** | | Must have one of the following:   - The result is in accordance with the hypothesis^7^ - Area Under the Curve (AUC) **≥**0.70 | | | | | | | | | | | | | | | | | | | |
| **?** | | No hypothesis defined by the review team | | | | | | | | | | | | | | | | | | | |
| **-** | | Has one of the following:   - The result is not in accordance with the hypothesis^7^ - Area Under the Curve (AUC) <0.70 | | | | | | | | | | | | | | | | | | | |
| Note:  ^7^ If there are multiple studies, the results of all studies should be taken together and it should then be decided if 75% of the results are in accordance with the hypotheses | | | | | | | | | | | | | | | | | | | | | |
| **Box 10. Risk of Bias Assessment for Studies on Responsiveness** | | | | | | | | | | | | | | | | | | | | | |
| 10a.Criterion approach- comparison to gold standard (*please delete if irrelevant)* | | | | | | | | | | | | | | | | | | | | | |
| *Statistical Methods* | | | | Very Good | | | Adequate | | | | | Doubtful | | | | | | | | Inadequate | NA |
| 1. | **For continuous scores:** Were correlations between change scores, or the area under the Receiver Operator Curve (ROC) curve calculated? | | | Correlations or Area under the ROC Curve (AUC) calculated | | | Cell not relevant - do not use | | | | | Cell not relevant - do not use | | | | | | | | Correlations or Area under the ROC Curve (AUC) NOT calculated | N/A |
| 2. | **For dichotomous scales:** Were sensitivity and specificity (changed  versus not changed) determined? | | | Sensitivity and  specificity calculated | | | Cell not relevant - do not use | | | | | Cell not relevant - do not use | | | | | | | | Sensitivity and  Specificity NOT calculated | N/A |
| *Other* | | | | | | | | | | | | | | | | | | | | | |
| 3. | Were there any other important flaws in the design or statistical methods of the study? | | | No other important methodological flaws | | | Cell not relevant - do not use | | | | | Other minor methodological flaws | | | | | | | | Other important methodological flaws | Cell not relevant - do not use |
| **Final Score (based on the “worst score counts” principle)** | | | | | | | | | | | | | | | | | | | | | |
| What is the lowest rating of any of the standards in box 10a? | | | | | | | | | | | | | | | |  | | | | | |
| Any further notes on reasons for the score? | | | | | | | | | | | | | | | | | | | | | |
|  | | | | | | | | | | | | | | | | | | | | | |

| 10b. Construct approach - hypothesis testing; comparison with other outcome measures (*please* *delete if irrelevant*) | | | | | | | |
| --- | --- | --- | --- | --- | --- | --- | --- |
| *Design Requirements* | | Very Good | Adequate | Doubtful | | Inadequate | NA |
| 4. | Is it clear what the comparator instrument(s) measure(s)? | Constructs measured by the comparator instrument(s) is clear | Cell not relevant - do not use | Cell not relevant - do not use | | Constructs measured by the comparator instrument(s) is not clear | Cell not relevant - do not use |
| 5. | Were the measurement properties of the comparator instrument(s) sufficient? | Sufficient measurement  properties of the comparator instrument(s) in a population similar to the study population | Sufficient measurement  properties of the comparator instrument(s) but not sure if these apply to the study population | Some information on measurement  properties of the  comparator instrument(s) in any study population | | No information on  the measurement  properties of the  comparator instrument(s), OR  evidence of poor quality of  comparator instrument(s) | Cell not relevant - do not use |
| *Statistical methods* | | | | | | | |
| 6. | Was the statistical method appropriate for the hypotheses to be tested? | Statistical method was appropriate | Assumable that statistical method was appropriate | Statistical method applied NOT optimal | | Statistical method applied NOT appropriate | Cell not relevant - do not use |
| *Other* | | | | | | | |
| 7. | Were there any other important flaws in the design or statistical methods of the study? | No other important methodological flaws | Cell not relevant - do not use | Other minor methodological flaws | | Other important methodological flaws | Cell not relevant - do not use |
| **Final Score (based on the “worst score counts” principle)** | | | | | | | |
| What is the lowest rating of any of the standards in box 10b? | | | | |  | | |
| Any further notes on reasons for the score? | | | | | | | |
|  | | | | | | | |

| 10c. Construct approach - hypothesis testing; comparison between subgroups (*please delete if irrelevant)* | | | | | | | |
| --- | --- | --- | --- | --- | --- | --- | --- |
| *Design Requirements* | | Very Good | Adequate | Doubtful | | Inadequate | NA |
| 8. | Was an adequate description provided of important characteristics of the subgroups? | Adequate description of the important characteristics of the subgroups | Adequate description of most of the important characteristics of the subgroups | Poor or no description of the important characteristics of the subgroups | | Cell not relevant - do not use | Cell not relevant - do not use |
| *Statistical methods* | | | | | | | |
| 9. | Was the statistical method appropriate for the hypotheses to be tested? | Statistical method was appropriate | Assumable that statistical method was appropriate | Statistical method applied NOT optimal | | Statistical method applied NOT appropriate | Cell not relevant - do not use |
| *Other* | | | | | | | |
| 10. | Were there any other important flaws in the design or statistical methods of the study? | No other important methodological flaws | Cell not relevant - do not use | Other minor methodological flaws | | Other important methodological flaws | Cell not relevant - do not use |
| **Final Score (based on the “worst score counts” principle)** | | | | | | | |
| What is the lowest rating of any of the standards in box 10c? | | | | |  | | |
| Any further notes on reasons for the score? | | | | | | | |
| \|  \| \| --- \| | | | | | | | |

| 10d. Construct approach (i.e. hypothesis testing; before and after intervention) | | | | | | | |
| --- | --- | --- | --- | --- | --- | --- | --- |
| *Design Requirements* | | Very Good | Adequate | Doubtful | | Inadequate | NA |
| 11. | Was an adequate description provided of the intervention given? | Adequate description of the  intervention | Cell not relevant - do not use | Poor description of  the intervention | | NO description of  the intervention | Cell not relevant - do not use |
| *Statistical methods* | | | | | | | |
| 12. | Was the statistical method appropriate for the hypotheses to be tested? | Statistical method was appropriate | Assumable that statistical method was appropriate | Statistical method applied NOT optimal | | Statistical method applied NOT appropriate | Cell not relevant - do not use |
| *Other* | | | | | | | |
| 13. | Were there any other important flaws in the design or statistical methods of the study? | No other important methodological flaws | Cell not relevant - do not use | Other minor methodological flaws | | Other important methodological flaws | Cell not relevant - do not use |
| **Final Score (based on the “worst score counts” principle)** | | | | | | | |
| What is the lowest rating of any of the standards in box 10d? | | | | |  | | |
| Any further notes on reasons for the score? | | | | | | | |
|  | | | | | | | |

| For further instructions on how to evaluate the responsiveness of the measure, see the *COSMIN Methodology for Systematic Reviews of Patient-Reported Outcome Measures (PROMS) User Manual Version 1.0*, **page 40** |
| --- |

| For further instructions on how to assess the Risk of Bias in a study on structural validity, see the *COSMIN Methodology for Systematic Reviews of Patient-Reported Outcome Measures (PROMS) User Manual Version 1.0*, **page 60** |
| --- |

# 9. Studies on Concordance

| **Table 11. Data extraction for Studies on Concordance** | | | |
| --- | --- | --- | --- |
| **11.1 Results** | | | |
| Sample Size | Respondents compared (e.g. parent- vs child-report) | | Measures Compared |
|  |  | |  |
| Analysis & method used (*delete/amend and expand as appropriate)* | | Results – Narrative (*please summarise how the results should be interpreted, in the author’s words or your own)* | |
| - Correlation coefficient - Sensitivity and Specificity analysis - Other (*please describe)* | |  | |
| Results – Quantitative (*please summarise quantitative results – this can be screenshots)* | | | |
|  | | | |

# 10. Studies addressing Interpretability

| **Table 12. Data extraction for Studies addressing Interpretability** | | | | |
| --- | --- | --- | --- | --- |
| PROM Interpretability | | | | |
| **Distribution of scores in study population** | **Percentage of missing items** | **Percentage of missing total scores** | **Floor effects** | **Ceiling Effects** |
|  |  |  |  |  |
| **Scores available for relevant sub-groups?** | **Change scores available for relevant sub-groups?** | **Minimal Important Change (MIC)** | **Minimal Important Difference (MID)** | **Response Shift** |
|  |  |  |  |  |

| For further information on how a PROM’s interpretability is defined and what information to gather, see the *COSMIN Methodology for Systematic Reviews of Patient-Reported Outcome Measures (PROMS) User Manual Version 1.0*, **pages 44 and 68** |
| --- |
