# Supplementary material for: Measuring Violence Against Children: A COSMIN Systematic Review of the Psychometric and Administrative Properties of Adult Retrospective Self-report Instruments on Child Abuse and Neglect
Source: Trauma Violence Abuse. 2023 Jan 25;25(1):183–96. doi: 10.1177/15248380221145912 (PMC10666516; doi:10.1177/15248380221145912)
Supplement: sj-docx-5-tva-10.1177_15248380221145912 – Supplemental material for Measuring Violence Against Children: A COSMIN Systematic Review of the Psychometric and Administrative Properties of Adult Retrospective Self-report Instruments on Child Abuse and Neglect [file sj-docx-5-tva-10.1177_15248380221145912.docx]

Data extraction form for Phase 2: Overview of Patient-Reported Outcome Measures (PROMs)

# Part 1: Summary of all evidence on the psychometric properties of the PROM

# **Part 4.1: Summary of evidence – Original Language [*insert original language here*]**

| **4.1.a Summary of content validity** | | | | | | | |
| --- | --- | --- | --- | --- | --- | --- | --- |
| **Source** | **n** | **Relevance rating** | **Comprehensibility Rating** | **Comprehensiveness Rating** | | **Overall content validity rating** | **Evidence Quality Rating** |
| **PROM Development Study (**Auth/year**)** |  |  |  |  |  |  |  |
| **Content Validity Study 1 (**Auth/year**)** |  |  |  |  |  |  |  |
| **Content Validity Study 2 (**Auth/year**)** |  |  |  |  |  |  |  |
| **Reviewer rating** | N/A |  |  |  |  |  | N/A |
| **Qualitatively pooled Result [Overall rating]** |  |  |  |  |  |  |  |

| **4.1.b Summary of other measurement properties 1/3** | | | | | | | | | | |
| --- | --- | --- | --- | --- | --- | --- | --- | --- | --- | --- |
| **Reference** (Auth/year) | **Country** | **Structural Validity** | | | **Internal Consistency** | | | **Cross-cultural validity** | | |
|  |  | **n** | **Meth qual** | **Result [Rating]** | **n** | **Meth qual** | **Result [Rating]** | **n** | **Meth qual** | **Result [Rating]** |
|  |  |  |  |  |  |  |  |  |  |  |
|  |  |  |  |  |  |  |  |  |  |  |
|  |  |  |  |  |  |  |  |  |  |  |
| **Qualitatively pooled Result [Overall rating]** | |  |  |  |  |  |  |  |  |  |
| **GRADE rating** | |  | | |  | | |  | | |

| **4.1.c Summary of other measurement properties 2/3** | | | | | | | | | | |
| --- | --- | --- | --- | --- | --- | --- | --- | --- | --- | --- |
| **Reference** (Auth/year) | **Country** | **Reliability** | | | **Measurement Error** | | | **Criterion Validity** | | |
|  |  | **n** | **Meth qual** | **Result [Rating]** | **n** | **Meth qual** | **Result [Rating]** | **n** | **Meth qual** | **Result [Rating]** |
|  |  |  |  |  |  |  |  |  |  |  |
|  |  |  |  |  |  |  |  |  |  |  |
|  |  |  |  |  |  |  |  |  |  |  |
| **Qualitatively pooled Result [Overall rating]** | |  |  |  |  |  |  |  |  |  |
| **GRADE rating** | |  | | |  | | |  | | |

| **4.1.d Summary of other measurement properties 3/3** | | | | | | | |
| --- | --- | --- | --- | --- | --- | --- | --- |
| **Reference** (Auth/year) | **Country** | **Hypothesis Testing for Construct Validity** | | | **Responsiveness** | | |
|  |  | **n** | **Methodological quality** | **Result [Rating]** | **n** | **Methodological quality** | **Result [Rating]** |
|  |  |  |  |  |  |  |  |
|  |  |  |  |  |  |  |  |
|  |  |  |  |  |  |  |  |
| **Qualitatively pooled Result [Overall rating]** | |  |  |  |  |  |  |
| **GRADE rating** | |  | | |  | | |

| **4.1.e. PROM Concordance Summary** | | | | |
| --- | --- | --- | --- | --- |
| **Reference** (auth/year) | **Comparator type**, including:   - CPS records - Child self-report - Adult retrospective self-report - Parent self-report (where parent is potential perpetrator of abuse) - Parent-as-proxy report (where the parent reports on their child’s abuse exposure) | **Population** (n) | **Result** |  |
|  |  |  |  |  |

| **4.1.f. PROM Interpretability Summary** | | | | |
| --- | --- | --- | --- | --- |
| **Distribution of scores in study population** | **Percentage of missing items (missing data in individual items)** | **Percentage of missing total scores (non-response)** | **Floor effects** | **Ceiling Effects** |
|  |  |  |  |  |
| **Scores available for relevant sub-groups?** | **Change scores available for relevant sub-groups (e.g. depressed, suicidal, criminal, revictimized respondents)?** | **Minimal Important Change (MIC)** | **Minimal Important Difference (MID)** | **Response Shift** |
|  |  |  |  |  |

# **Part 4.2: Summary of evidence – Translation 1 [*insert translation language here*]**

| **4.2.a Summary of content validity** | | | | | | | |
| --- | --- | --- | --- | --- | --- | --- | --- |
| **Source** | **n** | **Relevance rating** | **Comprehensibility Rating** | **Comprehensiveness Rating** | | **Overall content validity rating** | **Evidence Quality Rating** |
| **PROM Development Study (**Auth/year**)** |  |  |  |  |  |  |  |
| **Content Validity Study 1 (**Auth/year**)** |  |  |  |  |  |  |  |
| **Content Validity Study 2 (**Auth/year**)** |  |  |  |  |  |  |  |
| **Reviewer rating** | N/A |  |  |  |  |  | N/A |
| **Qualitatively pooled Result [Overall rating]** |  |  |  |  |  |  |  |

| **4.2.b Summary of other measurement properties 1/3** | | | | | | | | | | |
| --- | --- | --- | --- | --- | --- | --- | --- | --- | --- | --- |
| **Reference** (Auth/year) | **Country** | **Structural Validity** | | | **Internal Consistency** | | | **Cross-cultural validity** | | |
|  |  | **n** | **Meth qual** | **Result [Rating]** | **n** | **Meth qual** | **Result [Rating]** | **n** | **Meth qual** | **Result [Rating]** |
|  |  |  |  |  |  |  |  |  |  |  |
|  |  |  |  |  |  |  |  |  |  |  |
|  |  |  |  |  |  |  |  |  |  |  |
| **Qualitatively pooled Result [Overall rating]** | |  |  |  |  |  |  |  |  |  |
| **GRADE rating** | |  | | |  | | |  | | |

| **4.2.c Summary of other measurement properties 2/3** | | | | | | | | | | |
| --- | --- | --- | --- | --- | --- | --- | --- | --- | --- | --- |
| **Reference** (Auth/year) | **Country** | **Reliability** | | | **Measurement Error** | | | **Criterion Validity** | | |
|  |  | **n** | **Meth qual** | **Result [Rating]** | **n** | **Meth qual** | **Result [Rating]** | **n** | **Meth qual** | **Result [Rating]** |
|  |  |  |  |  |  |  |  |  |  |  |
|  |  |  |  |  |  |  |  |  |  |  |
|  |  |  |  |  |  |  |  |  |  |  |
| **Qualitatively pooled Result [Overall rating]** | |  |  |  |  |  |  |  |  |  |
| **GRADE rating** | |  | | |  | | |  | | |

| **4.2.d Summary of other measurement properties 3/3** | | | | | | | |
| --- | --- | --- | --- | --- | --- | --- | --- |
| **Reference** (Auth/year) | **Country** | **Hypothesis Testing for Construct Validity** | | | **Responsiveness** | | |
|  |  | **n** | **Methodological quality** | **Result [Rating]** | **n** | **Methodological quality** | **Result [Rating]** |
|  |  |  |  |  |  |  |  |
|  |  |  |  |  |  |  |  |
|  |  |  |  |  |  |  |  |
| **Qualitatively pooled Result [Overall rating]** | |  |  |  |  |  |  |
| **GRADE rating** | |  | | |  | | |

| **4.2.e. PROM Concordance Summary** | | | | |
| --- | --- | --- | --- | --- |
| **Reference** (auth/year) | **Comparator type**, including:   - CPS records - Child self-report - Adult retrospective self-report - Parent self-report (where parent is potential perpetrator of abuse) - Parent-as-proxy report (where the parent reports on their child’s abuse exposure) | **Population** (n) | **Result** |  |
|  |  |  |  |  |

| **4.2.f. PROM Interpretability Summary** | | | | |
| --- | --- | --- | --- | --- |
| **Distribution of scores in study population** | **Percentage of missing items (missing data in individual items)** | **Percentage of missing total scores (non-response)** | **Floor effects** | **Ceiling Effects** |
|  |  |  |  |  |
| **Scores available for relevant sub-groups?** | **Change scores available for relevant sub-groups (e.g. depressed, suicidal, criminal, revictimized respondents)?** | **Minimal Important Change (MIC)** | **Minimal Important Difference (MID)** | **Response Shift** |
|  |  |  |  |  |

# Part 2: Overview of PROM

# **1.a Overview of measure**

| 1.a Overview of Patient-Reported Outcome Measure (PROM) | | | | | | | | | | | | | | | |
| --- | --- | --- | --- | --- | --- | --- | --- | --- | --- | --- | --- | --- | --- | --- | --- |
| 1.a Overview | | | | | | | | | | | | | | | |
| **PROM name** [version, if applicable] | | **Main Construct**  (e.g. child abuse history, family environment, adverse childhood experiences) | | | **Target population**  (e.g. parents, children, adults) | | | | | | | **Reporting type** (*delete as appropriate)* | | | |
|  | |  | | |  | | | | | | | - Child self-report - Adult retrospective self-report - Parent self-report (where parent is potential perpetrator of abuse) - Parent-as-proxy report (where the non-offending parent reports on their child’s abuse exposure) | | | |
| 1.b PROM Feasibility | | | | | | | | | | | | | | | |
| **Mode(s) of administration**  (e.g. digital, paper-and-pencil, interview) | | | | **Copyright status** (i.e. do users need to pay a licensing fee?) | | **Cost of instrument** | | | **Regulatory Agency’s requirement for approval (**i.e. does the PROM developer require the tool to be administered by e.g. a licensed helping professional?) | | | | | | **Language** [available translations] |
|  | | | |  | |  | | |  | | | | | |  |
| **Respondent’s required physical and mental capacity (e.g. reading age)** | | | | **Required Equipment** | | | | **Total number of items** | | | | | **Time to Complete** | | |
|  | | | |  | | | |  | | | | |  | | |
| **Scope for cross-cultural use or adaptation** (*does the measure use any jargon-like terms or slang words that are difficult to translate – e.g. ‘feeling blue’?*) | | | | | | | | | | **Guidance/user manual available** (*please do a quick internet search if necessary*) | | | | | |
|  | | | | | | | | | |  | | | | | |
| 1.c PROM Structure & Characteristics | | | | | | | | | | | | | | | |
| **Subscale** [construct] | **Number of Items** | | **Response options**  (e.g. scale of 1-5? Likert scale? Visual analogue score? Multiple choice? Dichotomous?) | | | | **Range of scores/scoring** | | | | **Cut-off point** (*please specify whether the measure includes an option to binarize respondents into sub-groups, i.e. ‘abused’ vs ‘not abused’)* | | | | |
| Scale 1 |  | |  | | | |  | | | |  | | | | |
| Scale 2 |  | |  | | | |  | | | |  | | | | |
| Scale 3, … |  | |  | | | |  | | | |  | | | | |
| Sources (*please list the articles/sources where the above information was gathered*) | | | | | | | | | | | | | | | |
|  | | | | | | | | | | | | | | | |
| 1.d Summary of Available Evidence | | | | | | | | | | | | | | | |
| **Total number of articles evaluating PROM** | | | | | | | | | | | | | |  | |
| **Study on PROM development?** (delete as appropriate) | | | | | | | | | | | | | | *Yes / No* | |
| **Pilot test or Cognitive Interview Study on PROM?** (delete as appropriate) | | | | | | | | | | | | | | *Yes / No* | |
| Number of studies evaluating **Content Validity** | | | | | | | | | | | | | |  | |
| Number of studies evaluating **Structural validity** | | | | | | | | | | | | | |  | |
| Number of studies evaluating **Internal consistency** | | | | | | | | | | | | | |  | |
| Number of studies evaluating **Cross-Cultural Validity/Measurement Invariance** | | | | | | | | | | | | | |  | |
| Number of studies evaluating **Reliability** | | | | | | | | | | | | | |  | |
| Number of studies evaluating **Measurement Error** | | | | | | | | | | | | | |  | |
| Number of studies evaluating **Criterion Validity** | | | | | | | | | | | | | |  | |
| Number of studies conducting **Hypothesis Testing for Construct Validity** | | | | | | | | | | | | | |  | |
| Number of studies evaluating **Responsiveness** | | | | | | | | | | | | | |  | |
| Number of studies evaluating **Concordance** | | | | | | | | | | | | | |  | |
| Number of studies covering **Interpretability** | | | | | | | | | | | | | |  | |

# **1.b** Screenshot of measure items (*please insert screenshots of the measurement items if available, or write ‘not available’ if not*)

# Part 3: Overview of Content Validity

# **2.a Reviewer Rating**

| For further instructions on how to do a reviewer rating of content validity, see the *COSMIN Methodology for Assessing the Content Validity of PROMs, User Manual version 1.0*, **pages 51-59** |
| --- |

| **2A. Rating Content Validity - Reviewers’ rating (Face Validity)** | | | | |
| --- | --- | --- | --- | --- |
| **Domain** | | | **Criteria for a sufficient (+) rating** | Researcher Verdict |
| **Relevance** | | | | **+ / - / ?** |
| 1 | Are the included items relevant for the construct of interest? | | Reviewers consider at least 85% of the items relevant for the construct of interest. |  |
| 2 | Are the included items relevant for the target population of interest? | | Reviewers consider at least 85% of the items relevant for the population of interest. |  |
| 3 | Are the included items relevant for the context of use of interest? | | Reviewers consider at least 85% of the items relevant for the context of use of interest. |  |
| 4 | Are the response options appropriate? | | Reviewers consider the at least 85% of the response options appropriate for the construct, population, and context of use of interest. |  |
| 5 | Is the recall period appropriate? | | Reviewers consider the recall period appropriate for the construct, population, and context of use of interest. |  |
| **Comprehensiveness** | | | | |
| 6 | Are all key concepts included? | | Reviewers consider the PROM comprehensive for the construct,  population and context of use of interest. |  |
| **Comprehensibility** | | | | |
| 7 | Are the PROM instructions understood by the population of interest as intended? | |  |  |
| 8 | Are the PROM items and response options understood by the population of interest as intended? | |  |  |
| 9 | Are the PROM items appropriately worded? | | Reviewers consider at least 85% of the items and response options appropriately worded. |  |
| 10 | Do the response options match the question? | | Reviewers consider at least 85% of the response options matching the  questions. |  |
| **Pooled Reviewer Rating** | | | | |
| Domain | | | | Rating |
| Relevance (criteria 1-5, pooled^1^) | | | |  |
| Comprehensiveness | | | |  |
| Comprehensibility (criteria 9-10, pooled^2^) | | | |  |
| **^1^Guide to establishing an overall relevance rating per study** | | | | |
| **+** | | At least criteria 1 and 2 are rated + AND at least two of the other three criteria on relevance are rated + Criteria 1 and 2 (relevance for construct and population) are considered the most important criteria and therefore they need to be rated +. A maximum of 1 criterion rated – is allowed, but reviewers can also rate ± in that case. | | |
| **-** | | At least criteria 1 and 2 are rated ‐ AND at least two of the other three criteria on relevance are rated ‐ | | |
| ? | | At least two of the criteria are rated ‘?’ | | |
| ± | | All other situations | | |
| **^2^Guide to establishing an overall comprehensibility rating** | | | | |
| **+** | | Both criteria 9 and 10 are rated + | | |
| **-** | | Both criteria 9 and 10 are rated ‐ | | |
| ? | | At least one of the criteria is rated ‘?’ | | |
| ± | | One criterion is rated + and one is rated ‐ | | |

# **2.b Overall Summary of Relevance, Comprehensiveness, and Comprehensibility**

| For further instructions on how to pool information from all available sources and produce a cumulative rating of relevance, comprehensibility, comprehensiveness, and overall content validity, see the *COSMIN Methodology for Assessing the Content Validity of PROMs, User Manual version 1.0*, **pages 60-61** |
| --- |

| **2.B.1 Summary of Relevance** | | | | | | |
| --- | --- | --- | --- | --- | --- | --- |
| Criteria | | | Source | | | |
|  |  |  | PROM Development | Content Validity 1 | Content Validity 2 | Reviewer Rating |
|  |  |  | **+ / - / ?** | | | |
| 1 | Are the included items relevant for the construct of interest? | |  |  |  |  |
| 2 | Are the included items relevant for the target population of interest? | |  |  |  |  |
| 3 | Are the included items relevant for the context of use of interest? | |  |  |  |  |
| 4 | Are the response options appropriate? | |  |  |  |  |
| 5 | Is the recall period appropriate? | |  |  |  |  |
| Overall Relevance Rating (per study) | | |  |  |  |  |
| Overall Relevance Rating (pooled) | | | | | | |
|  | | | | | | |
| **Guide to establishing an overall relevance rating (across sources)^1^** | | | | | | |
| **+** | | All sources provide a ‘+’ grade for relevance  **OR**  There are no content validity studies available, but both the PROM development and reviewers’ rating are ‘+’ | | | | |
| **-** | | All sources provide a ‘-’ grade for relevance  **OR**  There are no content validity studies available, but both the PROM development and reviewers’ rating are ‘-’ | | | | |
| ± | | Some sources provide a ‘+’ grade for relevance, while others provide a ‘-‘ grade, and no explanation can be found for the inconsistency.^2,3^ | | | | |
| **Notes, taken from p. 60-61 of the *COSMIN Methodology for Assessing the Content Validity of PROMs, User Manual* :**  ^1^The OVERALL RATINGS will be sufficient (+), insufficient (‐),or inconsistent (±). An indeterminate overall rating (?) is not possible because the reviewer’s rating is always available, which will be + or – or ±. If there are no content validity studies, or only content validity studies of inadequate quality, and the PROM development is of inadequate quality, the rating of the reviewers will determine the overall ratings. Indeterminate (?) ratings for development or content validity studies can be ignored.  ^2^ If the ratings of the PROM development study, the available content validity studies, and the reviewer’s rating are inconsistent, reviewers should examine whether there is an explanation for the inconsistency. Explanations may lie in the population (e.g. differences in disease severity), the country in which the study was performed (or language version on the PROM), the year in which the PROM was developed, or the methods used in the study (e.g. study quality or patient versus professionals judgment). If an explanation is found, one should consider making subgroups of studies with similar results and draw conclusions on these subsets of studies.  ^3^ “If different RELEVANCE RATINGS were given to studies with very good or adequate quality than to studies with doubtful quality, one could consider determining the OVERALL RELEVANCE RATING based on the very good and adequate quality studies only and ignore the results of the doubtful quality studies. Otherwise studies of doubtful or inadequate quality (e.g. older studies) will always influence the overall ratings, even when multiple adequate or very good studies are available (e.g. performed later).” | | | | | | |

| **2.B.2 Summary of Comprehensiveness** | | | | | | |
| --- | --- | --- | --- | --- | --- | --- |
| Criteria | | | Source | | | |
|  |  |  | PROM Development | Content Validity 1 | Content Validity 2 | Reviewer Rating |
|  |  |  | **+ / - / ?** | | | |
| 6 | Are all key concepts included? | |  |  |  |  |
| Overall Comprehensiveness Rating (pooled) | | | | | | |
|  | | | | | | |
| **Guide to establishing an overall Comprehensiveness rating (across sources)** | | | | | | |
| **+** | | All sources provide a ‘+’ grade for comprehensiveness  **OR**  There are no content validity studies available, but both the PROM development and reviewers’ rating are ‘+’ | | | | |
| **-** | | All sources provide a ‘-’ grade for comprehensiveness  **OR**  There are no content validity studies available, but both the PROM development and reviewers’ rating are ‘-’ | | | | |
| ± | | Some sources provide a ‘+’ grade for comprehensiveness, while others provide a ‘-‘ grade, and no explanation can be found for the inconsistency.^2,3^ | | | | |
| **Notes, taken from p. 60-61 of the *COSMIN Methodology for Assessing the Content Validity of PROMs, User Manual* :**  ^1^The OVERALL RATINGS will be sufficient (+), insufficient (‐),or inconsistent (±). An indeterminate overall rating (?) is not possible because the reviewer’s rating is always available, which will be + or – or ±. If there are no content validity studies, or only content validity studies of inadequate quality, and the PROM development is of inadequate quality, the rating of the reviewers will determine the overall ratings. Indeterminate (?) ratings for development or content validity studies can be ignored.  ^2^ If the ratings of the PROM development study, the available content validity studies, and the reviewer’s rating are inconsistent, reviewers should examine whether there is an explanation for the inconsistency. Explanations may lie in the population (e.g. differences in disease severity), the country in which the study was performed (or language version on the PROM), the year in which the PROM was developed, or the methods used in the study (e.g. study quality or patient versus professionals judgment). If an explanation is found, one should consider making subgroups of studies with similar results and draw conclusions on these subsets of studies.  ^3^ “If different [COMPREHENSIVENESS] RATINGS were given to studies with very good or adequate quality than to studies with doubtful quality, one could consider determining the OVERALL [COMPREHENSIVENESS] RATING based on the very good and adequate quality studies only and ignore the results of the doubtful quality studies. Otherwise studies of doubtful or inadequate quality (e.g. older studies) will always influence the overall ratings, even when multiple adequate or very good studies are available (e.g. performed later).” | | | | | | |

| **2.B.3 Summary of Comprehensibility** | | | | | | |
| --- | --- | --- | --- | --- | --- | --- |
| Criteria | | | Source | | | |
|  |  |  | PROM Development | Content Validity 1 | Content Validity 2 | Reviewer Rating |
|  |  |  | **+ / - /?** | | | |
| 7 | Are the PROM instructions understood by the population of interest as intended? | |  |  |  |  |
| 8 | Are the PROM items and response options understood by the population of interest as intended? | |  |  |  |  |
| 9 | Are the PROM items appropriately worded? | |  |  |  |  |
| 10 | Do the response options match the question? | |  |  |  |  |
| Overall Comprehensibility rating | | |  |  |  |  |
| Overall Comprehensibility Rating (pooled) | | | | | | |
|  | | | | | | |
| **Guide to establishing an overall comprehensibility rating (across sources)** | | | | | | |
| **+** | | All sources provide a ‘+’ grade for comprehensibility  **OR**  There are no content validity studies available, but both the PROM development and reviewers’ rating are ‘+’ | | | | |
| **-** | | All sources provide a ‘-’ grade for comprehensibility  **OR**  There are no content validity studies available, but both the PROM development and reviewers’ rating are ‘-’ | | | | |
| ± | | Some sources provide a ‘+’ grade for comprehensibility, while others provide a ‘-‘ grade, and no explanation can be found for the inconsistency.^2,3^ | | | | |
| **Notes, taken from p. 60-61 of the *COSMIN Methodology for Assessing the Content Validity of PROMs, User Manual* :**  ^1^The OVERALL RATINGS will be sufficient (+), insufficient (‐),or inconsistent (±). An indeterminate overall rating (?) is not possible because the reviewer’s rating is always available, which will be + or – or ±. If there are no content validity studies, or only content validity studies of inadequate quality, and the PROM development is of inadequate quality, the rating of the reviewers will determine the overall ratings. Indeterminate (?) ratings for development or content validity studies can be ignored.  ^2^ If the ratings of the PROM development study, the available content validity studies, and the reviewer’s rating are inconsistent, reviewers should examine whether there is an explanation for the inconsistency. Explanations may lie in the population (e.g. differences in disease severity), the country in which the study was performed (or language version on the PROM), the year in which the PROM was developed, or the methods used in the study (e.g. study quality or patient versus professionals judgment). If an explanation is found, one should consider making subgroups of studies with similar results and draw conclusions on these subsets of studies.  ^3^ “If a different COMPREHENSIBILITY RATING was given to a content validity study than to the PROM development study, one could consider determining the OVERALL COMPREHENSIBILITY RATING on the content validity study only (if the content validity study is of at least adequate quality).” | | | | | | |

# **2.C Overall Rating of Content Validity**

| **2.C Rating Overall Content Validity** | | |
| --- | --- | --- |
| Rating | Criteria for establishing an overall Content Validity rating | |
| + | The RELEVANCE RATING is +, the COMPREHENSIVENESS RATING is +, and the COMPREHENSIBILITY RATING is + | |
| - | The RELEVANCE RATING is ‐, the COMPREHENSIVENESS RATING is ‐, and the COMPREHENSIBILITY RATING is ‐ | |
| ± | At least one of the ratings is + and at least one of the ratings is – | |
| ? | Two or more of the ratings are rated ? | |
| Overall Content Validity Rating | |  |

# **2.D Grading Evidence Quality**

| For further instructions on how to grade the quality of evidence on a measure’s content validity, see the *COSMIN Methodology for Assessing the Content Validity of PROMs, User Manual version 1.0*, **pages 62-64** |
| --- |

| **2.D.1 Summary of Evidence Quality** | | | | | |
| --- | --- | --- | --- | --- | --- |
| **Domain** | **Verdict** *(delete as appropriate)* | | | **Justification** (*please explain your reasoning for the action taken, in the context of the instructions provided in the notes below (section 2.D.2 & 2.D.3))* | |
| Risk of Bias^1^ | - *High Quality* - *Moderate Quality* - *Low Quality* - *Very Low Quality* | | |  | |
| Inconsistency^2^ | - *Downgrade* - *No action* | | |  | |
| Indirectness^3^ | - *Downgrade* - *No action* | | |  | |
| **Overall evidence grade** *(delete as appropriate)* | | | - *High Quality* - *Moderate Quality* - *Low Quality* - *Very Low Quality* | | |
| **2.D.2 ^1^Instructions for assessing Risk of Bias for evidence on content validity** | | | | | |
| **Evidence Quality Rating** | | **Criteria^a^** | | | |
| High Quality | | At least one content validity study of very good or adequate quality | | | |
| Moderate Quality | | At least 1 content validity study of doubtful quality | | | |
|  |  | One of the following:   - Only content validity studies of inadequate quality - no content validity studies **and** PROM development study of very good or adequate quality | | | |
| Low Quality | | One of the following:   - Only content validity studies of inadequate quality - No content validity studies **and** PROM development study of doubtful quality | | | |
| Very Low Quality | | One of the following:   - Only content validity studies of inadequate quality or - No content validity studies AND PROM development study of inadequate quality | | | |
| **2.D.3 Instructions on downgrading evidence quality due to *Inconsistency* or *Indirectness*** | | | | | |
| **Domain** | **Description** | | | | **Action to Take** |
| ^2^Inconsistency | Inconsistency refers to discrepancies in the content validity ratings between the PROM development study, content validity study (or studies), and reviewers’ rating. | | | | Downgrade the evidence by one level (e.g. from moderate quality to low quality). |
| ^3^Indirectness | Indirectness refers to cases in which the evidence is based on a study population that differs from the population for which the PROM was intended to be used. In our review, indirectness refers to situations where e.g. a child abuse measure that is intended to measure exposure retrospectively in an adult sample is used instead to measure current child abuse exposure in a child sample. | | | | Downgrade the evidence by one level (e.g. from low quality to very low quality). |
| **Source:** adapted from recommendations on page 64.  Notes, from COSMIN Guide to Step 5, p. 64  ^a^ “[H]igh quality evidence for content validity can be obtained by at least one content validity study of adequate quality, independent of the quality of the PROM development. This means that high quality evidence for content validity study can also be obtained for PROMs that were poorly developed.” | | | | | |

**Further notes re. grading the evidence on Content Validity**

**From the COSMIN guide to assessing content validity, p. 64:**

- “**Inconsistency** can occur if the ratings of the PROM development study and additional content validity studies are inconsistent or if the ratings of these studies are inconsistent with the reviewers’ ratings of the PROM. Inconsistency may already have been solved in step 3b, by making subgroups of studies with similar results and provide OVERALL RATINGS for these subgroups of studies. However, an alternative solution could be to give one OVERALL RATING, including all studies, even if the ratings per study are inconsistent, and **grade down the quality of the evidence for inconsistency.** It is up to the review team to decide which seems to be the best solution for their review.”
- **Indirectness** can occur if content validity studies are included that were performed in another population or another context of use than the population or context of use of interest in the systematic review, or if the content validity study assessed whether the PROM was valid for measuring another construct of interest than the one in the systematic review. Such studies could provide evidence on the comprehensibility of the PROM, but the evidence for the relevance and comprehensiveness may be considered indirect because this clearly depend on the construct and population of interest. In that case, it is possible **to downgrade the evidence for indirectness**.
  - “Indirectness can also occur when the PROM was developed for a target population that is not the same as the population of interest in the review. In the example above, the DASH was developed for a broader target population (musculoskeletal disorders of the upper limb) than the population of interest in the review (hand OA). If only a few patients with hand OA were involved in the PROM development one may not be sure that the items of the DASH are relevant and comprehensive for patients with hand OA. In that case, reviewers may consider to down grade the quality of the evidence from the PROM development study for indirectness.”
- **“Imprecision** is less relevant for content validity because PROM development and content validity studies concern qualitative research. Publication bias is difficult to assess because of a lack of registries for PROM development studies and content validity studies.”

# **Part 4: Overview of Other Measurement Properties**

# **Part 3a: Pooling of Results (to be completed)**

Pooling of results will be qualitative.

# **Part 3b: Grading evidence Quality**

| For further instructions on how to grade the evidence quality of studies into different psychometric properties of a measure, see the *COSMIN Methodology for Systematic Reviews of Patient-Reported Outcome Measures (PROMS) User Manual Version 1.0*, **pages 47-60** |
| --- |

| **4.b Instructions on Grading Evidence Quality** | | | | | | | | | |
| --- | --- | --- | --- | --- | --- | --- | --- | --- | --- |
| Criteria for downgrading evidence quality across 4 dimensions | | | | | | | | | |
| **Dimension** | **Grade** | | **Criteria for grading** | | | | | | **Prescribed Action** |
| **Risk of Bias** | No | | There are multiple studies of at least adequate quality, or there is one study of very good quality available | | | | | | None |
|  | Serious | | There are multiple studies of doubtful quality available, or there is only one study of adequate quality | | | | | | -1 point |
|  | Very Serious | | There are multiple studies of inadequate quality, or there is only one study of doubtful quality available | | | | | | -2 points |
|  | Extremely Serious | | There is only one study of inadequate quality available | | | | | | -3 points |
| **Inconsistency** | No | | Similar results from reviewer rating and content validity/PROM development study | | | | | | No action |
|  | Serious | | Content validity study result differs from PROM development study result | | | | | | -1 point |
|  | Very Serious | | Reviewer result differs from results of content validity and PROM development studies. | | | | | | -2 points |
| **Imprecision** | No | | Total sample size (n) >100 | | | | | | No action |
|  | Serious | | Total sample size (n) = 50-100 | | | | | | -1 point |
|  | Very Serious | | Total sample size (n) <50 | | | | | | -2 points |
| **Indirectness** | No | | PROM was evaluated using the population for which it was intended. | | | | | | No action |
|  | Serious | | PROM not used on appropriate or intended age group (e.g. Juvenile Victimization Questionnaire used on children under 10). | | | | | | -1 point |
|  | Very Serious | | PROM not used on appropriate respondents (e.g. the Parent-Child Conflict Tactics Scale – parent version used to retrospectively measure child abuse exposure in a sample of adult respondents) | | | | | | -2 points |
| 4.b Criteria for downgrading overall evidence quality | | | | | | | | | |
| **Overall Evidence Quality** | | **Points deducted** | | | | | **Quality of Evidence** | | |
|  |  | 0 | | | | | High | | |
|  |  | -1 | | | | | Moderate | | |
|  |  | -2 | | | | | Low | | |
|  |  | -3 or more | | | | | Very Low | | |
| 4.c Overall Evidence Quality Criteria for each measurement property | | | | | | | | | |
| **Structural Validity** | **Domain** | | | | **Quality grading** | **Points to deduct** | | **Researcher Verdict** | |
|  | Risk of Bias | | | |  |  | | (*please specify the resulting Quality of Evidence grade)* | |
|  | Inconsistency | | | |  |  | |  |  |
|  | Imprecision | | | |  |  | |  |  |
|  | Indirectness | | | |  |  | |  |  |
|  | **Total:** | | | | |  | |  |  |
|  | **Justification for grade** (*if necessary, briefly explain your reasoning below*) | | | | | | | | |
|  |  | | | | | | | | |
| **Internal Consistency** | **Domain** | | | | **Quality grading** | **Points to deduct** | | **Researcher Verdict** | |
|  | Risk of Bias | | | |  |  | | (*please specify the resulting Quality of Evidence grade)* | |
|  | Inconsistency | | | |  |  | |  |  |
|  | Imprecision | | | |  |  | |  |  |
|  | Indirectness | | | |  |  | |  |  |
|  | **Total:** | | | | |  | |  |  |
|  | **Justification for grade** (*if necessary, briefly explain your reasoning below*) | | | | | | | | |
|  |  | | | | | | | | |
| **Cross-Cultural Validity/ Measurement Invariance** | **Domain** | | | | **Quality grading** | **Points to deduct** | | **Researcher Verdict** | |
|  | Risk of Bias | | | |  |  | | (*please specify the resulting Quality of Evidence grade)* | |
|  | Inconsistency | | | |  |  | |  |  |
|  | Imprecision | | | |  |  | |  |  |
|  | Indirectness | | | |  |  | |  |  |
|  | **Total:** | | | | |  | |  |  |
|  | **Justification for grade** (*if necessary, briefly explain your reasoning below*) | | | | | | | | |
|  |  | | | | | | | | |
| **Reliability** | **Domain** | | | | **Quality grading** | **Points to deduct** | | **Researcher Verdict** | |
|  | Risk of Bias | | | |  |  | | (*please specify the resulting Quality of Evidence grade)* | |
|  | Inconsistency | | | |  |  | |  |  |
|  | Imprecision | | | |  |  | |  |  |
|  | Indirectness | | | |  |  | |  |  |
|  | **Total:** | | | | |  | |  |  |
|  | **Justification for grade** (*if necessary, briefly explain your reasoning below*) | | | | | | | | |
|  |  | | | | | | | | |
| **Measurement Error** | **Domain** | | | | **Quality grading** | **Points to deduct** | | **Researcher Verdict** | |
|  | Risk of Bias | | | |  |  | | (*please specify the resulting Quality of Evidence grade)* | |
|  | Inconsistency | | | |  |  | |  |  |
|  | Imprecision | | | |  |  | |  |  |
|  | Indirectness | | | |  |  | |  |  |
|  | **Total:** | | | | |  | |  |  |
|  | **Justification for grade** (*if necessary, briefly explain your reasoning below*) | | | | | | | | |
|  |  | | | | | | | | |
| **Criterion Validity** | **Domain** | | | | **Quality grading** | **Points to deduct** | | **Researcher Verdict** | |
|  | Risk of Bias | | | |  |  | | (*please specify the resulting Quality of Evidence grade)* | |
|  | Inconsistency | | | |  |  | |  |  |
|  | Imprecision | | | |  |  | |  |  |
|  | Indirectness | | | |  |  | |  |  |
|  | **Total:** | | | | |  | |  |  |
|  | **Justification for grade** (*if necessary, briefly explain your reasoning below*) | | | | | | | | |
|  |  | | | | | | | | |
| **Hypothesis Testing for Construct Validity** | **Domain** | | | **Quality grading** | | **Points to deduct** | | **Researcher Verdict** | |
|  | Risk of Bias | | |  | |  | | (*please specify the resulting Quality of Evidence grade)* | |
|  | Inconsistency | | |  | |  | |  |  |
|  | Imprecision | | |  | |  | |  |  |
|  | Indirectness | | |  | |  | |  |  |
|  | **Total:** | | | | |  | |  |  |
|  | **Justification for grade** (*if necessary, briefly explain your reasoning below*) | | | | | | | | |
|  |  | | | | | | | | |
| **Responsiveness** | **Domain** | | | **Quality grading** | | **Points to deduct** | | **Researcher Verdict** | |
|  | Risk of Bias | | |  | |  | | (*please specify the resulting Quality of Evidence grade)* | |
|  | Inconsistency | | |  | |  | |  |  |
|  | Imprecision | | |  | |  | |  |  |
|  | Indirectness | | |  | |  | |  |  |
|  | **Total:** | | | | |  | |  |  |
|  | **Justification for grade** (*if necessary, briefly explain your reasoning below*) | | | | | | | | |
|  |  | | | | | | | | |

# **Supplement 1. Guide on pooling Results**

**Extracted from the COSMIN User Manual for Systematic Reviews, pg. 32**

***Applying criteria for good measurement properties to the pooled or summarized result***

*The pooled or summarized result per measurement property per PROM should again be rated against the same quality criteria for good measurement properties (Table 4). The overall rating for the pooled or summarized result can be sufficient (+), insufficient (–), inconsistent (±), or indeterminate (?). This rating can be added to the pooled or summarized result per PROM for each measurement property in the Summary of*

*Findings Tables (Appendix 8).*

*If the results per study are all sufficient (or all insufficient), the overall rating will also be sufficient (or insufficient). To rate the qualitatively summarized results as sufficient (or insufficient), in principle 75% of the results should met the criteria. For example, for structural validity the criteria is that ‘at least 75% of the CFA studies found the same factor structure’. The criteria for hypotheses testing and responsiveness (construct approach) for summary results is that ‘at least 75% of the results should be in accordance with the hypotheses’ to rate the overall results as ‘sufficient’ and ‘at least 75% of the results are not in accordance with the hypotheses’ to rate the overall results as ‘insufficient’’.*

*If the results of single studies which can be pooled are inconsistent and the inconsistency is unexplained, the results could be pooled anyway, and this pooled result could be rated as either sufficient or insufficient, and subsequently be downgraded due to inconsistency (see also Chapter 3.1.3 and the next section). If the results of single studies which cannot be pooled (e.g. results of CFAs) are inconsistent and the inconsistency is unexplained, the overall result will be rated as ‘inconsistent’. In this case, the results are actually not summarized, and the evidence will not be graded. If the results per study are all indeterminate (?), the overall rating will also be indeterminate (?).*

# **Supplement 2. Overview on grading – Quality of Evidence**

**Extracted from the COSMIN User Manual for Systematic Reviews, pg. 32-36**

***Grading the quality of the evidence***

**Guide to GRADE rating:**

*After pooling or summarizing all evidence per measurement property per PROM, and rating the pooled or summarized result against the criteria for good measurement properties, the next step is to grade the quality of this evidence. The quality of the evidence refers to the confidence that the pooled or summarized result is trustworthy. The grading of the quality is based on the Grading of Recommendations Assessment, Development, and Evaluation (GRADE) approach for systematic reviews of clinical trials (20). Using a modified GRADE approach, the quality of the evidence is graded as high, moderate, low, or very low evidence (for definitions, see Table 5). The GRADE approach uses five factors to determine the quality of the evidence: risk of bias (quality of the studies), inconsistency (of the results of the studies), indirectness (evidence comes from different populations, interventions or outcomes than the ones of interest in the review), imprecision (wide confidence intervals), and publication bias (negative results are less often published). For evaluating measurement properties in systematic reviews of PROMs, the following four factors should be taken into account: (1) risk of bias (i.e. the methodological quality of the studies), (2) inconsistency (i.e. unexplained inconsistency of results across studies), (3) imprecision (i.e. total sample size of the available studies), and (4) indirectness (i.e. evidence from different populations than the population of interest in the review).*

***How to Apply GRADE***

*For each pooled result or for the summarized result for each measurement property per PROM, the quality of the evidence will be determined by using Table 6. If in summarizing the evidence and determining the overall rating of the pooled or summarized result for a measurement property, the results of some studies are ignored, these studies should also be ignored in determining the quality of the evidence. For example, if only the results of high quality studies are considered in determining the overall rating, then only the high quality studies determine the grading of the evidence (in this case we would not downgrade for risk of bias).*

| **Modified GRADE approach for grading the quality of evidence** | |
| --- | --- |
| **Dimension** | **Instructions for downgrading** |
| Risk of Bias | **-1 point** if Serious; **-2 points** if Very Serious; **-3 points** if Extremely Serious |
| Inconsistency | **-1 point** if Serious; **-2 points** if Very Serious; |
| Imprecision | **-1 point** if total n=50-100; **-2 points** if total n<50; |
| Indirectness | **-1 point** if Serious; **-2 points** if Very Serious; |

Taken from COSMIN user manual p. 34 (table 6)

- **On Determining Risk of Bias:**
  - “Risk of bias can occur if the quality of the study is doubtful or inadequate, as assessed with the COSMIN Risk of Bias checklist, or if only one study of adequate quality is available. The quality of evidence should be downgraded with one level (e.g. from high to moderate evidence) if there is serious risk of bias, with two levels (e.g. from high to low) if there is very serious risk of bias, or with three levels (i.e. from high to very low) of there is extremely risk of bias. In Table 7 we explain what we consider as serious, very serious or extremely serious risk of bias.”
    - - - Source: COSMIN User Manual for Systematic Reviews, p. 34

| **Instructions on Downgrading for Risk of Bias** | |
| --- | --- |
| **Risk of Bias grade** | **Criteria for grading** |
| No | There are multiple studies of at least adequate quality, or there is one study of very good quality available |
| Serious | There are multiple studies of doubtful quality available, or there is only one study of adequate quality |
| Very Serious | There are multiple studies of inadequate quality, or there is only one study of doubtful quality available |
| Extremely Serious | There is only one study of inadequate quality available |

Taken from COSMIN user manual p. 34 (table 7)

- **On Determining Inconsistency:**
  - “Inconsistency may already have been solved by pooling or summarizing the results of subgroups of studies with similar results and provide overall ratings for these subgroups. In this case, one doesn’t need to downgrade. If no explanation for inconsistency is found, the review team can decide not to pool or summarize results, and rate the results as ‘inconsistent’. In this case, no quality level for the evidence will be given. However, an alternative solution could be to rate the pooled or summarized result (e.g. based on the majority of results) as sufficient or insufficient and downgrade the quality of the evidence for inconsistency with one or two levels. The reviewers should also decide what will be considered as serious (i.e. ‐1 level) or very serious (i.e. ‐2 levels) inconsistency, because this is context dependent. It is up to the review team to decide which seems to be the best solution in each situation.”
    - - - Source: COSMIN User Manual for Systematic Reviews, p. 34-35
- **On Determining Imprecision:**
  - “Imprecision refers to the total sample included in the studies. We recommend to downgrade with one level when the total sample size of the pooled or summarized studies is below 100, and with two levels when the total sample size is below 50. Note that this principle should not be used for measurement properties in which a sample size requirement is already included in the COSMIN Risk of Bias box, i.e. content validity, structural validity, and cross‐cultural validity.”
    - - - Source: COSMIN User Manual for Systematic Reviews, p. 35
- **On Determining Indirectness:**
  - “Indirectness can occur if studies are included in the review that were (partly) performed in another population or another context of use than the population or context of use of interest in the systematic review. For example, if only part of the study population consists of patients with the disease of interest, the review team can decide to downgrade with one or two levels for serious or very serious indirectness. One can consider downgrading for indirectness for construct validity or responsiveness when the evidence is considered weak. For example when the evidence is only based on comparisons with PROMs measuring different constructs or only based on differences between extremely different groups. How to decide on what should be considered as serious or very serious indirectness is context dependent, and should be decided on by the review team.”
    - - - Source: COSMIN User Manual for Systematic Reviews, p. 35

**Determining a final Grade for evidence quality**

*To determine the grading for the quality of evidence, the concerns about the quality of the evidence should be added up. Therefore, it is helpful to consider the GRADE factors one by one by using the consecutive order as specified in Table 6.*

- ***First****, the risk of bias is considered (see Table 7). For example, when three studies are found with sufficient (i.e. ‘+’) results, but all of doubtful quality, the level of evidence will be downgraded for risk of bias from high to moderate (i.e. ‐1).*
- ***Second****, further downgrading for other factors should be considered. After risk of bias, inconsistency should be considered. If the results of the three studies in the example above are all rated as sufficient, no downgrading for inconsistency is required. Otherwise, downgrading should be considered.*
- ***Next****, the sample size should be taken into account. For example, when the sample size of the three studies together is more than 100, there will be no further downgrading. If the (total) sample size is between e.g. 50‐100, one should downgrade with ‐1.*
- ***Lastly****, the evidence could be downgraded because of indirectness. For example, consider a systematic review that focusses on pain and comfort scales for infants, and the inclusion criteria is ‘children between 0‐18 years’ because a lack of studies in infants only (i.e. below 1 year) was expected. Studies including children of all ages, including infants, may lead to downgrade the quality of evidence by one level, and studies including only children between 4 and 12 years may even lead to downgrade the quality by two levels, due to indirectness of the results. If, in our example, the three studies found all include children between 0‐4, but only very few infants, one may decide to downgrade one level (i.e. from moderate to low). In this example, the overall quality of COSMIN manual for systematic reviews of PROMs 36 the evidence is now considered as ‘low’, so the conclusion will be that there is low quality evidence that the measurement property of interest is sufficient.*
  - - - - **Source:** COSMIN User Manual for Systematic Reviews, p. 35-36
