# Supplementary material for: Measuring Violence Against Children: A COSMIN Systematic Review of the Psychometric and Administrative Properties of Adult Retrospective Self-report Instruments on Child Abuse and Neglect
Source: Trauma Violence Abuse. 2023 Jan 25;25(1):183–96. doi: 10.1177/15248380221145912 (PMC10666516; doi:10.1177/15248380221145912)
Supplement: sj-docx-6-tva-10.1177_15248380221145912 – Supplemental material for Measuring Violence Against Children: A COSMIN Systematic Review of the Psychometric and Administrative Properties of Adult Retrospective Self-report Instruments on Child Abuse and Neglect [file sj-docx-6-tva-10.1177_15248380221145912.docx]

Supplementary Material: Review Decisions

*Title and abstract screening*

Inclusion criteria:

1. All articles which are a psychometric evaluation study looking at validity, factor structure or reliability of a child abuse measure are automatically included
   1. Child abuse measures include the ones that are obviously child abuse measures but also some more obscure ones such as the Life Events Checklist, Childhood Trauma Questionnaire etc. or parenting measures of harsh parenting i.e. Alabama Parenting Questionnaire or Parental Discipline Inventory and those that measure child abuse potential AAPI and CAPI
2. All articles which mention the use of a child abuse measure by name i.e. ACE, CTQ, ICAST and report on the correlations specified in the protocol i.e. depression, anxiety, suicidal ideation, self-harm problem behaviour and drug use, violence revictimisation, concurrent violence types or associations between two different child abuse measures
   1. Child abuse measure must be self-report - it can be historic (adults report on their childhood experiences) or current (children/adolescents) or parents current report
3. Reviews on the psychometric properties of child abuse measures/harsh parenting measures or childhood trauma checklists
4. Evaluation of an intervention which prevents/reduces child abuse if they use a self-report measure
   1. Reasoning: Trials will always have to report some psychometrics in their methods section. Interventions must have child abuse or child abuse potential or corporal punishment as an outcome i.e. child behaviour or parenting stress as outcomes are NOT included.
   2. Reviews of interventions are excluded; only primary studies are included.

Include at title and abstract screening stage:

1. Cases where there is a measure stated by name but there are no correlates mentioned and there is a chance that there are psychometrics anywhere in the text

Exclusion criteria:

1. Studies where child abuse is measured with agency records
2. Use of non-self-report measure to asses child abuse (i.e. agency or sexually abused children who are in therapy)
3. Studies which use self-report of social workers or other professionals
4. Studies which use self-report child abuse measures and report on correlates that are not pre-specified i.e. cognitive development or obesity or heart disease or education etc.
5. Reviews or background chapters
6. Qualitative studies
7. Name of questionnaire is not stated but the right correlates are given
8. Articles which do not mention child abuse
9. Name of questionnaire is not stated and study reports neither on psychometrics nor on correlates
10. Study reports on attitudes or perceptions about abuse

Clarifications during title and abstract screening:

1. Studies that mention the “ACE study” - Include as they likely used the ACE questionnaire
2. Studies in which CAPI is mentioned as a questionnaire but filled out as day-care staff, not parents (defined as a population in protocol) Exclude
3. Study on child and adolescent neglect which used the Adolescent Abuse Inventory Include
4. Studies where the abstract aimed “To develop a psychometric instrument to evaluate psychological processes associated with institutional (child) abuse and coping strategies used to deal with such abuse” Exclude

*Full-text screening*

1. If number of experiences reported by each participant was summed for a total ACEs score from 0 to 8 or similarly another scale like the CTQ/JVQ, even if the questionnaire includes a range of questions not about abuse – Include if psychometrics are provided
2. When questions on the ACE or other measures are grouped into scales (e.g., there are two questions on emotional abuse which then make the "child abuse scale") and relevant metrics for correlates are provided: Include while flagging that ACE/CTQ measure other things as well as child abuse and that we can't be certain that the correlations are driven by abuse
3. Study includes relevant correlates, but the correlations of individual items are provided – Exclude
4. When studies report ‘recycled’ psychometrics – such as alpha/kappa values – from a previous study sample – Exclude
5. When studies cut a child abuse measure down to a few questions and only one item is related to abuse - Exclude
6. When studies using the ACE questionnaire report correlations between each item on a type of childhood adversity with one of our relevant correlates e.g. correlates between parental alcoholism and depression and then correlates between child abuse and depression – Exclude as it's neither a dichotomous variable of ACEs vs no ACEs or ACEs +4 vs no ACEs with our pre-specified outcomes
7. Studies not found online or found but not accessible – Exclude as unavailable or not accessible
8. Studies accessible in a non-English language version – Exclude
9. Studies testing hypotheses with one of the specified outcomes, using individual scores or splitting samples into abused/non-abused – Include
10. List of unacceptable correlates:
    1. PTSD
    2. Relationship with spouse/family/friends
    3. Quality of life
    4. Eating disorders
    5. Self-capacities- inner connection; affect tolerance; self-worth.
    6. Adult attachment
    7. Self-injurious behaviour
    8. Borderline Personality Disorder
11. Clarifications on specified correlates:
    1. Problem behaviour - delinquency and criminal activity or violence perpetration.
    2. Measuring correlates:
       1. Studies included only if specified correlates are measured using a validated questionnaire
       2. Medical diagnoses included, but only if a medical diagnosis is specifically for one of the specified outcomes i.e. depression or anxiety as opposed to a general diagnosis of ‘poor mental health’

*Reviews included*

1. Reviews where no psychometric values are reported in text or tables – Exclude
2. Reviews which include only child abuse potential measures - Exclude
3. Reviews where some psychometrics from primary studies are included, usually in the form of a table
   1. Double check Dropbox to see if studies in the review are in our included studies. If they are included, no additional action needed. If they are not, track down the primary studies and do a full-text review.
   2. Caveats of additional full-text review:
      1. Where the reviews report that no psychometric information is provided in a primary study that is not already included in Dropbox – No need to track down the original study
      2. Where the reviews report that psychometric information is provided in a primary study that is not already included in Dropbox – Track down and do a full-text review of the primary study

*Process of developing data extraction forms*

Materials used for developing the data extraction forms were taken from the example data extraction grids provided by COSMIN and different checklists and evidence criteria provided in the User Manual. We decided to use Word to develop the data extraction forms in, as COSMIN forms were available both in Word and Excel, and it was easier to make the extraction grid user-friendly in Word.

In line with the multiple levels of extraction and analysis in a COSMIN review, namely: (1) evidence and RoB ratings for individual studies on each measurement property, followed by 2) evidence and RoB ratings for the evidence base of each PROM overall, followed by 3) a top-level overview of PROMs to find the 'best' one), we developed multiple different data extraction grids.

The first level consists of extraction grids for individual articles that look into a PROM’s different measurement properties. One extraction grid can be used for articles that look into content validity, since it is a much lengthier process, and another extraction grid for articles that look into all other properties of a measure (structural validity, reliability, etc). The idea was that when using these grids, we will have everything we need right in front of us in the document to do an evidence rating and a risk of bias rating as extraction happens. This was a time-consuming process that yielded lengthy documents, but we prioritised this in order to ensure that all reviewers are clear about what they are doing. We also developed an initial grid detailing the meanings of each measurement property familiarise any new reviewers who weren't familiar with COSMIN.

The second level is an extraction grid to contain all information on the evidence base of each individual PROM. This can be populated as soon as each extraction grid on individual articles/studies is finished. This level has information on implementing GRADE.

The third level is a grid of tables on an overview of all PROMs, to help with the stage of selecting and recommending a PROM.

*Data extraction decisions*

1. General:
   1. Studies on child abuse prevalence – data not extracted unless psychometrics of measure provided, or other outcomes reported e.g. hypothesis testing with an outcome of interest
   2. Differentiating between the hypothesis testing table and the responsiveness table: If the study has an intervention and the outcome is child abuse or change in child abuse score, then this goes into the responsiveness boxes of the table. The reason for this is that we are interested in responsiveness only for changes in child abuse scores, nothing else (so not mental health etc.). If the study that looks at associations between child abuse score and a pre-determined outcome specified in the protocol (at the same time point or a later time point), then you would be filling in the hypothesis testing box. Use Part 3 checklist to determine which box is to be filled out.
   3. Definition of child or childhood exposure to violence: This would be violence experienced before the 18^th^ birthday. It’s NOT important how old the respondent is when they fill in the questionnaire - it’s important at what age the questions measure the violence.
   4. Sensitivity/specificity and positive/negative predictive values - reported under 'Concordance'.
   5. Associations between child abuse self-report and maternal report of abuse risk: If two respondents report on the same outcome – extracted in Concordance. If it is mother’s risk of being abused – excluded. If it is mother’s risk of perpetrating abuse – excluded
   6. Populations: Studies with individuals under the age of 18 – classified as child self-report and extracted and summarised accordingly. Studies with individuals under the age of 18 – classified as adult self-report and extracted and summarised accordingly Studies with a mixed sample – assess if majority are under or over 18 and classify accordingly.
2. Content Validity and PROM Development:
   1. If a study is adapted, then it is technically a new PROM so the PROM development boxes should be filled in e.g. culturally adapting the ICAST-R measure in Indonesia to the context
   2. If it is a pilot study in a different sample to the study development then it would be content validity e.g. testing the adapted ICAST-R with young people in Indonesia
3. Structural Validity:
   1. If a study reports loadings values for EFA, but no CFI/TLI/RMSEA/SRMR values – a rating of – is given as criteria for '+' is not met
   2. For PCA analysis, studies which report percent of variance and eigenvalues but none of the fit indices - a rating of – is given as criteria for '+' is not met
   3. For IRT analyses, studies which haven't carried out analysis of local independence or provided evidence of monotonicity - a '?' rating is given
   4. Studies which mention they used varimax rotation, but no additional info is provided – noted as a minor flaw and downgraded accordingly in RoB
   5. When studies do a PCA and report factor loadings and eigenvalues, acceptable range for such values – Eigenvalues help in reducing the dimensions (factors) and generally loadings of more than 0.3 is considered to be acceptable
4. Hypothesis testing:
   1. We assumed that there will be significant relationships between child abuse exposure and the correlate of interest, in the expected direction - i.e. higher emotional abuse = higher anxiety or depression.
   2. When a hypothesis is stated explicitly - in the box on author's hypotheses, we quoted them when an explicit hypothesis is mentioned or wrote that a positive relationship is explicitly mentioned
   3. If the study claims that one of its aims is to test the existence of a relationship between child abuse and a correlate, but no explicit direction of the relationship is mentioned - we paste the study aim verbatim into the box (or briefly summarise - whichever would be faster) and give the study a + rating if any of the relationships are positive
   4. Rating given if a hypothesis is not stated explicitly – studies are not penalised for not stating hypothesis i.e. inadequate ratings are NOT given solely due to this reason
   5. When a study looks at multiple specified correlates and only some relationships are positive - + rating, because expecting every single specified correlate to have a significant relationship with every single subscale of a measure in a study is a very high bar to set
   6. When a study looks at multiple specified correlates and only one relationship is positive – ‘-‘ rating because only a minority of relationships are in accordance with hypotheses
   7. When a study looks at multiple specified correlates and none of the relationships are positive – ‘-‘ rating
   8. When studies carry out hypothesis testing and do not report psychometrics of comparison PROM, even if assumable that the comparison PROM has good properties in populations similar to the study population – studies are penalised in the RoB for not reporting this
   9. Is the Parental Acceptance-Rejection Questionnaire, which measures hostility/aggression among other parental behaviours, a valid comparison measure – Yes, considered as a valid comparison PROM
   10. When studies measure a specified correlate using a validated questionnaire, but this is filled out by a teacher or another respondent – Included and extracted into the hypothesis testing box
   11. When studies report alphas reported for comparison PROM, but they are not all above 0.70 – penalised in RoB assessment as comparison PROM does not have sufficient measurement properties
   12. Studies on discriminative validity – Include and extract only if the difference on the basis of which two groups are compared is one of the specified outcomes
   13. When studies measure a specified correlate using items from a questionnaire that borrows items from validated scales but is not validated itself – Exclude, and not extracted as this is not a valid comparison PROM
5. Internal Consistency:
   1. If a study reports a Cronbach’s alpha for a PROM but this is not the primary goal of the study- Yes, extracted into the internal consistency box
   2. Alphas are satisfactory for all but a single subscale of a measure – +/- rating with a footnote in the overview tables to mark every time that the alphas have been satisfactory for every subscale but the physical neglect one. Rating is given because not all alphas are above 0.70. But this is noted in the overview table.
   3. When the ‘at least low evidence for structural validity' criterion is not met from that study or another study - ? rating is given. When only one study is carried out for a PROM with no evidence of structural validity - ? rating is given
   4. When studies use a scale which has clear subscales but they've only calculated Cronbach alphas for the whole scale – a rating of – is given because the psychometrics available are not for the measure as intended
   5. Range of acceptable values for KR-20 to report internal consistency - same as for alpha, greater than .70
   6. Studies briefly report the ranges for internal consistency e.g. alpha values are between X and Y for the subscales without reporting the values for each subscale – Fill in the internal consistency box, state that it is unclear which subscale the alphas refer to and highlight this as an additional limitation under RoB
   7. When studies use only one subscale and reports alpha values for this subscale, but collects data from participants on other subscales – Noted in the overview table that the alpha values are only for the one subscale
6. Reliability:
   1. When in test-retest reliability analyses, studies’ time interval is longer than the 2 weeks recommended by COSMIN –
      - Studies penalised if they 1) had an unrealistically short interval period of 1-2 days or 2) had a long interval period for a construct that could very well change over time e.g. if there was a long interval period of 6 months+ and the measure was of parent's use of corporal punishment, CP tends to decline as children get older and that the actual construct being measured is highly likely to change.
      - But if we are asking grown adults about early adverse experiences in childhood - a construct that is retrospective and by definition cannot be changed - at test periods that are 6 months apart, studies are not penalised.
   2. Studies which have reported ICC values but not described the model/formula – an ‘adequate’ rating is given in RoB
   3. Studies which report the k coefficient as evidence of temporal stability – extracted as test-retest reliability and included in the reliability section
   4. When studies do not report information on test conditions in a test-retest reliability analysis, such as type of administration and conditions – marked as adequate in the RoB and noted reasons
   5. Criteria to determine if participants were stable in the interim period in a test-retest reliability analysis. For adult retrospective reports, sufficient to say that participants had not suffered any abuse in the interim. For child self-report or parental self-report we would want to know more about whether their caregiving situation has changed, etc.
   6. Other reliability values - Jo ̈reskog’s Rho included (same as Raykov’s Rho)
   7. Studies which report a test-retest coefficient but no ICC – downgraded accordingly in RoB
7. Measurement Invariance:
   1. Study which recruited parents of multiple ethnicities and reported scaled scores on the CTQ for each ethnicity, but key sample characteristics like age, sex etc. are provided for the sample as a whole, not for parents of each ethnicity – Exclude as no measurement invariance was conducted

*Overview ratings*

1. Inconsistency standard: Most measures do not have any content validity evidence, and for the inconsistency standard, the criteria for downgrading evidence quality depends on the differences between reviewer rating and PROM development/content validity study. To address this, a ‘not applicable’ note is made
